# Quantitative methods demonstrate that environment alone is an insufficient predictor of present-day language distributions in New Guinea

**DOI:** 10.1371/journal.pone.0239359

**Published:** 2020-10-07

**Authors:** Nicolas Antunes, Wulf Schiefenhövel, Francesco d’Errico, William E. Banks, Marian Vanhaeren

**Affiliations:** 1 Römisch-Germanisches Zentralmuseum (RGZM), Leibniz-Forschungsinstitut für Archäologie, Mainz, Germany; 2 CNRS, MCC, De la Préhistoire à l’Actuel: Culture, Environnement et Anthropologie (PACEA), Univ. Bordeaux, Pessac, France; 3 Department of Archaeogenetics, Max Planck Institute for the Science of Human History, Jena, Germany; 4 Max-Planck-Institute for Ornithology, Seewiesen, Germany; 5 SFF Centre for Early Sapiens Behaviour (SapienCE), University of Bergen, Bergen, Norway; 6 Biodiversity Institute, University of Kansas, Lawrence, Indiana, United States of America; University of Edinburgh, UNITED KINGDOM

## Abstract

Environmental parameters constrain the distributions of plant and animal species. A key question is to what extent does environment influence human behavior. Decreasing linguistic diversity from the equator towards the poles suggests that ecological factors influence linguistic geography. However, attempts to quantify the role of environmental factors in shaping linguistic diversity remain inconclusive. To this end, we apply Ecological Niche Modelling methods to present-day language diversity in New Guinea. We define an Eco-Linguistic Niche (ELN) as the range of environmental conditions present in the territory of a population speaking a specific language or group of languages characterized by common language traits. In order to reconstruct the ELNs, we used Papuan and Austronesian language groups, transformed their geographical distributions into occurrence data, assembled available environmental data for New Guinea, and applied predictive architectures developed in the field of ecology to these data. We find no clear relationship between linguistic diversity and ELNs. This is particularly true when linguistic diversity is examined at the level of language groups. Language groups are variably dependent on environment and generally share their ELN with other language groups. This variability suggests that population dynamics, migration, linguistic drift, and socio-cultural mechanisms must be taken into consideration in order to better understand the myriad factors that shape language diversity.

## Introduction

The uneven distribution of languages across the globe has interested researchers for decades, e.g. [1–10]. Cavalli-Sforza and colleagues [3,11] showed that a strong correlation exists between linguistic and genetic phyla, which they linked to prehistoric patterns of human migration. Others (e.g. [12]) have linked linguistic diversity to linguistic drift, a notion coined by Sapir [13], which refers to the process by which different linguistic elements, such as cognates and syntax, randomly diverge. More recent studies focus on the role of adaptation in linguistic diversity [8,14]. Languages are thereby seen as being adaptive to specific niches determined by the social, technological, physical and ecological aspects of the environment in which they are learned and used [8,14,15]. Especially intriguing is the correlation, stressed in several studies [4,9,10,16], between language distributions and environmental variables. They mainly reflect the finding that the highest linguistic diversity exists in tropical areas and gradually declines towards polar regions [4,9,10]. They tie this variation to global environmental gradients, such as the biodiversity gradient [17] or the mean growing season (GS) [4], which show similar latitudinal patterns, or to topographic complexity correlated with habitat diversity [16,18,19] and geographic isolation [10]. The main argument is that the more resource-abundant an environment, the more linguistic diversity it will sustain. Poor or unpredictable environments would lead to larger linguistic territories [4], which would reflect the need of human groups to maintain mutual intelligibility over large distances to promote cooperation and access to sufficient resources. However, despite considerable research efforts and numerous correlations found between language distributions and respectively variables of the natural environment, human subsistence strategies and socio-cultural organization [20], no consensus exists on the determining factor or combination of factors explaining present-day language distributions [8,9,16,21–23].

Here we apply Eco-Linguistic Niche Modelling (ELNM), an approach derived from Ecological Niche Modelling (ENM) [24], to test the correlation between language distribution and environment for mainland and island New Guinea (NG) (see Methods and Text 1 in S1 File). Due to its environmental heterogeneity, its great biodiversity [22], and because this large island has one of the highest levels of linguistic diversity in the world [7,25], NG (i.e. both halves of the island west and east of the political border between Indonesian NG and Papua NG) represents an ideal case study. Furthermore, ELNM provides a geographically-based record of language diversity against which other cultural variables and additional environmental factors (e.g. soil, vegetation, and parasite loads such as malaria) can be evaluated.

ELNM overcomes two main problems present in previous studies investigating the link between linguistic diversity and environment (e.g. [4,10,18]). First, languages and environmental variables typically are noted by country (e.g. [4]), island (e.g. [5]), culture area (e.g. [26]), or map grid cell (e.g. [10]) instead of by geographic area characterized by a homogeneous environment. Prior studies may therefore have been biased by approximations and mismatches in their environmental data-sets. Second, different environmental determinants were used individually to test the link between environmental and linguistic variability, the latter being represented by a defining factor equally valid for all languages at the same time. ELNM simultaneously takes into account a range of environmental variables and allows one to test their influence on each linguistic group independently, i.e., without assuming that environmental variables influence all linguistic groups in the same way. Humans represent an extremely adaptive species and it can be expected that the dependence on environment of linguistic groups may differ as the speakers of each group will have a specific package of subsistence strategies and socio-cultural practices. Also, in contrast to studies that examine languages and cultures globally, our study provides a detailed geographic analysis of well-defined groups of languages at a regional scale.

## Results

**Fig 1:**
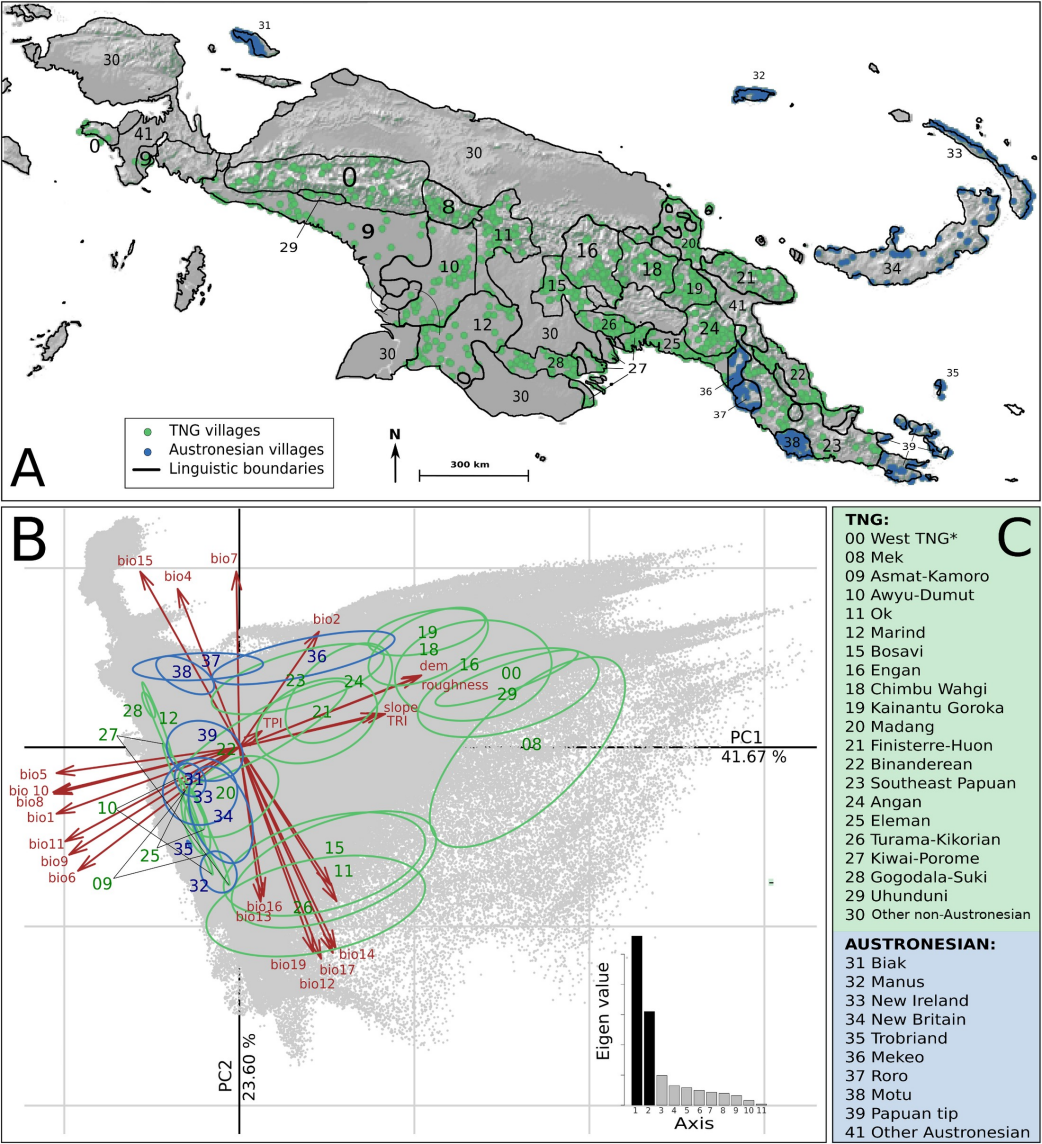
Map of New Guinea languages groups and principal component analysis. (A) Linguistic areas (Polygons) of Trans New Guinea (TNG) and Austronesian language groups (identified by index numbers given in C; * includes groups 1–7, see Materials and Methods.) and location of villages (dots) used as occurrences for Eco-Linguistic Niche Modeling; map background generated by using R::Raster—CRAN Repository, public domain software. (B) Principal Component Analysis of Eco-Linguistic Niches of New Guinea language groups and Eigen values of the most explanatory axis. Red arrows show environmental variable contributions. Environmental variable codes are explained in Table 2. Ellipses represent the inertia distributions for groups belonging to TNG (green) and Austronesian (blue) language groups. Grey background represents the available environment in New Guinea.

Eco-Linguistic-Niches (ELNs)—i.e. the range of geographic and environmental parameters that characterize a linguistic territory—were modelled for 29 top-level linguistic groups of NG (see Methods), including 9 Austronesian language family groups and 20 Trans-New Guinea (TNG) language family groups (identified by index numbers given in Fig 1, S1 Fig., Text 2 in S1 File).

The applied consensus method (see Methods) effectively allows one to calculate a best-fit ELN model from the 10 most commonly used predictive algorithms in ENM for each of the 29 linguistic groups present in the studied region. Comparisons of geographic distributions and ecological space positions of ELNs with actual linguistic areas of the 29 modelled groups show that: 1) the territory of a linguistic group only rarely corresponds to the territory of the predicted ecological niche (only seven cases); 2) ELNs are variably reliant on at least six different sets of environmental parameters; 3) half of the linguistic groups share their ELN with at least one other linguistic group; 4) language diversity within shared ELNs is extremely variable as they number from 1 to 107 languages, 1 to 7 linguistic groups, and one or both of the two modelled linguistic families (Table 1); and 5) although areas of low ecological risk yield a higher number of top-level linguistic groups, they do not yield a higher number of languages.

**Table 1:** Eco-Linguistic Patterns (ELPs) identified according to environmental space position and geographical distribution of the modeled Eco-Linguistic Niches (ELNs). Index labels (id) for linguistic groups are given in Fig 1. Number (N) of languages counted from the Glottolog database [27]. Geographical regions are indicated on the map in Fig 6.

ELP label	Geographical Region	Linguistic Group(s) id(s)	Linguistic Family	N Group(s)	N Language(s)
ELP 1	Highlands	00, 08, 16, 18, 19, 24, 29	TNG	7	95
ELP 2	Central NG	11, 15	TNG	2	20
ELP 3	Southern NG	12, 28	TNG	2	6
ELP 4	South-Eastern NG	23, 36, 37, 38	TNG, Austronesian	4	39
ELP 5	Northern NG+ Bismarck Archipelago	33, 34	Austronesian	2	59
ELP 6	South Papuan Basin	10	TNG	1	18
ELP 7	Huon Peninsula	21	TNG	1	61
ELP 8	Sepik-Ramu Basin	20	TNG	1	107
ELP 9	South-Eastern NG + South Papuan Basin	22	TNG	1	13
ELP 10	Turama, Kikori, Purari River Basins	26	TNG	1	3
ELP 11	North-Eastern part of the Gulf of Papua	25	TNG	1	5
ELP 12	Gulf of Papua	27	TNG	1	6
ELP 13	South-Western NG	09	TNG	1	11
ELP 14	Biak Island	31	Austronesian	1	1
ELP 15	Manus Island + Central NG	32	Austronesian	1	23
ELP 16	Trobriand Islands	35	Austronesian	1	1
ELP 17	Papuan Tip	39	Austronesian	1	43

### Geographic distribution of Eco-Linguistic Niches (ELNs)

**Fig 2:**
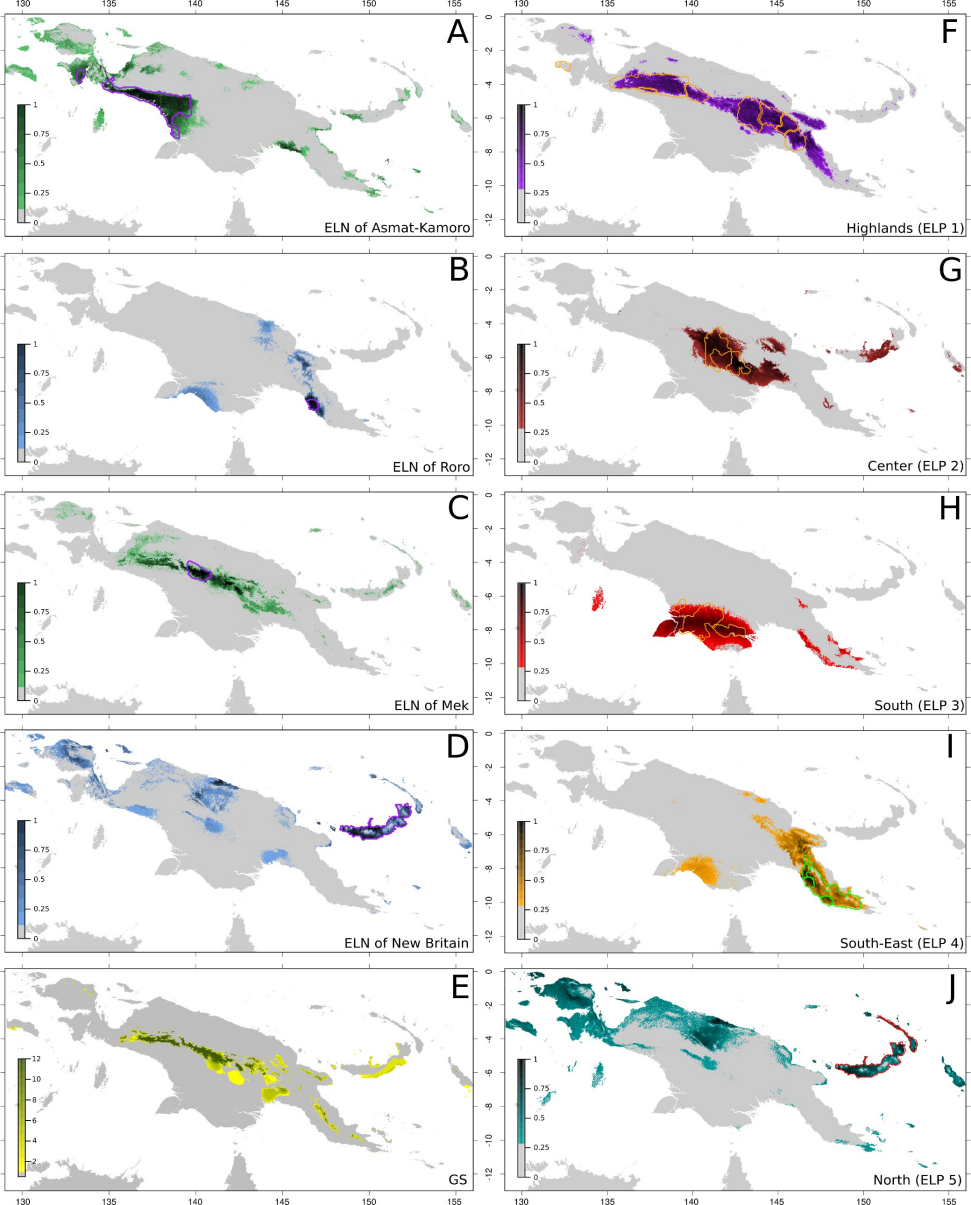
Examples of four Eco-Linguistic Niches (ELNs), Growing Season (GS), and Eco-linguistic patterns (ELPs). (A) Asmat-Kamoro, (B) Roro, (C) Mek, and (D) New Britain language groups ELNs. Colour shades reflect probability of niche presence. Green colour is used for Trans New Guinean ELNs and blue for Austronesian ELNs. The purple lines delimit linguistic areas. (E) Map of New-Guinea with GS values calculated according to the ecological risk formula (see Materials and Methods). Coulour scale indicates the length of the GS in months. (F-J): ELPs of New-Guinea. Each pattern (Highlands, Center, South, South-East and North) corresponds to the sum of similar Eco-Linguistic Niches according to their environmental space positions and their geographical distribution. Colour shades indicate prediction probabilities. Polygons indicate linguistic areas of modelled linguistic groups. The Highland pattern (F) includes from West to East the West Trans New Guinea, Uhunduni, Mek, Engan, Chimbu-Waghi, Kainantu-Goroka and Angan language groups. The Center pattern (G) includes the Ok and Bosavi, the South pattern (H) the Marind and Gogodola-Suki, the South-East pattern (I) the Mekeo, Roro, Motu and the South-East Papuan, and the North pattern (J) New Britain and New Ireland. Map background generated by using R::Raster—CRAN Repository–public domain software.

Each of the 29 modelled ELNs has a different geographic extent (Table 1 in S1 File). When ELNs are compared to linguistic areas, two types of cases can be distinguished: Either the geographic distribution of the ELN is larger than that of the linguistic area (Fig 2C and 2D) or it largely coincides with the linguistic area (Fig 2A and 2B). Most of the modelled ELNs (22 of the 29 linguistic groups) correspond to the former.

In both cases, ELNs can present discontinuities in their geographic distribution, i.e. they include territories that are disconnected from the core region around the respective linguistic area. Typically, in ENM of terrestrial mammals, these distant areas are considered inaccessible (be it because of dispersal barriers to or of sister-species in those areas) and therefore are not taken into account [28]. However, it should be pointed out that the territories in which Austronesian languages are spoken across the Indian and Pacific Oceans, from Madagascar to Polynesia, almost always represent coastal/island habitats, which reflect Austronesian maritime lifeways [29]. They are, on the one hand, separated by large water bodies and, on the other hand, potentially connected by seafaring travel. If maritime connections are taken into account, i.e. when overseas territories of the predicted ELNs can be considered accessible, only four linguistic groups remain cases wherein the geography of ELNs and the linguistic area correspond: Austronesian Trobriand (35) and Motu (39) language groups and two TNG groups, Asmat (09) and Eleman (25).

Geographic overlap between ELNs is observed for most language groups, but non-overlapping ELNs also exist. Similar geographic distributions are observed for language groups occupying, respectively, the Western Highlands (00, 08, 16, 18, 29), the Eastern Highlands (19, 21, 22, 24), the South (12, 28) and the South-East (36, 37). In contrast, no major geographic overlap in ELNs occurs in the four mentioned linguistic groups for which the geographical extends of the ELN and the linguistic area correspond.

### Ecological space positions of ELNs

A Principal Component Analysis (PCA) of the ELNs of 20 TNG and the 9 Austronesian language groups reveals six clusters within the available ecological space of NG (Fig 1). The first two components of the PCA comprise 65% of the variability (Fig 1). The first axis (41.67%) represents topography and temperature. The second axis (23.6%) mainly reflects precipitation. The first axis separates the language groups 00, 08, 16, 18, 19, 29 on the one hand and 09, 10, 12, 25, 27, 28, 31–34, 35, 37–39 on the other. At the intersection of these two axes, language groups 11, 15, 20–24, 26, 36 are present. According to the weight of the different environmental variables, the first cluster is characterized by topographic variables (altitude, roughness, terrain ruggedness index and slope) as well as annual and diurnal temperature range, the second by other variables related to temperature.

**Fig 3:**
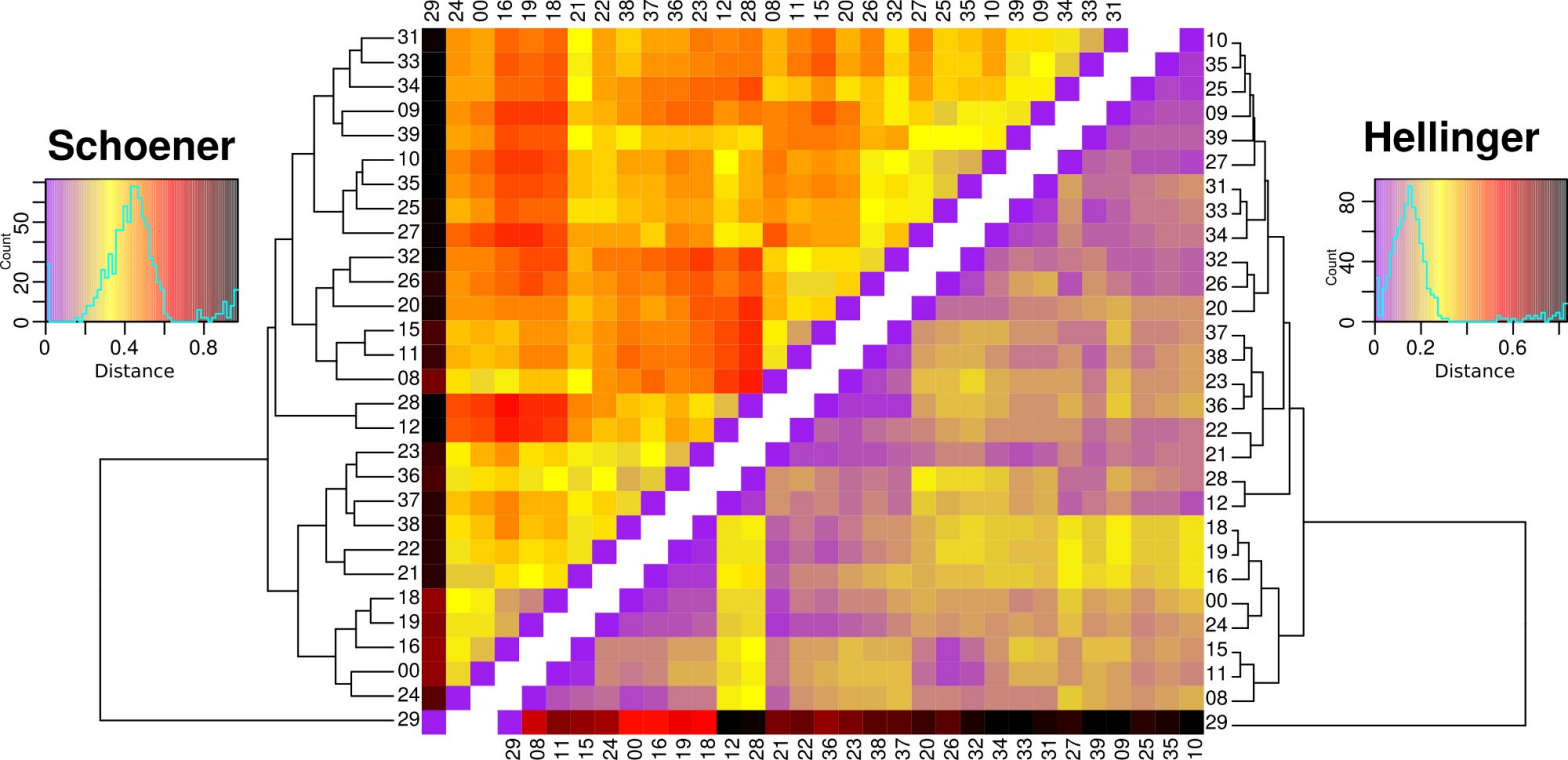
Heatmap of Eco-Linguistic Niche environmental distances computed according to the Schoener’s D and Hellinger’s I overlap scores. Distance corresponds to 1 –the overlap score (Table 2 in S9). Values vary between 0 and 1. Low values (purple) correspond to strong overlaps and environmental similarity, high values (dark red) to marked environmental differences. Numbers correspond to the index numbers of language groups given in Fig 1.

**Fig 4:**
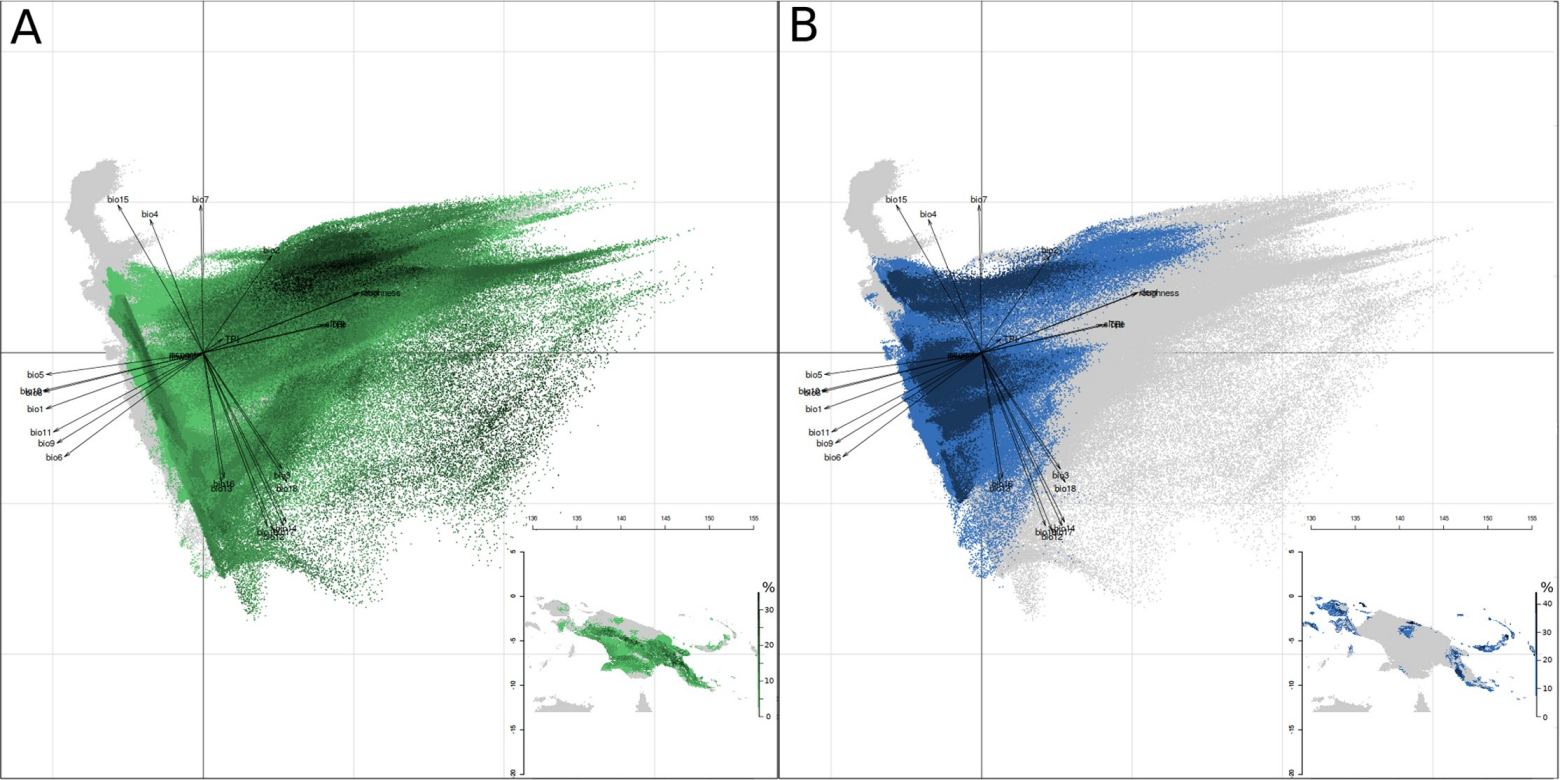
Principal component analysis and map of New Guinea Eco-Linguistic Niches. (A) Trans New Guinea language family, (B) Austronesian language family. Dots represent the distributions of ELN predictions, and the colour shades depict the number of languages. Grey background corresponds to the available environment in New Guinea. Map background generated by using R::Raster CRAN Repository–public domain software.

**Fig 5:**
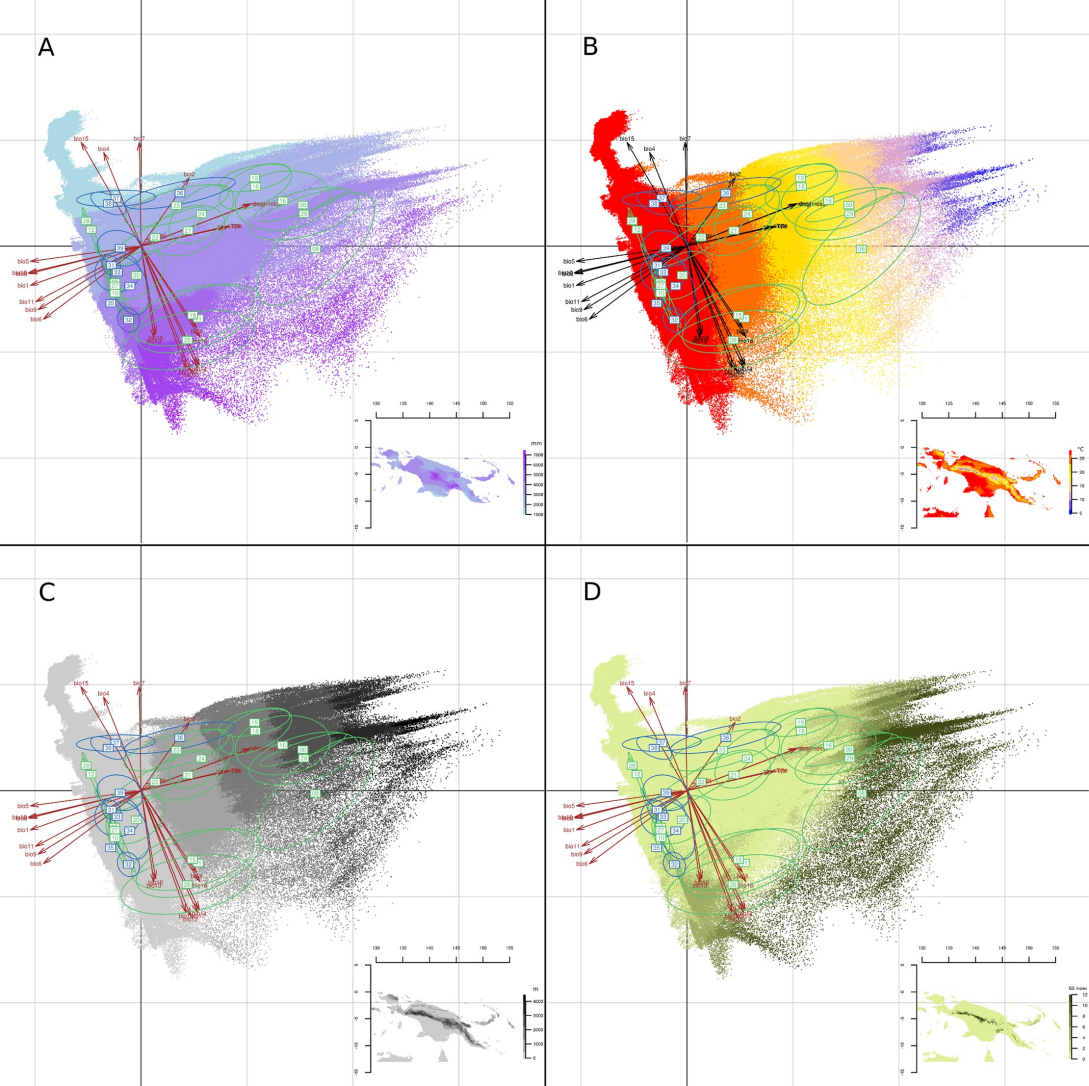
Principal component analysis of New Guinea language groups’ Eco-Linguistic Niches. Arrows show environmental variable contributions. Environmental variable codes are explained in Table 2. Ellipses represent the inertial distributions for groups belonging to Trans New Guinea (green) and Austronesian language groups (blue). Background colour scales correspond to the sum of annual precipitation (A), annual mean temperature (B), altitude (C) and Growing Season (D). Map background generated by using R::Raster CRAN Repository–public domain software.

The second axis splits the language groups located in the middle of axis 1 into three clusters. Language groups 11, 15 and 26 are clearly separated from groups 23, 36 and the remaining groups, i.e. 20–22 and 24, have values closer to zero. The cluster of groups 11, 15, 26 is strongly influenced by precipitation and that of groups 23 and 36 by precipitation seasonality. The second axis also separates groups 37 and 38, and groups 12 and 28, with niches determined by specific seasonality values for rainfall and temperature. It is noteworthy that the nine Austronesian language groups are split into three different ecological spaces. The first space (including groups 31–34 and 39) overlaps with TNG language ecological space positions but shows a wider range along axis 1. In the second ecological space (including groups 36–38), two of the three Austronesian groups do not overlap with any TNG ELN. This is also the case for the single language group (35) in the third ecological space. Hierarchical clustering of Schoener’s D and Hellinger’s distance values reveal clusters (Fig 3, Table 2 in S1 File) almost identical to those identified by the PCA. Exceptions concern Turama-Kikorian, which is clearly separated from Bosavi and Ok, as well as Uhunduni. This may be due, at least for the former language group, to the fact that the ELNM was conducted with a small sample of occurrences. The separate positioning of the former three language groups must, however, be explained by information provided by the third axis and subsequent axes of the PCA. These distance values also allow one to identify the position of the ELNs with values close to zero on the 1st and 2nd axis of the PCA. Group 24 shows proximity to the groups dependent on temperature amplitude and altitude (i.e. groups 00, 08, 16, 18, 19, 29). Group 20 is linked to groups 32 and 26 but shows a greater distance value suggesting it constitutes a separate entity. Finally, groups 21 and 22 cluster together. When the ecological space positions of Austronesian and TNG linguistic families are considered separately, their distinctiveness becomes evident (Fig 4). The distribution of the Austronesian linguistic family is parallel to the second axis of the PCA, which mainly reflects the intensity and seasonality of precipitation. The narrowness of the Austronesian distribution within the first PCA axis indicates that Austronesian linguistic groups have a similar ecological space position with respect to temperature (Fig 5B) and topography (Fig 5C). Within these trends, Austronesian linguistic groups concentrate around three different environmental settings (Fig 4): one with low precipitation (Fig 5A) occupied by three of the four mainland groups (Mekeo, Roro and Motu, Fig 1), one (Manus Island) with comparatively higher GS values (Fig 5D), and one situated around high temperatures (Fig 5A) and low altitudes (Fig 5C) where the remaining, mainly island Austronesian, groups are situated (Fig 1). The distribution of the TNG linguistic family covers a much larger part of the available NG ecological space (Fig 4) apparently preferring higher altitudes (Fig 5C) and higher GS values (Fig 5D) over those environmental settings where most Austronesian distribution points are found (Fig 4).

### Eco-Linguistic Patterns (ELPs)

Combining ecological dimensions and geographic distributions of the modelled ELNs allows for the identification of ELPs, which offers a means to estimate linguistic diversity within an environmental framework instead of within political boundaries or geodetic grids (related to Earth´s geometric shape). As explained previously, an ELP corresponds to a single ELN if the ELN has a specific ecological space positions and geographic distribution, or to several ELNs when these ELNs present similar ecological space positions and geographical distribution. Contrary to ELNs, ELPs exclude geographical areas that are considered inaccessible to the language group.

Comparisons of ecological space positions and geographic distributions of ELNs highlight: 1) similarities between two pairs of Austronesian groups, i.e. groups 33 and 34, whose ELNs cover two islands (New Britain and New Ireland) of the Bismarck Archipelago and a large part of the northern NG main island, and groups 37 and 38, for which predicted ELNs are located in the South East of NG; 2) that the Eastern and Western Highlands groups (with TNG languages) can be grouped together based on similar geographic and contiguous ecological positions, with the exception of groups 21 and 22, the ELNs of which have a particular geographic distribution and are situated in the center of the PCA; 3) that group 29 can be considered, given its few occurrence points for ELNM, as an outlier; 4) that TNG groups 09 and 25 (both are cases in which the geographic extend of the ELN and the linguistic area largely coincide) can be separated even if they occupy a relatively similar environmental space, as each geographic area of a predicted niche can be considered accessible only if the niche is relatively geographically continuous [28]; and 5) that the geographic distribution distinguish all Austronesian groups from TNG groups with which they overlap according the first axis of the PCA.

Seventeen ELPs can be identified (Table 1, Fig 2): 10 ELPs correspond to ELNs extending the actual linguistic area to which they refer and 7 ELPs correspond to ELNs coinciding with the linguistic area to which they refer. ELP 1 corresponds to language groups 00, 08, 16, 18, 19, 24, 29, for which ELNs depend on temperature range and altitude, and are located in the Highlands. ELP 2 is mainly influenced by precipitation and unites groups 11 and 15 in the center of NG. ELP 3 is composed of groups 12 and 28 in Southern NG and determined by precipitation seasonality and temperature. ELP 4 brings together groups 23, 36, 37, 38 with ELNs determined mainly by precipitation seasonality, located in the Southeastern part of NG. ELP 5 is composed of groups 33 and 34 with overlapping ELNs in the Bismarck Archipelago and across a large part of Northern NG. This ELP is determined by a range of temperatures and a wide range of precipitation/seasonality values. ELNs of groups 20, 21, 22 with PCA values close to the origin, appear to be determined neither by specific environmental variables nor by similar geographic distributions. Although the environmental conditions of group 26 are very similar to those of groups 11 and 15, its distinct geographic distribution classifies the ELN of this group into a distinct ELP. The remaining ELNs, i.e. groups 09, 10, 25, 27, 31, 32, 35 and 39, are all strongly bound to a very confined temperature-dependent environment, which does not display geographic continuities except for the four TNG groups 09 and 10 in Southwestern NG, and 25 and 27 in the Gulf of Papua at the southern coast. However, group 10 in the swampy lowlands of Southwest NG has a very specific geographic position.

### ELPs and language diversity

Counting the linguistic groups included in the same ELP equates to calculating the linguistic diversity on ecological rather than geodetic or administrative criteria. In this way, a linguistic diversity of 1 can be attributed to environments wherein a single linguistic group occupies the full geographic extend of its ELN. ELP 1 comprises seven ELNs meaning that seven linguistic groups share a relatively similar environment, and thus corresponds to a diversity value of 7. In the same way, a diversity value of 2 is found in ELP 2, ELP 3, ELP 5, and a diversity of 4 in ELP 4. The same calculation yields a diversity of 1 to the ELNs of linguistic groups that present limited overlap with others. Notably, only ELP 4 includes both TNG and Austronesian linguistic groups. Also, linguistic diversity in terms of the number of languages per language group(s) included in the same ELP is differs greatly (Table 1).

### ELNs and ecological risk

Application of the ecological risk formula to the study area identifies higher GS values, and hence lower ecological risk, in the Highlands, where the highest linguistic group diversity is observed (Figs 2E and 5D). On the other hand, most language groups for which the ELN geographic distribution and the linguistic area largely overlap, occur in areas with lower GS values (higher ecological risk). No correlation emerges between GS values and number of languages (Table 1).

### ELNs and environmental suitability

**Fig 6:**
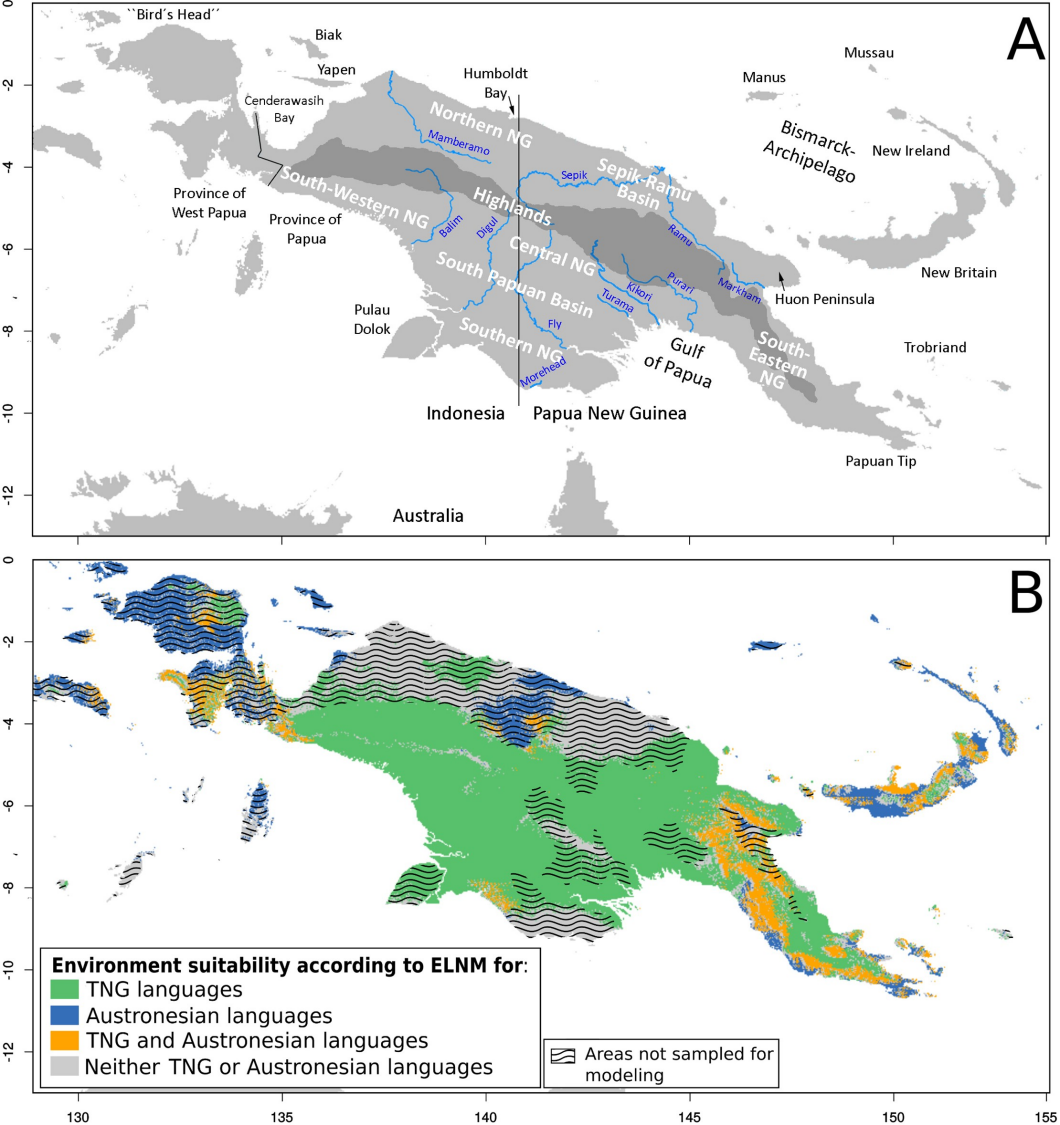
Environment suitability according to Eco-Linguistic Niche (ELN) modeling for Trans New Guinean (TNG) and Austronesian language families. A: Geographic areas in mainland and island New Guinea with relevance to the text. B: Environmental suitability map corresponding to the difference (delta) between the mean predictions of TNG ELNs and Austronesian ELNs calculated with formula ΔELN (cf. Methodology section). Map background generated by using R::Raster CRAN Repository–river shapes obtained from Natural Earth–public domain.

The map superimposing all TNG and Austronesian ELNs (Fig 6) identifies the most suitable areas for each of the two language families, that is, areas with an environment suitable for both families and areas inappropriate for both.

**Fig 7:**
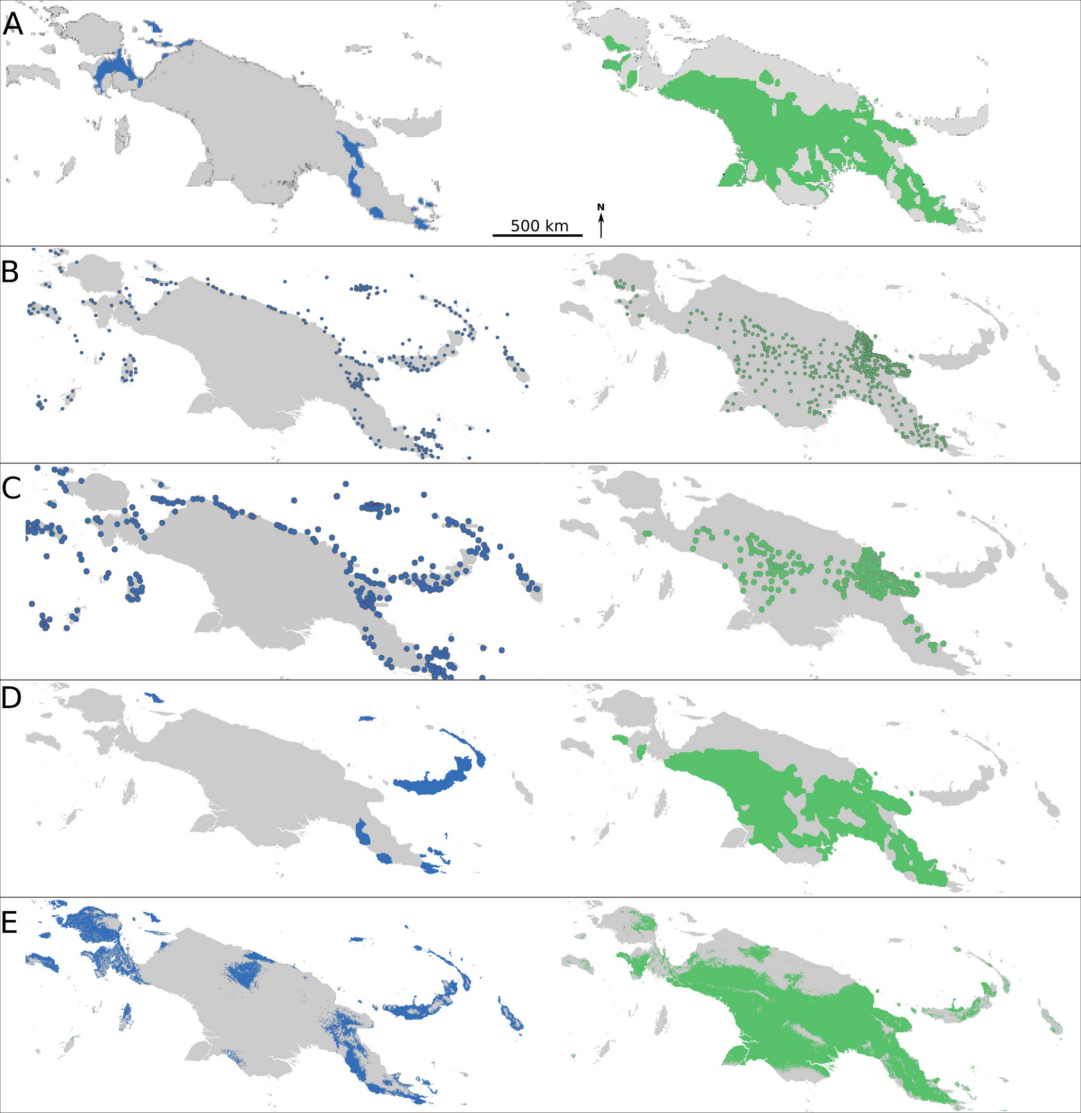
Geographical distributions of language families. Geographical distribution of the Austronesian (blue) and Trans New Guinean language families according to Ross [19] (A), Glottolog versions 2.7 (B) and 3.0 (C) [27], the selected language groups for which Eco-Linguistic Niche modeling (ELNM) was performed (D) and the results of the ELNM (E). Dots in (B) and (C) correspond to the center of the geographical location of the constituent languages of each language family according to Glottolog versions 2.7 (B) and 3.0 (C) [27]. Map A after Ross 2005 [19]; Maps B-E generated by using R::Raster–CRAN Repository–public domain software.

This map predicts much of the geographic distribution of both linguistic families. In the case of the Austronesian language family, there is good correspondence with the Glottolog [27] (Fig 7B and 7C), Muturzikin [30] and Ethnologue maps [31]. In the case of the TNG family, similarity exists with the Ross [19], Ethnologue [31], Muturzikin [30] and Glottolog 2.7 [27] maps (Fig 7). The 3.0 and 3.1 versions of the Glottolog [27] maps correspond less to the ELN prediction for TNG, because their TNG family concept targets the more restrictive Nuclear TNG.

Detailed comparison between predicted ELNs and actual linguistic distribution of Austronesian and TNG language families shows that several areas, not included in the analysis, were predicted to have a potentially favorable environment to accommodate populations speaking a language of the linguistic family identified for these regions, e.g. Yos Sudarso Island (also named Pulau Dolok) at the South Coast of West-NG, where people speak TNG languages, according to Ross [19] (Fig 7A). For “Other Austronesian” languages (41) along the North Coast (e.g. the Austronesian language areas of the Ormu and Tobati speakers west of the Humboldt Bay in the central area of the North Coast, as well as those of the Island of Yapen, south of the Biak Island, and Waropen, a neighboring area on the East Coast of the Cenderawasih Bay, cf. Glottolog 3.0 [27] (Fig 7A). Also, the model correctly indicates that the areas labelled as “Other non-Austronesian NG” languages (30) do not contain groups speaking TNG languages. The “Other non-Austronesian NG” category includes, for example, the languages of the Sepik-Ramu basin and languages of the Ndu group in the central area of the Sepik River Valley both in North-East NG, the languages of the Greater Kwerba family at the mouth of Mamberamo River on the North Coast of West-NG, and Morehead-Wasur and Pahoturi on the South Coast (Fig 6). However, some Austronesian groups inhabit areas not predicted by the model, such as the languages of the Sarmi region on the North Coast of West-NG (Fig 7A).

Taken at the linguistic family level, the Austronesian and TNG ELNs only partly overlap, i.e. only in those regions yielding a null differential of ELN predictions (ΔELN = 0) (Fig 6). This means that some environments are most likely occupied by TNG, others most likely by Austronesians, still others by none or both of these two language families.

## Discussion

### Does environment determine the extent of linguistic areas?

The modelled ELNs define environmentally coherent areas (i.e. environmentally similar and spatially continuous) that are either entirely occupied by a single linguistic group or only partly by one or more linguistic groups. In ecology, the cases where environmentally coherent areas are occupied entirely by one species—corresponding to situations where the realised niche approaches the existing fundamental niche of the species—occur only when there is no significant competition with other species [32]. If ELNs for which the geographic extend of the modeled niche and the linguistic area coincide corresponded to such situations, Asmat, Eleman, Trobriand and Motu (language groups 09, 25, 35 and 38) would each have qualities allowing them to exploit a specific environment, inappropriate to competitors. The nature of these qualities might be biological (e.g. malaria resistance [33]) or cultural (e.g., related to technology [34], warfare, economy, politics, ideology). However, most modelled language groups have realised niches that are smaller than their potential existing fundamental niches. This discrepancy may be explained either by population dynamics (i.e. the language groups might still be in a phase of territorial expansion), competition (i.e. the presence of other language groups prevents access to resources), drift (i.e. the language group were isolated and their languages diverged) or a process of pseudo-speciation, *sensu* Erikson [35]. In sum, cases in which the extent of a linguistic area is predicted by an environmental setting are rare, while many others show a variety of factors, not linked to environment, that appear to preclude a language family from occupying its fundamental ELN. This work represents the first attempt to explore the link between linguistic and ecological variability by applying predictive architectures forged to explore the relationship between biological species and the environment. Future research should focus on measuring the relevance and reproducibility of the results obtained here by testing the informative potential of other predictive tools, including those that do not entail the joint application of several predictive algorithms to reach a consensual prediction.

### Does environment determine linguistic diversity?

No obvious relationship between linguistic diversity and environment is apparent. This is particularly true when linguistic diversity is examined at the level of language groups. Language groups are inconsistently dependent on environment, with some language groups occupying specific niches (ELPs 6–17, Table 1) and others sharing them (ELPs 1–5, Table 1). This is equally the case for language groups within the same language family. Different classifications of the TNG language groups do not contradict this observation: Nuclear TNG language groups, as defined in the Glottolog [27], occupy at least seven different ELPs and of the nine language groups with questionable TNG language family status, three (Bosavi, Angan, Uhunduni) share ELPs with undisputed TNG language groups, three (Eleman, Turama-Kikorian, Kiwai-Porome) occupy specific ELPs, two (Marind, Gogodala) share a proper ELP and one (Southeast Papuan) an ELP with undisputed TNG and Austronesian language groups (Table 1). The number of constituent languages within language groups (Table 1) also lacks correlation with topographic (Figs 1 and 5C) and GS variables (Figs 1 and 5D) suggesting that hypotheses of language diversity being influenced by environmental risk [4] or difficult terrain [16] are not necessarily pertinent. While environmental risk and terrain may not be adequately reflected in the exclusively bioclimatic and topographic variables used in the ELNM, those variables should be diverse enough and of adequate resolution to reliably estimate existing niches, but they do not.

Environment appears to play a more prominent role in the geographic distribution of language families (Fig 7): ELNs and linguistic areas of language families correspond closely. This is enhanced when the ELN predictions for language groups of questionable TNG language family status are excluded, i.e. if only Nuclear TNG [27] languages are taken into account. Large parts of NG (especially North-Western, Northern, Southern and South-Eastern) are environmentally different from the TNG language family’s ELN and, in those areas, languages of other families are spoken [27]. Austronesian ELNs (except for New Ireland and New Britain) also generally do not predict areas where non-Austronesian NG languages are spoken (Fig 6). The ELNM predictions of which geographic areas are suitable for TNG and Austronesian languages, respectively, are relatively accurate, even for areas not sampled in the present study. This finding validates the use of ENM for the analysis of language family distribution and suggests that the geographic distribution of Austronesian and TNG language families is related to environmental conditions. In sum, ELNM allows one to clarify the role of environment in present-day Austronesian and TNG language distributions and, by extrapolation, to infer the possible role of environmental factors in the settlement history of NG.

### Did environment determine the expansion of language families in NG?

The settlement histories of TNG and Austronesian language families still have many blank spots. Nevertheless, a tentative juxtaposition of the obtained ELNM results with the pieces of the puzzle put together by linguists, geneticists and archaeologists can be attempted.

If the TNG language family is correlated with the dispersal of taro-growing horticulturalist populations in the highlands of NG at least 8,000 years BP [36–41], ELNM suggests that they occupied different ELNs during that time span. If the model proposed by Codding and Jones [42] is correct, the settlement of the most suitable niches would have occurred before dispersal into the less optimal niches, and high GS values (Fig 2E) could be taken as a proxy for most favorable niches for taro-growing horticulturalists [4,43], ELPs 1 and 2 appear to include the best ecological niches, but it must be noted that the highest GS values do not correspond to a specific ELP. This suggests that other factors (e.g. soil, earthquake and landslide frequency) also may have played a role. ELPs (3–4, 6–13) with lower GS values then would be occupied only by TNG languages during a second stage. Automated age determination [44] of TNG languages only partly confirms this hypothesis. It suggests that language groups in ELPs 6, 9–11 and 13, which have lower GS values, are indeed younger. However, also younger are the two language groups (11 and 15) in the ecologically well-suited ELP 2, and within ELP 1, which includes the highest GS values, at least one language group (8) has a younger age estimate as well. ELPs 7, 8 and 12, with low GS values, have age estimates as old as those in ELP 1. Homeland identification of the TNG language family based on the diversity principle [45] points to the Eastern Highlands or the Central Highlands where up to four top-level linguistic groups are present. This area however also does not correspond to a specific ELP.

Proto-Austronesian language dispersal took place after the 6,000 year limit imposed by linguistic lineage methods [44], which identifies at least 10 first-order linguistic groups in Austronesian languages today [27,41]. Linguistic evidence [27,37,46–51] suggests for NG that all Austronesian languages East of Cenderawasih Bay belong to the Oceanic Phylum and most of them to the Western Oceanic linkage subgroup [27], with Biak being the only language group of the Eastern Malayo-Polynesian subgroup. The Western Oceanic linkage group, with a possible point of origin in the Bismarck Archipelago, is further subdivided linguistically into the Meso-Melanesian (New Ireland and North of New Britain), the North NG (New Britain and North Coast and Markham Valley of Mainland NG), and the Papuan Tip linkages [27].

Respective ELNs of NG Austronesian language groups distinctly show a gradual expansion in the directions of the ELN edges: Biak ELN shows similar environments on the North coast of NG, on the Island of Mussau, the southern part of New Ireland, the northern part of New Britain and parts of Bougainville and Manus; ELN of New Ireland reaches larger parts of the NG North and West Coast, the Trobriand Islands and the South Eastern Tip of NG, where languages of the Papuan Tip Cluster are spoken; ELN of the Papuan Tip Cluster reaches further into Motu language territories; this expands further towards Roro and Mekeo language territories; ELN of Mekeo includes the far away Markam Valley and Huon Gulf where other Austronesian inland languages are spoken [27]. ELNs of Austronesian language groups thus suggest that most of the present-day language area were reached through gradual geographic expansions. Even if ELN, alone, cannot determine the point of origin, it nevertheless can suggest directions of diffusion and improbabilities such as expansions towards the Bismarck Archipelago from mainland Austronesian language groups, the ELNs of which do not predict New Ireland or New Britain.

Furthermore, the geographic positioning of ELNs allows one to estimate niche overlaps between language groups and identify regions where competition within niches may have occurred. Regions where there is overlap between TNG and Austronesian ELNs (Fig 6) could reflect the most coveted areas where territorial conflicts (over environmental resources) could have existed or areas where language shifts may have led to expansions.

The competitive exclusion principle stipulates that two species cannot coexist indefinitely within the same ecological niche [32,52,53]. However, competitive exclusion may be avoided by niche shifting, in which one or both specie(s) migrate(s) or time-shift(s) its resource exploitation [54]. This principle can also be applied to human groups living in the same territory and competing for resources.

Recently, d’Errico and colleagues [55] proposed that human adaptive systems allow a population to shift its niche and exploit new environmental conditions. We suggest that, similarly, populations sharing a language, seeking to avoid competition with other language groups in the same niche, may have developed cultural adaptions to enable them to exploit environmental components that previously were used only rarely or intermittently. The present small overlap between the two NG linguistic families (TNG and Austronesian) indicates that only a few of their subgroups compete for resources that are ecologically relevant to others. The existence of some intermingled Austronesian/TNG ELPs (Table 1) may reflect past competition, language shift, adoption of new behaviours, convergence of adaptive systems, or niche shifts to avoid competition. For the Markham Valley, Ballard [56] reported that the Austronesian speaking Mari were pushed up the Markham River by warfare with the Austronesian speaking Adzera. The Mari kept their pottery tradition (a typical element of Austronesian cultures) but adopted the language of their TNG-speaking Gadsup neighbors.

Minor differences between language family ELN predictions and reality exist: For example, Austronesian language is predicted (mainly by the ELN of New Ireland), but not observed, on the South coast of the North-Western NG peninsula (i.e. the “Bird’s Head”) and the North-Central NG inland. TNG language is observed, according to Ross [19], but not predicted on the South coast of the “Bird’s Head” (Figs 6 and 7). These discrepancies might be explained by: 1) inaccuracy of the ELNM methodology; 2) historical reasons; or 3) missing linguistic data. ELNM accuracy certainly would benefit from the inclusion of additional environmental parameters (e.g. soil and vegetation), in-depth checking of the linguistic affiliations in the areas where multiple language groups are present (e.g. New Britain and New Ireland [57]), or apparent (e.g. differences between Ross [19] and Glottolog 3.0 [27] for the South East Papuan language group linguistic area). Further genetic, linguistic and archaeological studies should help to clarify (pre)historical events in the areas concerned.

**Fig 8:**
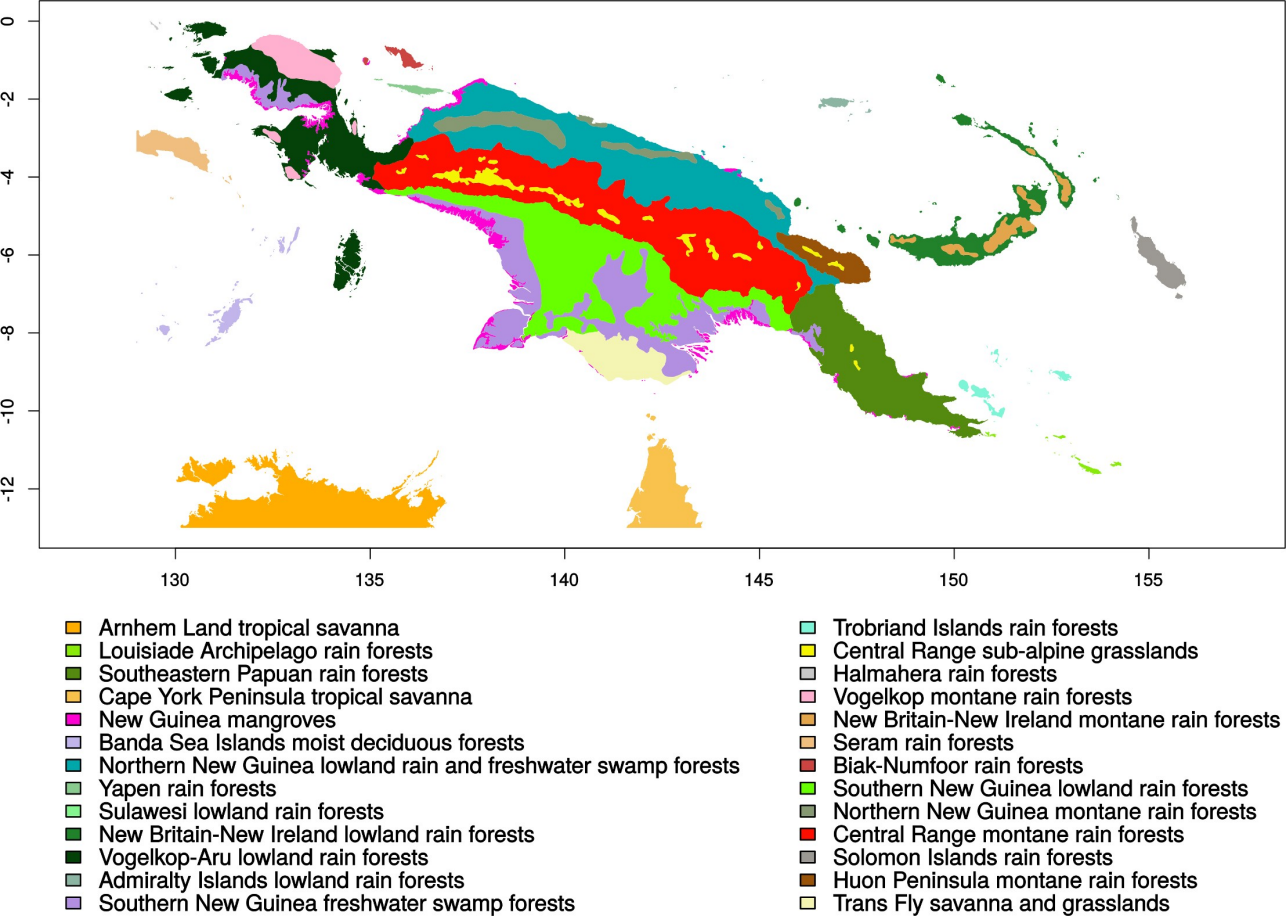
Ecosystems of New-Guinea according to the World Wide Fund For Nature (WWF) ecosystem database [58]. Map generated by using R::rgdal package—CRAN Repository–public domain software.

ELNM allows one to pinpoint by multi-variate analyses the ecological preferences of Austronesian language families (Figs 4 and 5). Results show that low altitude, flat terrain, high precipitation (3000–4500mm/year), and high mean annual temperature (27–29^°^C) drive those preferences. However, it is notable that ELN predictions (although mainly from New Ireland, S1 Fig) for the Austronesian language family (Figs 6 and 7) also include the *a priori* non-preferred swampy forest environment on the South coast of the “Bird’s Head” (Fig 8), which ELNM is unable to detect. This discrepancy suggests that ELNM would benefit from inclusion of additional environmental variables.

Modelling of ELNs at different phylo-linguistic levels, identification of correlated environmental variables, and calculations of language diversity within ELPs provide a new approach with which to correlate linguistic area and language diversity with environment and to test cultural expansions by taking into account ecology. Our approach, via consensus-building methods, presents a way to overcome the ENM problem of seeking the most appropriate single algorithm that provides optimal predictions in all circumstance [59]. Instead, it enables identification of robust relationships between linguistic groups and environment variables. Also, identification of ELPs based on geographic space and ecological positions of the modelled ELNs provides a new means of estimating linguistic diversity while avoiding the biases of earlier research induced by the use of political borders or geodetic grids.

## Conclusion

Our results suggest that while the expansion of TNG and Austronesian language families, and the current linguistic diversity of NG, have been and are influenced by environmental constraints, rather than acting as prime movers, such influences act in concert with many other factors, such as evolved socio-dynamics, ethnocentricity, and warfare. Only by understanding the mechanisms that have allowed environmental and non-environmental factors to interact will we achieve a better understanding of the processes that influenced current linguistic diversity.

## Materials and methods

In this study, an Eco-Linguistic Niche (ELN) represents the range of environmental conditions present in the territory of a population speaking a specific language or group of languages characterised by common language traits. In order to reconstruct ELNs of New Guinea (NG), we used language groups belonging to two major NG monophyletic language families (Trans New Guinea (TNG) and Austronesian), transformed their geographical distribution into occurrence data, assembled available environmental data for NG, and employed predictive architectures developed in the field of ecology.

### Predictive algorithms

To reconstruct the ELNs of the selected NG language groups, we used 10 predictive algorithms developed by ecologists to reconstruct species ecological niches and geographic distributions [24] and available in OpenModeler [60] (Table 3 in S1 File). We employed 15 different settings to estimate niches and then used a consensus method [61] to calculate the best-fit model from the 15 predictions (Table 4 in S1 File).

### Linguistic data occurrences

Language groups from two purportedly monophyletic language clades were considered: the TNG and the Austronesian language families (Fig 7). The linguistic data (geographical distributions and labelling) of the language groups selected for ELNM (Fig 1) were taken from maps and databases provided by Ross [19], the Ethnologue (15th ed.) [31], Schiefenhövel [29], Muturzikin [30], and the Glottolog versions 2.7 and 3.0 [27], World Atlas of Language Structures (WALS) [62] and Newguineaworld [63]. The number of languages per linguistic group and its phylo-linguistic rank (Table 5 in S1 File) were taken from the Glottolog 3.0 database [27].

Index numbers for the language groups correspond to those in Schiefenhövel [29] except for group 00, which corresponds to groups 1–7 (i.e. West Bomberai, Ekari, Moni, Demal, Woni, Dani, and Yali) in Schiefenhövel [29], and to the West Trans New-Guinea (WTNG) subgroup of Ross’ classification [19]. Bringing together these seven groups yielded a division of the TNG family with linguistic subgroups at a similar phylo-linguistic top-level similar to Ross [19] (Table 5 in S1 File). Ideally, the chosen linguistic groups constitute linguistic “genera” wherein speakers can largely understand each other’s speech. It is however clear that specialists of NG languages [19,25,64–66] and available online language databases such as Glottolog [27], Muturzikin [30], Ethnologue [31], WALS [62] and Newguineaworld [63], providing “genetic” groupings of languages, have different bases for classification and do not necessarily agree with each other; they must be considered as provisional and subject to ongoing refinement. The TNG status of nine (Marind, Bosavi, Southeast Papuan, Angan, Eleman, Turama-Kikorian, Kiwai-Porome, Gogodala-Suki, Uhunduni) of the 20 modelled language groups (Table 5 in S1 File) is for example debated. The inclusion of the Kiwai-Porome Group to the TNG language family is questioned by Ross [19], and abandoned in the Glottolog 3.0 [27], which attributes Kiwaian to an independent non-Austronesian language family and Porome to a dialect of the Kibiri language, which could be the only representative of another non-Austronesian language family. The Southeast Papuan language group is composed of languages (Koiarian, Kwalean, Manubaran, Yareban, Mailuan, Dagan, Gailalan) that may not be linked to TNG and to each other according to Glottolog 3.0 [27]. Also, according to Glottolog 3.0 [27], Marind would be a subgroup of the Anim language family, independent of the TNG language family. Bosavi, Gogodala-Suki, Turama-Kikorian, Eleman, and Angan would also each correspond to a separate non-Austronesian language family (Glottolog 3.0 [27]).

The linguistic groups “Other non-Austronesian NG” (30) and “Other Austronesian” (41) were not included, as they do not constitute phylo-linguistically homogeneous linguistic entities according to Ross [19] and Glottolog [27]. Given their remoteness from NG, the linguistic groups of Timor and Alor islands (13, 14, 40) were excluded from the present analysis. This geographical restriction was preferred to enhance modelling accuracy (see “geographical extent” section).

Polygons were generated for the 29 selected linguistic groups by using the GNU Image Manipulation Program software 2.10 [67]. Using R software [68] and the Quantum-GIS geographical information system [69] each polygon was georeferenced. Using the DIVA-GIS database [70], we selected 1326 localities (populated places–i.e. villages) as occurrence points for the ELN modelling. These localities were then classified according to the defined language polygons. Within each language polygon, up to 50 occurrence points (Fig 1, Table 6 in S1 File) were randomly sampled in order to estimate an ELN. This method allowed reliable predictions for the modelling of each language group, an exception being the Uhunduni. The minimum required for the modelling algorithms is 10–20 occurrences [71]. Finally, as the geographical boundary between the Ok and the Awyu-Dumut (Fig 1) follows the international boundary between Indonesia and Papua New Guinea, this boundary was considered artificial.

In order to compensate for the presence of unrepresentative occurrences generated by sampling or assignment error, or particular historical events, we applied an omission threshold with a sensitivity of 0.10 to the ELN predictions, according to the fixed sensitivity method *sensu* Pearson & Dawson 2004 [72], and Peterson 2011 [73]. The probabilities of presence below this threshold in ELNM predictions were reduced artificially to zero in order to limit the influence of any minority aberrant occurrences to calculate a niche.

### Geographical extent

In ecological niche reconstructions, it is recommended to set geographical limits of the study area in which the predictive architecture is allowed to extrapolate (M) to be consistent with the known ecology and dispersal capacity of the studied species [28]. Due to their maritime knowledge, Austronesian populations have a high dispersal capacity, so we did not seek to define the extent of M based on their distribution. Instead, we used the TNG speakers’ dispersion to delimit M (Fig 1). Places inhabited by TNG speakers are essentially present on the main island of NG, whose environment is dominated by moist broad-leaf forest (Fig 8). Timor and Alor islands were excluded from the M because of their remoteness from NG and their differing vegetation (dry broad-leaf forest). With respect to latitude and longitude, the M for the ELNM of TNG and Austronesian language distributions falls between the equator and the 11^°^ South parallel, and between 130^°^ East and 153^°^ East meridian.

### Environmental data

High-resolution ELNM requires environmental predictors that consist of gridded data layers containing numerical values for a given variable at each grid point. Predictors need to be complete and to have the same extent and resolution within the studied area. We used 26 environmental variables that summarise climate and topographic data (Table 2).

**Table 2:** Environmental variables used for Eco-Linguistic Niche Modeling. Climatic variables obtained from the WorldClim model [74]; topographic variables are derived from the altimetric model ETOPO1 [75].

code	Description
bio1	Annual Mean Temperature
bio2	Mean Diurnal Range Temperature
bio3	Isothermality
bio4	Temperature Seasonality
bio5	Max Temperature of Warmest Month
bio6	Min Temperature of Coldest Month
bio7	Temperature Annual Range
bio8	Mean Temperature of Wettest Quarter
bio9	Mean Temperature of Driest Quarter
bio10	Mean Temperature of Warmest Quarter
bio11	Mean Temperature of Coldest Quarter
bio12	Annual Precipitation
bio13	Precipitation of Wettest Month
bio14	Precipitation of Driest Month
bio15	Precipitation Seasonality
bio16	Precipitation of Wettest Quarter
bio17	Precipitation of Driest Quarter
bio18	Precipitation of Warmest Quarter
bio19	Precipitation of Coldest Quarter
Aspect	Azimuth that slopes are facing
DEM	Altitude in meters
Flowdir	Flow Direction of Water
Slope	Slope in degrees
TPI	Topographic Position Index
TRI	Terrain Ruggedness Index
Roughness	Eight surrounding Cells Altitude Difference

Selecting and using non-correlated environmental variables improves niche estimations by reducing overfitting and is common practice. However, we retain all available bioclimatic variables (S2 File) despite them being highly correlated in average because not all regions are characterised by the same sets of correlated variables. Furthermore, we minimise overfitting due by employing an "ensemble" approach, as well as an omission threshold (see previous section on linguistic data occurrences).

### Climate data

Climatic data come from the WorldClim 1 model [74] and comprise 19 bioclimatic variables (Table 2: bio1–bio19). The WorldClim 1 database provides a monthly interpolation of mean temperatures and precipitation collected between 1950 and 2000. The algorithm ANUSPLIN that generates this interpolation uses latitude, longitude, and elevation as independent variables [74]. Mean values of the primary environmental data used can be found on the World Clim database website [76]. Using these climate data allows for the integration of seasonal variability into the analysis. The fine-scale WorldClim grid allows modelling at a resolution of 30 arc-second (ca. 1 km^2^). It should be noted, however, that weather stations used by WorldClim shows a substantial difference in coverage between Papua New-Guinea and Indonesian NG. This means that WorldClim environmental coverages would lean more heavily on spatial interpolation of environments on the Eastern side instead of the Western side of the island. Although the use of multiple climatic datasets has been recommended [77], we used exclusively the WorldClim simulation. To avoid potential biases, we increased predictive reliability by combining multiple algorithms and multiplying statistical evaluations of niche predictions (cf. supra).

### Topographic data

Seven topographic variables (Table 2: Aspect, DEM, Flowdir, Slope, TPI, TRI, Roughness) were calculated from elevation data via ETOPO1 [75]. Variables were obtained using the R package “raster” [78,79]. This dataset includes: mean altitude, slope, slope orientation, roughness (or relief), flow direction, Topographic Position Index [80], and Terrain Ruggedness Index [81]. These variables also have a 30 arc-second resolution.

### Prediction reliability improvement

Different types of predictive architectures exist, each relying on particular methods (e.g. machine learning, artificial intelligence, multi-factorial analyses, statistical regression, hierarchic classification, etc.), thus producing variability in their respective predictions. No single algorithm is able to model the most reliable niches, regardless of the dataset [59,77], even if some predictive algorithms, such as DesktopGARP and MaxEnt, are used more often, due to their reliability or ease of use [77]. The accuracy of a prediction depends on the dataset, modelled populations, and employed architecture [59].

To address these issues, some authors [82,83] suggest using several algorithms and comparing the various niche estimations, or building consensus models that summarise various estimations obtained with different methods. We applied 15 predictive architectures (Table 3 in S1 File). Ten were set with default parameters in OpenModeler [60] and four were configured with customized settings that impose fewer restrictions on the predictions (those are labelled “light”, Table 3 in S1 File).

In order to improve clarity and not favour a particular a predictive architecture to the detriment of others, we present results in the form of weighted elitist consensus reconstructions generated by a three-step process: Selection of the best predictions, weighting by prediction relevance, and addition of selected and weighted predictions (Table 4 in S1 File).

### Statistical analysis and characterisation of Eco-Linguistic Niches (ELNs)

An ecological niche can be conceived as a “hypervolume” in which each dimension represents an environmental factor [32]. Accordingly, the factorial parts of a multivariate analysis can be considered as a visual representation of this hypervolume. We generated a principal component analysis (PCA), in which variables correspond to environmental parameters and individual data-points to each grid-point of the studied area. This visualisation gives the total available environment in the geographic area under study and the environment present within the territory of the different linguistic groups. In such a graphic representation, position and size of the ellipses (summarizing 61% of the individuals’ scatter distribution) depict: 1) contribution of each environmental variable to the niche; 2) amplitude of the environmental variations for each linguistic group; and 3) position of the niches in environmental “hyperspace”. Ellipse size is proportional to the amplitude of the environmental conditions within the niche, i.e. it corresponds to the “ecological breadth” of the niches [84] and is inversely correlated to its degree of specialisation [85]. Superposition of the ellipses is indicative of overlapping environments between niches. Thus, we could determine whether or not the geographical distribution of a population is subject to environmental constraints and identify the most influential environmental parameters. This representation also allows for assessment of the remoteness or affinity of the modelled ELNs.

We generated a unique PCA, grouping the predictions of the 29 ELNs to determine their respective positions within the total available ecological space represented by the factorial plans of the PCA. This PCA was computed from the values of all environmental variables within the geographical extent of NG, plus the ELN predictive values having a probability greater than 0.9. Doing so allows better evaluations of variation between the ELNs and their position in the available environmental space. This applies especially as the total environmental space is displayed in the background wherein particular environmental subspaces (e.g. altitudinal range, temperature- and precipitation range, or any combination of environmental variables) can be highlighted with colour scales. Thresholding of predictive values helps to reduce the background noise generated by the consensus method.

As proposed by Warren et al. [86], niche-similarity metrics Schöner’s D and Hellinger I distance were used to measure quantitatively the degree of niche overlap. These overlap metrics range from 0 to 1, in which 0 indicates a perfect disjunction (i.e. no overlap) and 1 complete overlap (i.e. identical niches). We computed a pair-wise overlap comparison of the 29 ELNs, using the R package Phyloclim [87]. We calculated a matrix of dissimilarities from the overlap matrix in which dissimilarity corresponds to “1-overlap value”. Finally, we generated a hierarchical clustering from this matrix, employing the agglomerative complete-linkage method and Euclidean distances.

The different ecosystems covered by the 29 ELNs were labelled as Eco-linguistic Patterns (ELPs) and identified based on high geographical overlap, similar position in the PCA, and high paired overlap scores of the ELNs. We propose that the number of groups within each of these defined ELPs offers a useful way to estimate linguistic diversity, according to ecological criteria.

### Eco-linguistic potential calculation

In order to identify regions with environments potentially favourable to the presence of TNG and Austronesian language families, we averaged predictions for each of these two families. The geographical projection of such an average calculation allows the calculation of a value for each geographical location. This value corresponds to the number of constituent linguistic groups predicted as having suitable environmental conditions at this location. This score was named *eco-linguistic potential*. The difference between TNG and Austronesian eco-linguistic potentials was calculated according to formula 1:

$$\Delta_{\mathrm{ELN}}=\frac{\sum_{i=0}^{\mathrm{29}}{\mathrm{ELN}}_{i}}{n_{\mathrm{TNG}}}-\frac{\sum_{j=\mathrm{31}}^{\mathrm{39}}{\mathrm{ELN}}_{j}}{n_{\mathrm{Austronesian}}}$$ (1)

In which *i* and *j* correspond to the index number of language groups, nTNG to the total number of TNG language groups (i.e., 20), and nAustronesian to the total number of Austronesian language groups (i.e., 9). This difference was projected geographically to identify differences in the suitability of the environment for the two language families. In order to identify the environmental parameters prevailing in regions suitable for TNG, Austronesian, or both language families, we projected the eco-linguistic potential scores onto the PCA of the available environmental space.

### Ecological risk calculation

*Ecological risk* is defined as the reciprocal of the Mean Growing Season (MGS), in which the MGS is the number of months during which vegetation can grow [4]. This calculation used Le Houérou’s formula [43] as: “A month is included in the growing season if the average daily temperature is more than 6^°^C and the total precipitation in millimeters is more than twice the average temperature in centigrade.” [4].

In Nettle’s study [4], the MGS of Papua New-Guinea (eastern half of NG) was based on data from meteorological stations and estimated to last 10.88 ± 1.96 months. The MGS of Indonesia (western part of NG plus all Islands from the Moluccas to Sumatra) was estimated to be 10.67 ± 1.82 months. This calculation is questionable, because the territory of a geographically diverse country such as PNG encompasses varied eco-types that could yield erroneous MGS calculations (Fig 8). In order to test Nettle’s hypothesis, Le-Houérou’s Growing Season (GS) formula was modified and applied, using WordClim’s monthly temperature and precipitation variables according to the formula 2:

$$\mathrm{IF}\left[(\frac{\mathrm{Precipitation}}{\mathrm{Temperature}}>2)\mathrm{AND}(\mathrm{Temperature>}6\mathrm{{}^{\circ}C})\right]\mathrm{THEN}\;\mathrm{Month}\;\mathrm{is}\;\mathrm{included}\;\mathrm{in}\;\;\mathrm{the}\;\mathrm{GS}$$ (2)

Thus, a high-resolution (one value per km^2^), gridded GIS layer was created for GS values, which provided a more precise determination of the ecological risk.

## Supporting information

S1 Fig Eco-Linguistic Niches (ELNs) of New Guinea language groups. Colour shades reflect probability of niche presence. Green colour is used for Trans New Guinean ELNs and blue for Austronesian ELNs. The purple lines delimit linguistic areas.
(PDF)

S1 File Supplementary texts 1–2 and tables 1–6 Text 1. Eco-Linguistic Niche Modeling (ELNM); Text 2. Papuan and Austronesian settlement, language diversity and phylogeny in New Guinea (NG); Table 1. Characterisation of the Eco-Linguistic Niche (ELN) of each language group; Table 2. Matrices of overlap values between Eco-linguistic niches of language groups; Table 3. Algorithms from OpenModeler with their parameter settings used for the prediction of Eco-Linguistic Niches of language groups; Table 4. Description of the consensus building method used for Eco-Linguistic Niches Modeling and obtained values for Accuracy (ACC), Area Under the “Receiver Operating Characteristics (ROC)” Curve (AUC) and Partial ROC ratio (P-ROC-ratio); Table 5. Phylolinguistic classification of Papuan Trans New Guinean language groups; Table 6. Geographic coordinates of the selected villages.
(PDF)

S2 File ELNM–Variable correlations: Should correlated environmental variables be excluded from the ELN estimation process for New Guinea (NG)? (PDF)
