# Supplementary material for: Quantitative methods demonstrate that environment alone is an insufficient predictor of present-day language distributions in New Guinea
Source: PLoS One. 2020 Oct 7;15(10):e0239359. doi: 10.1371/journal.pone.0239359 (PMC7540881; doi:10.1371/journal.pone.0239359)
Supplement: S1 Fig — Colour shades reflect probability of niche presence. Green colour is used for Trans New Guinean ELNs and blue for Austronesian ELNs. The purple lines delimit linguistic areas. (PDF) [file pone.0239359.s001.pdf]

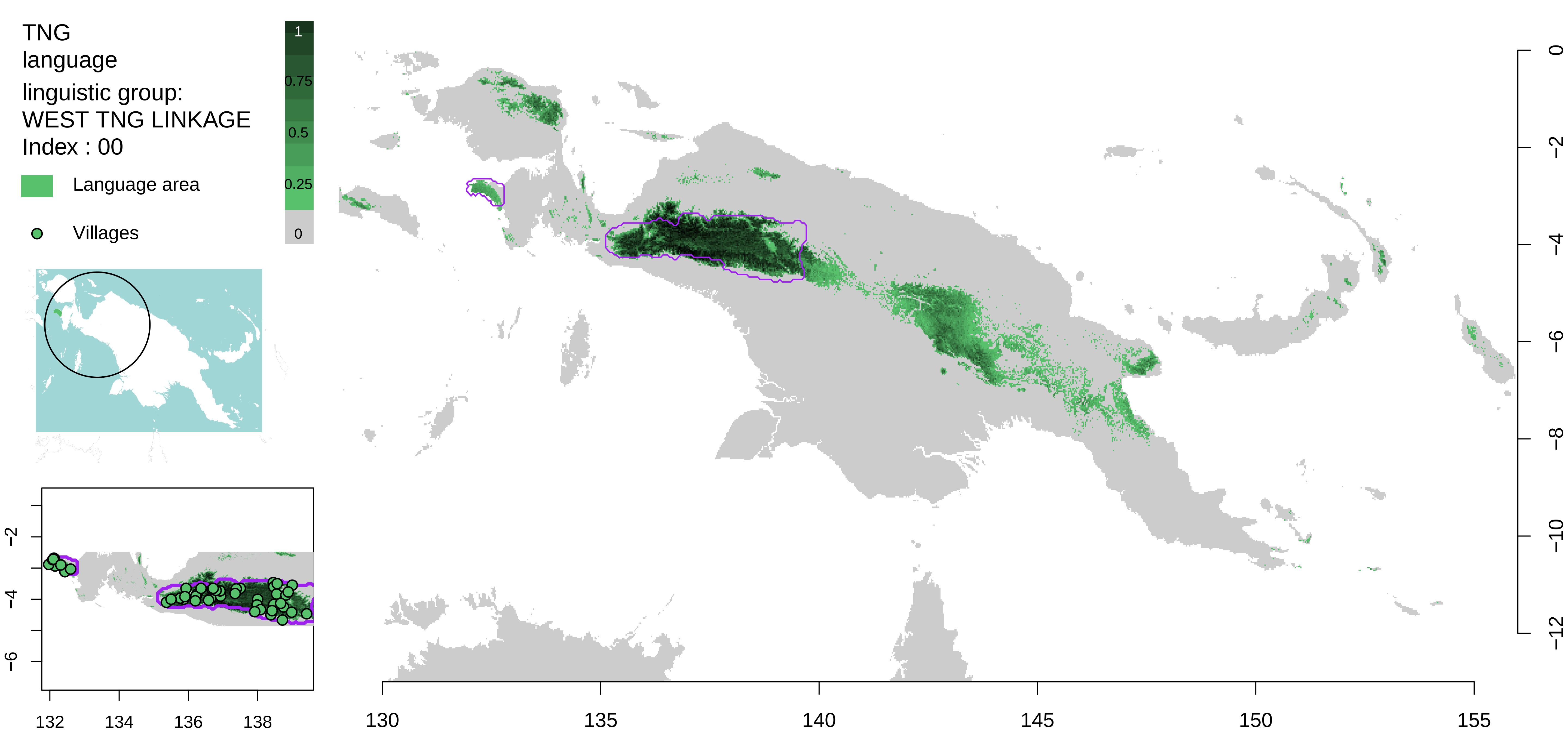
TNG
language
linguistic group:
WEST TNG LINKAGE
Index : 00
Language area
Villages
1
0.75
0.5
0.25
0
130
135
140
145
150
155
0
−2
−4
−6
−8
−10
−12
132
134
136
138
−2
−4
−6

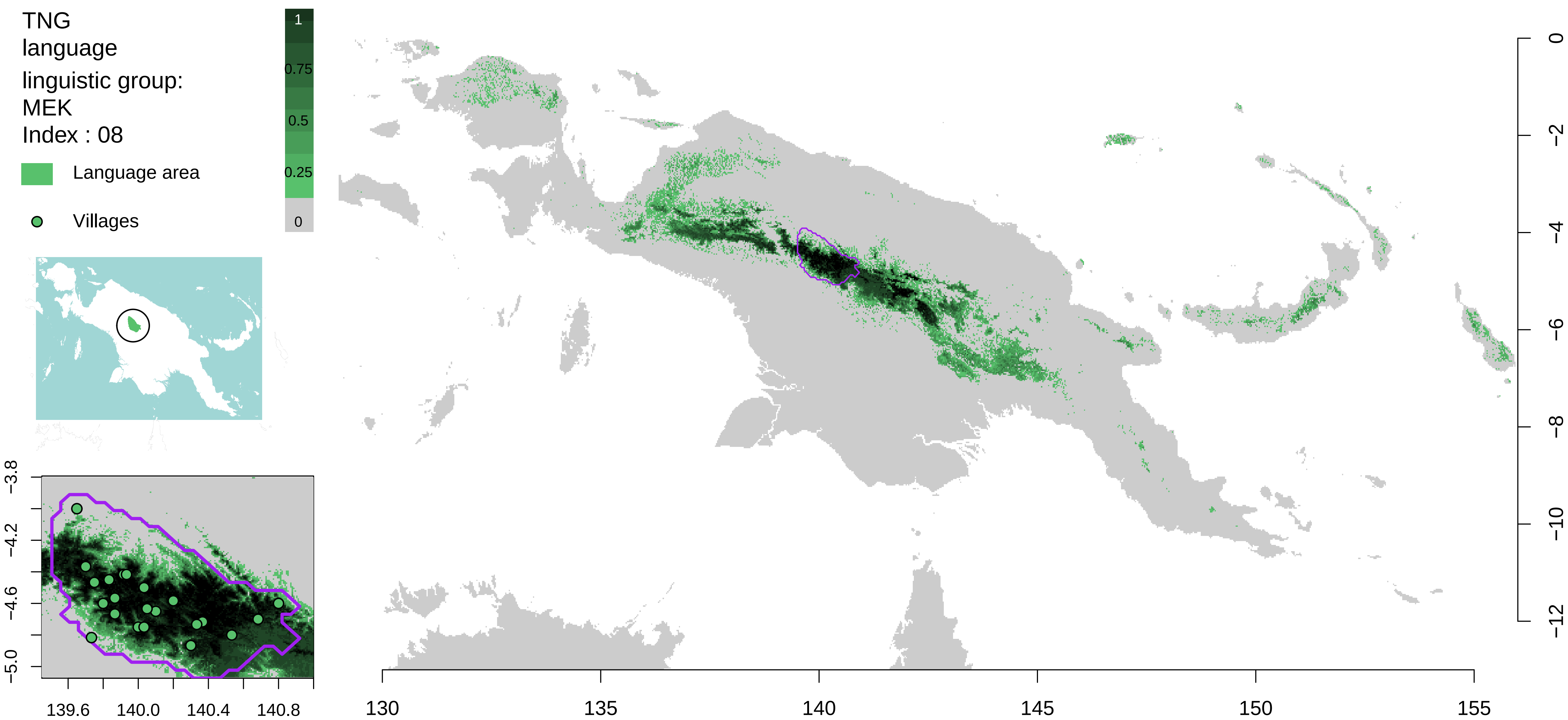

TNG
language
linguistic group:
MEK
Index : 08
Language area
Villages
1
0.75
0.5
0.25
0
−3.8
−4.2
−4.6
−5.0
139.6
140.0
140.4
140.8
130
135
140
145
150
155
0
−2
−4
−6
−8
−10
−12

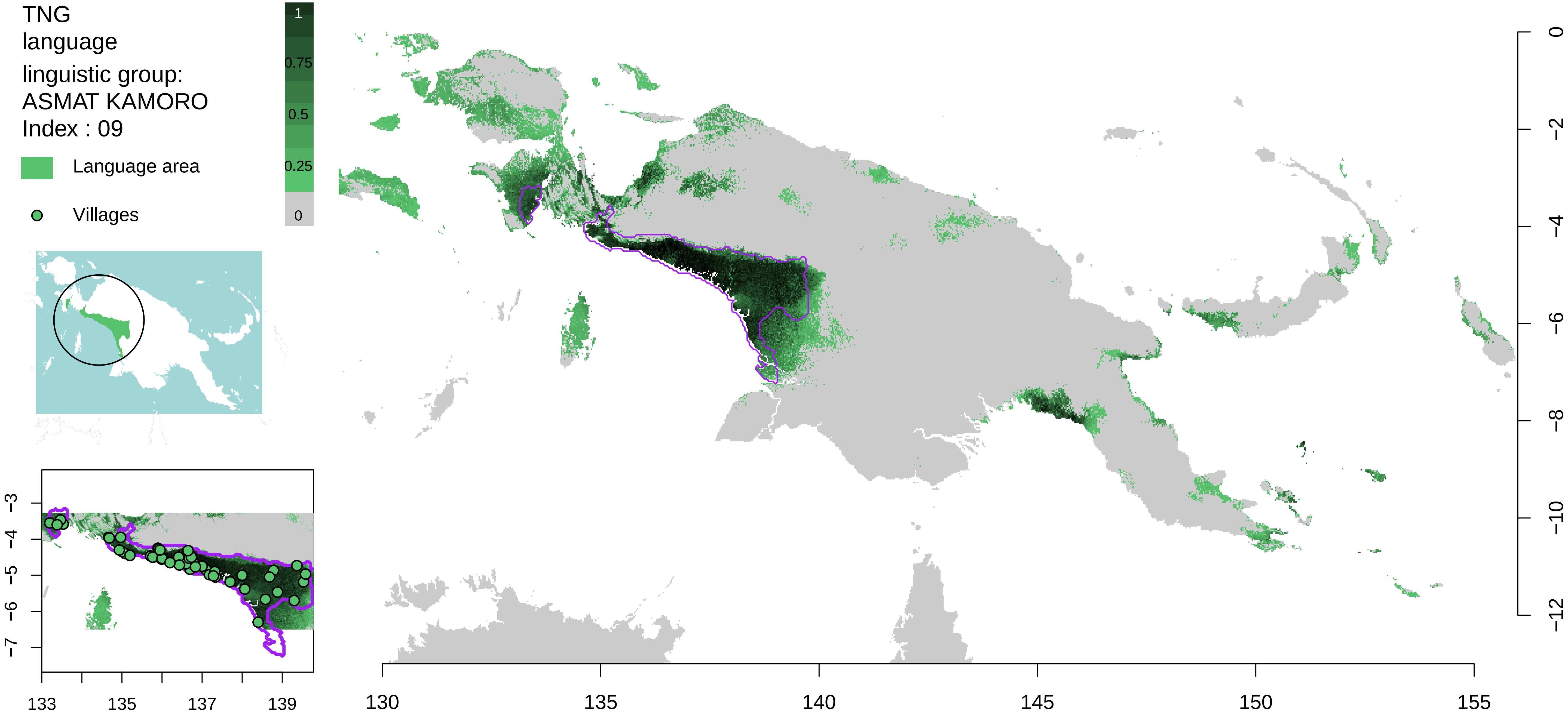

TNG
language
linguistic group:
ASMAT KAMORO
Index : 09
Language area
Villages
1
0.75
0.5
0.25
0
0
−2
−4
−6
−8
−10
−12
130
135
140
145
150
155
−3
−4
−5
−6
−7
133
135
137
139

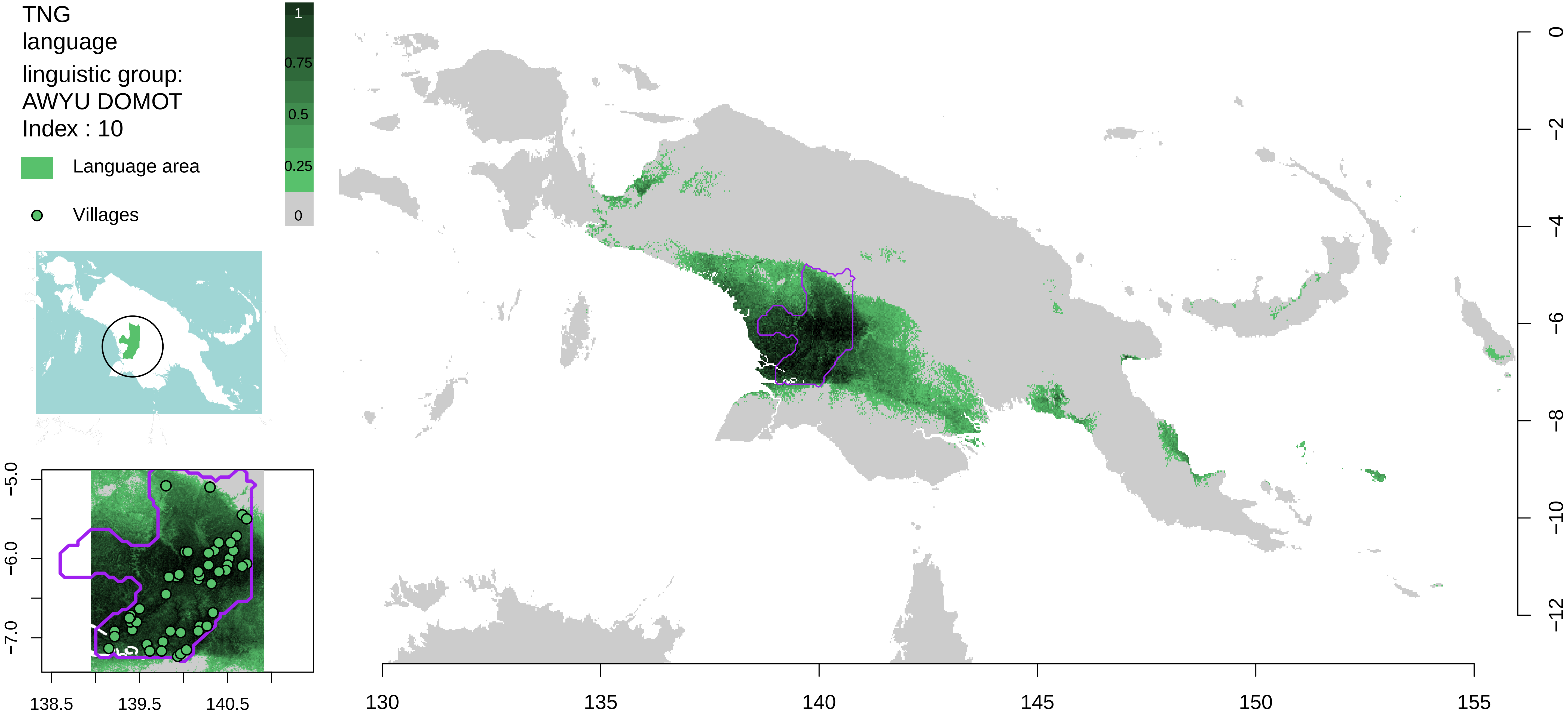

TNG
language
linguistic group:
AWYU DOMOT
Index : 10
Language area
Villages
1
0.75
0.5
0.25
0
−5.0
−6.0
−7.0
138.5
139.5
140.5
130
135
140
145
150
155
0
−2
−4
−6
−8
−10
−12

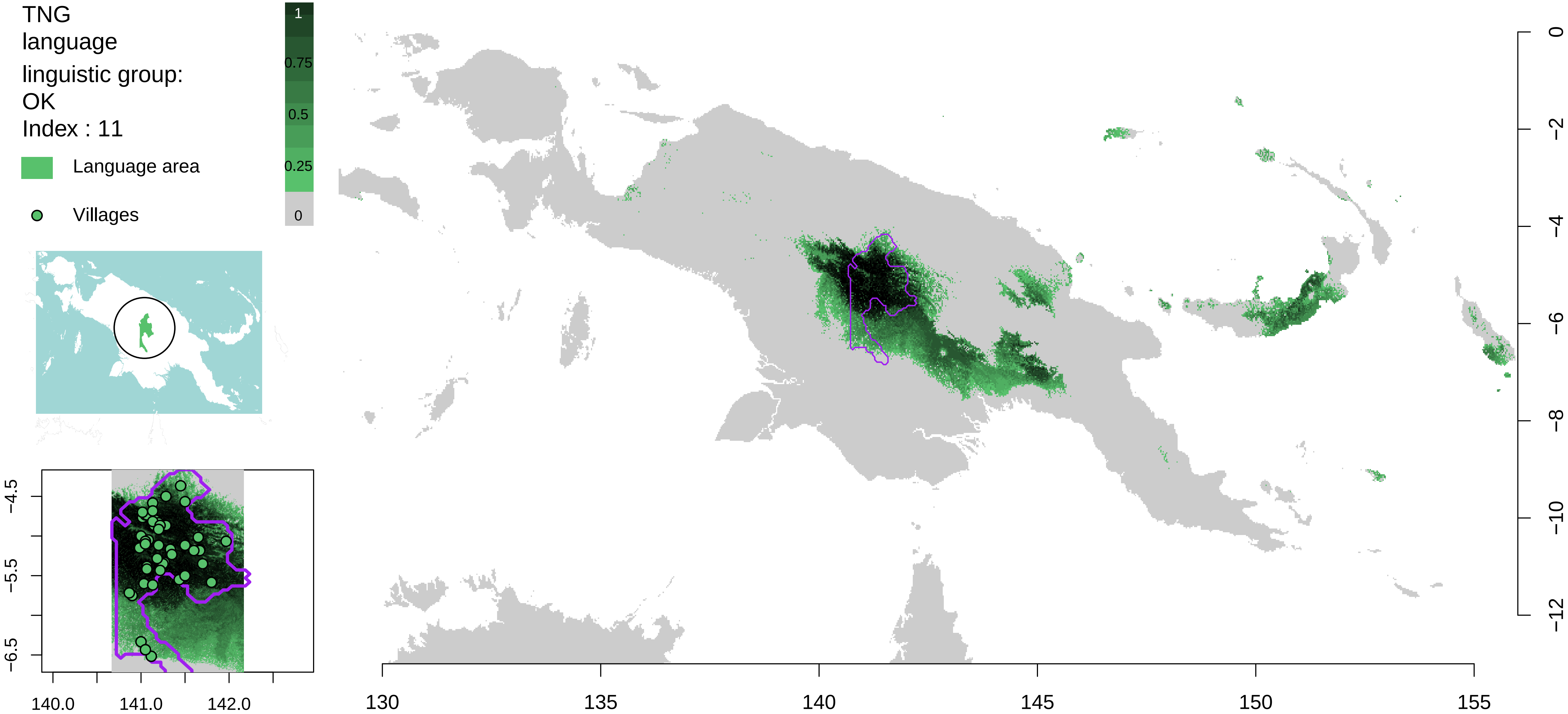
TNG
language
linguistic group:
OK
Index : 11
Language area
Villages
1
0.75
0.5
0.25
0
−4.5
−5.5
−6.5
140.0
141.0
142.0
130
135
140
145
150
155
0
−2
−4
−6
−8
−10
−12

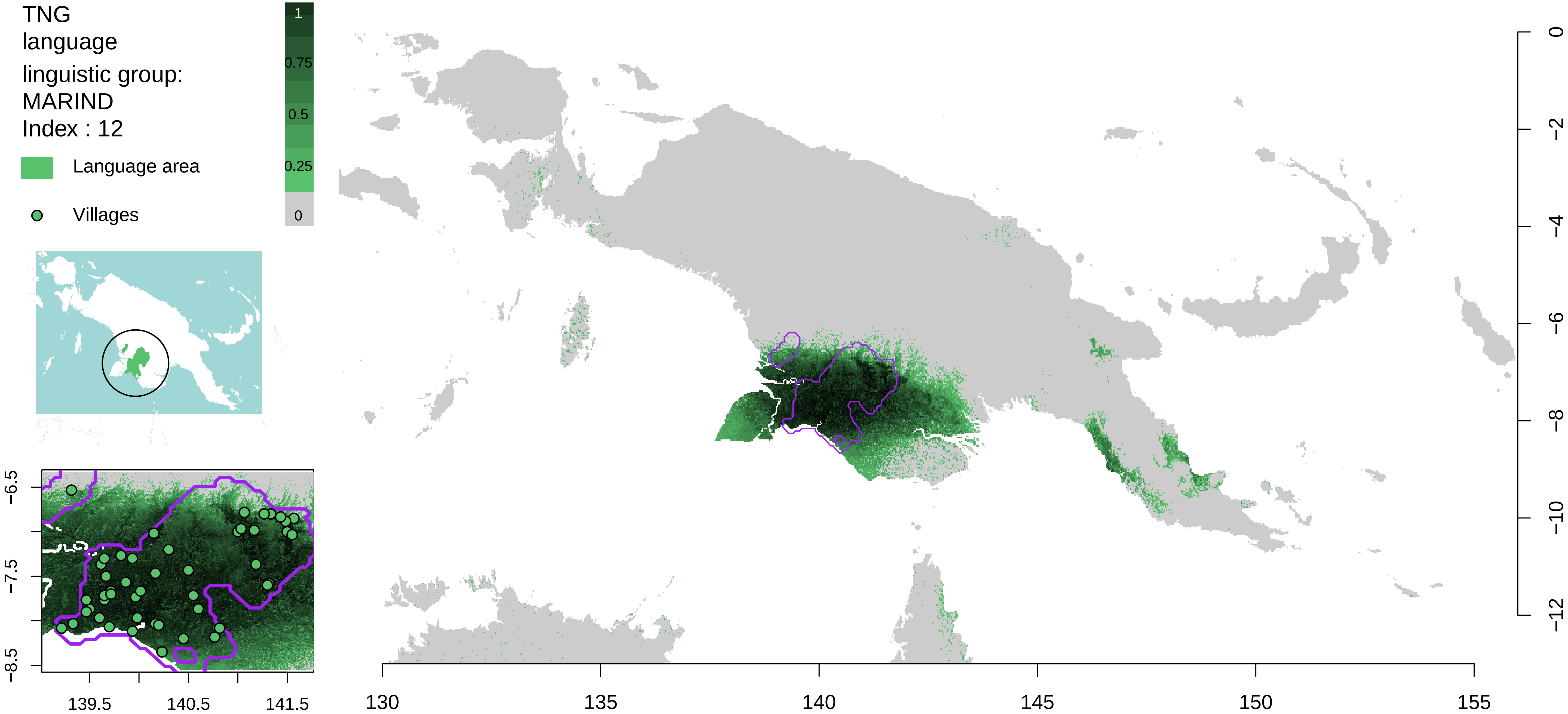
TNG
language
linguistic group:
MARIND
Index : 12
Language area
Villages
1
0.75
0.5
0.25
0
−6.5
−7.5
−8.5
139.5
140.5
141.5
130
135
140
145
150
155
0
−2
−4
−6
−8
−10
−12

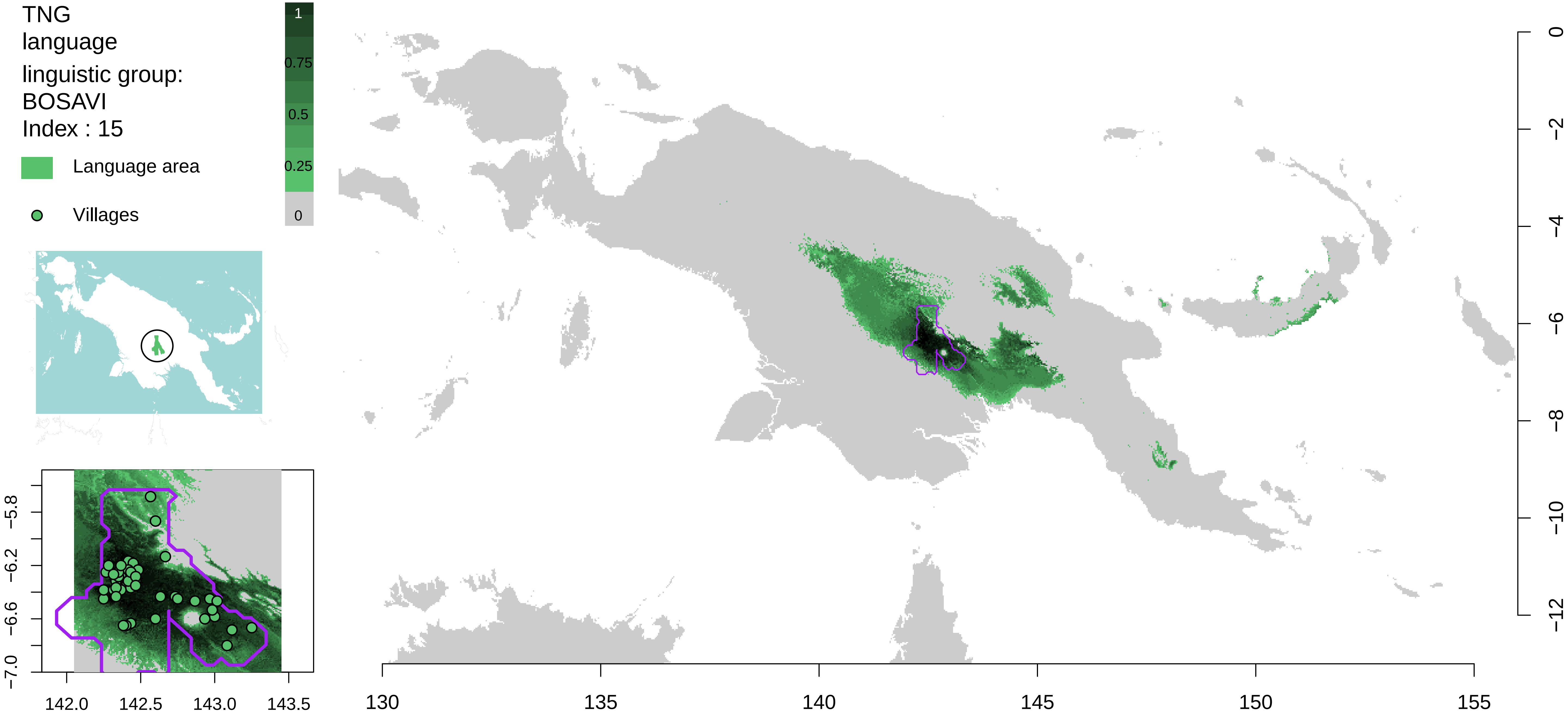

TNG
language
linguistic group:
BOSAVI
Index : 15
Language area
Villages
1
0.75
0.5
0.25
0
−5.8
−6.2
−6.6
−7.0
142.0
142.5
143.0
143.5
130
135
140
145
150
155
0
−2
−4
−6
−8
−10
−12

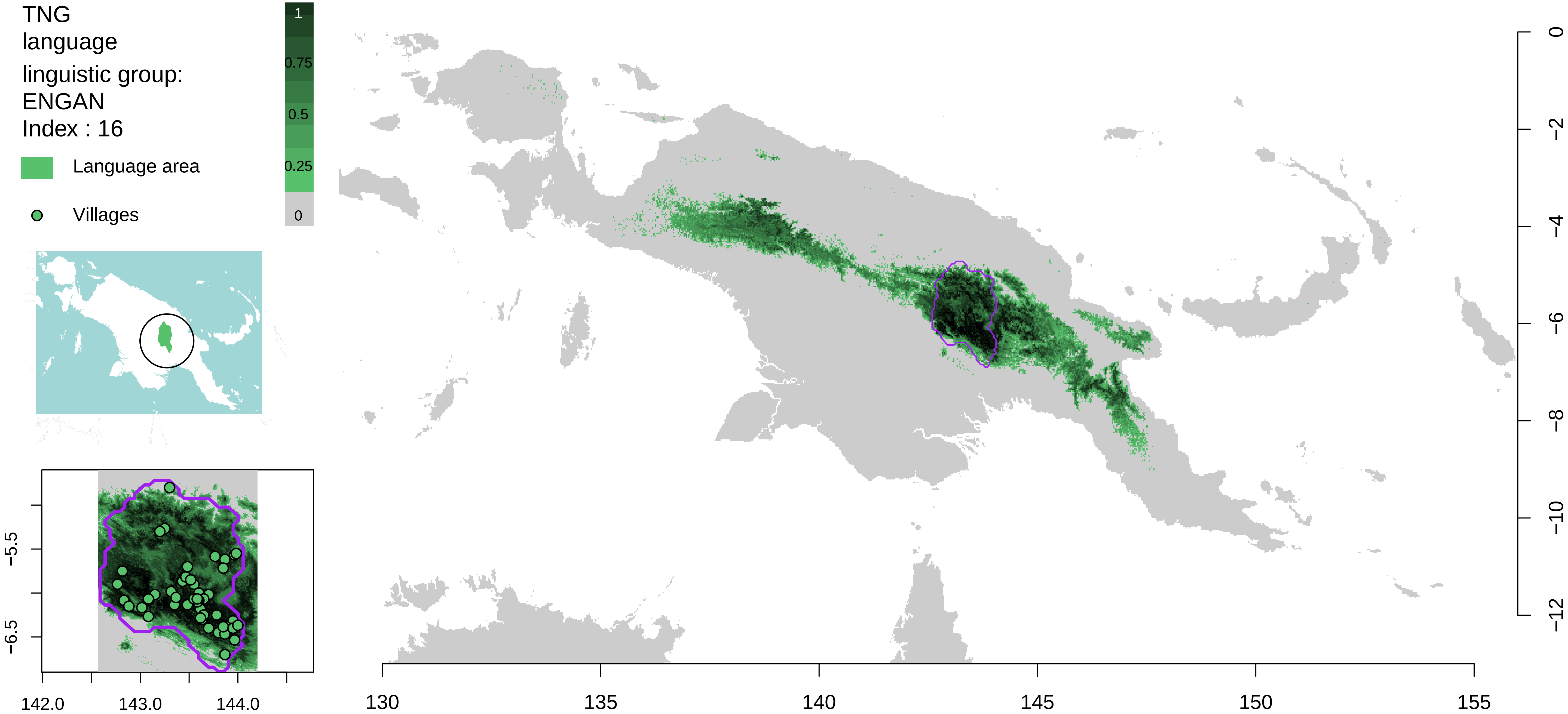

TNG
language
linguistic group:
ENGAN
Index : 16
Language area
Villages
1
0.75
0.5
0.25
0
−5.5
−6.5
142.0
143.0
144.0
130
135
140
145
150
155
0
−2
−4
−6
−8
−10
−12

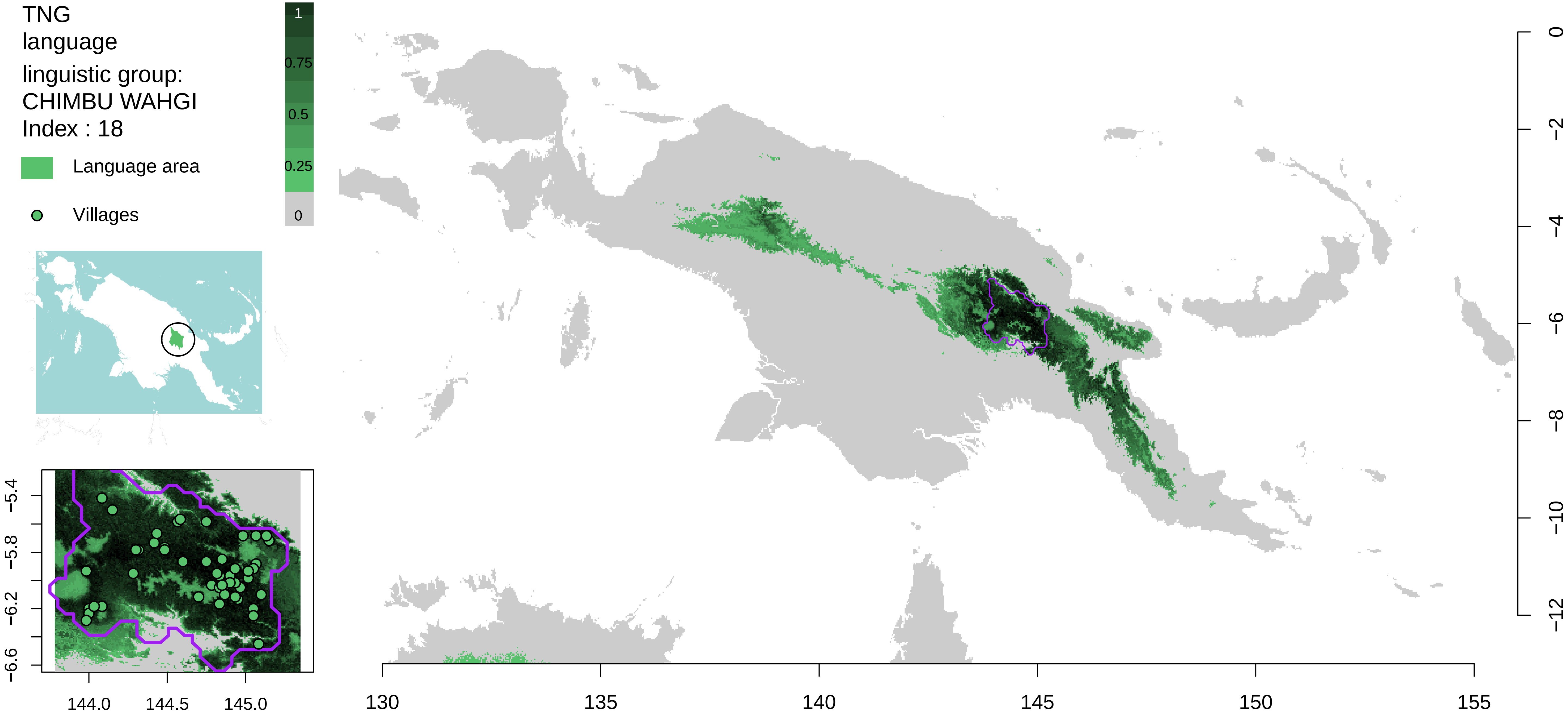
TNG
language
linguistic group:
CHIMBU WAHGI
Index : 18
Language area
Villages
1
0.75
0.5
0.25
0
−5.4
−5.8
−6.2
−6.6
144.0
144.5
145.0
130
135
140
145
150
155
0
−2
−4
−6
−8
−10
−12

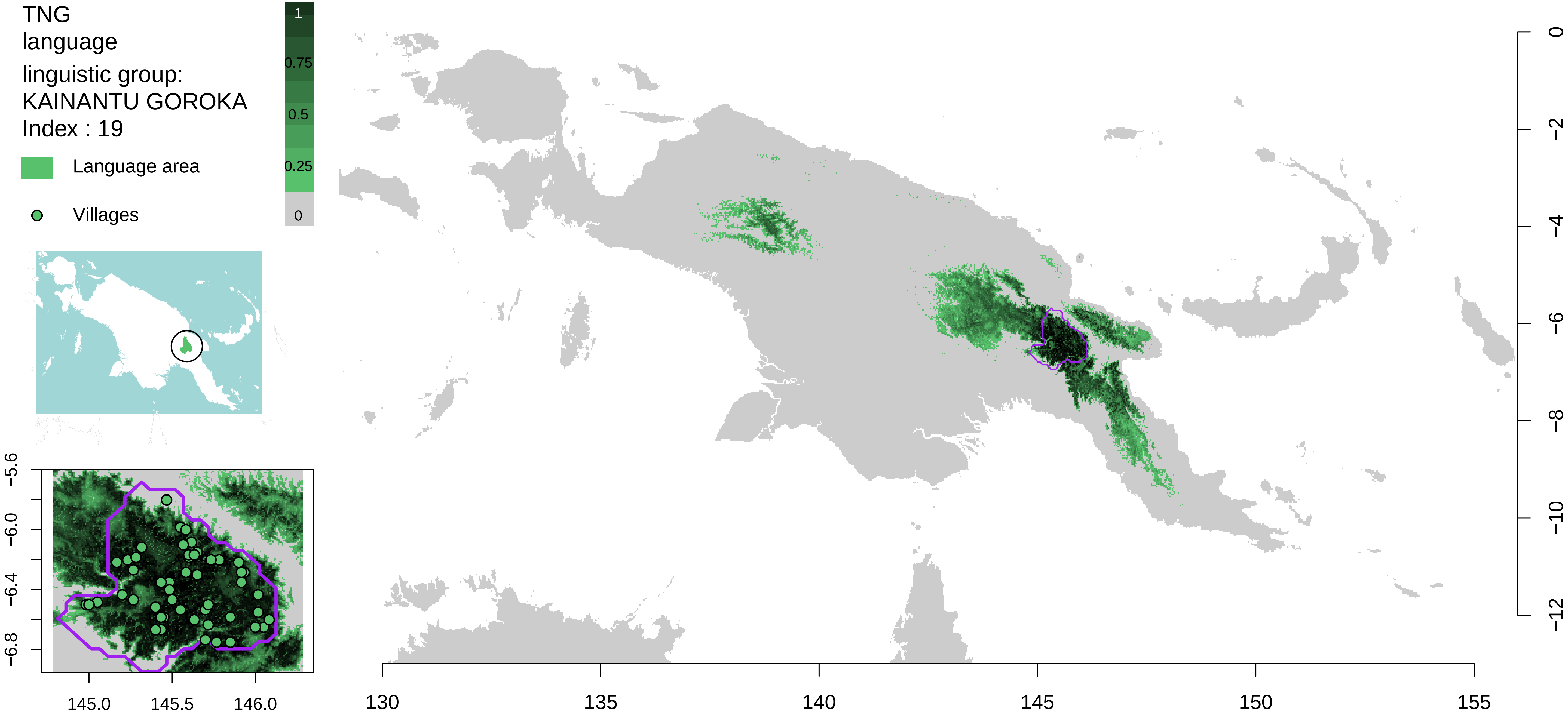
TNG
language
linguistic group:
KAINANTU GOROKA
Index : 19
Language area
Villages
1
0.75
0.5
0.25
0
−5.6
−6.0
−6.4
−6.8
145.0
145.5
146.0
130
135
140
145
150
155
0
−2
−4
−6
−8
−10
−12

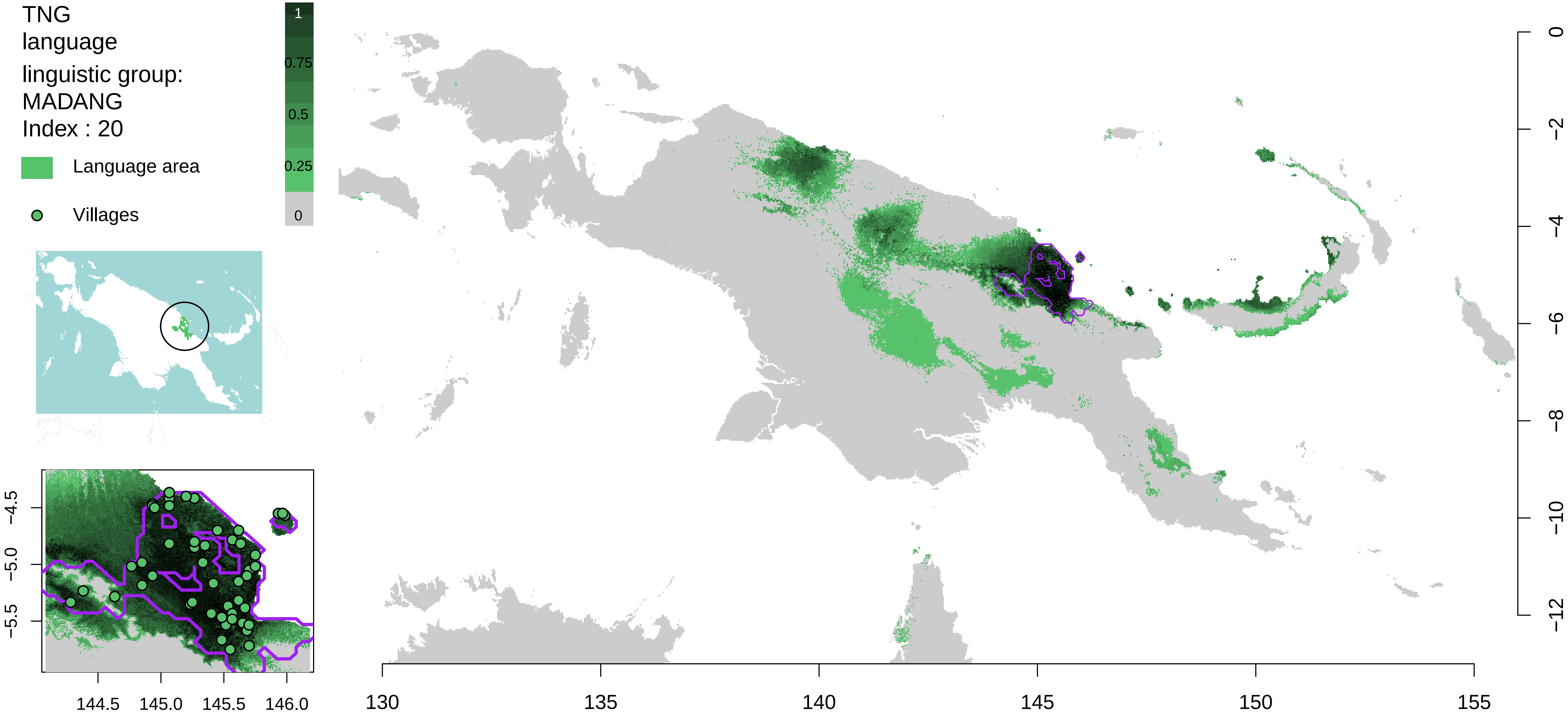
TNG
language
linguistic group:
MADANG
Index : 20
Language area
Villages
1
0.75
0.5
0.25
0
−4.5
−5.0
−5.5
144.5
145.0
145.5
146.0
130
135
140
145
150
155
0
−2
−4
−6
−8
−10
−12

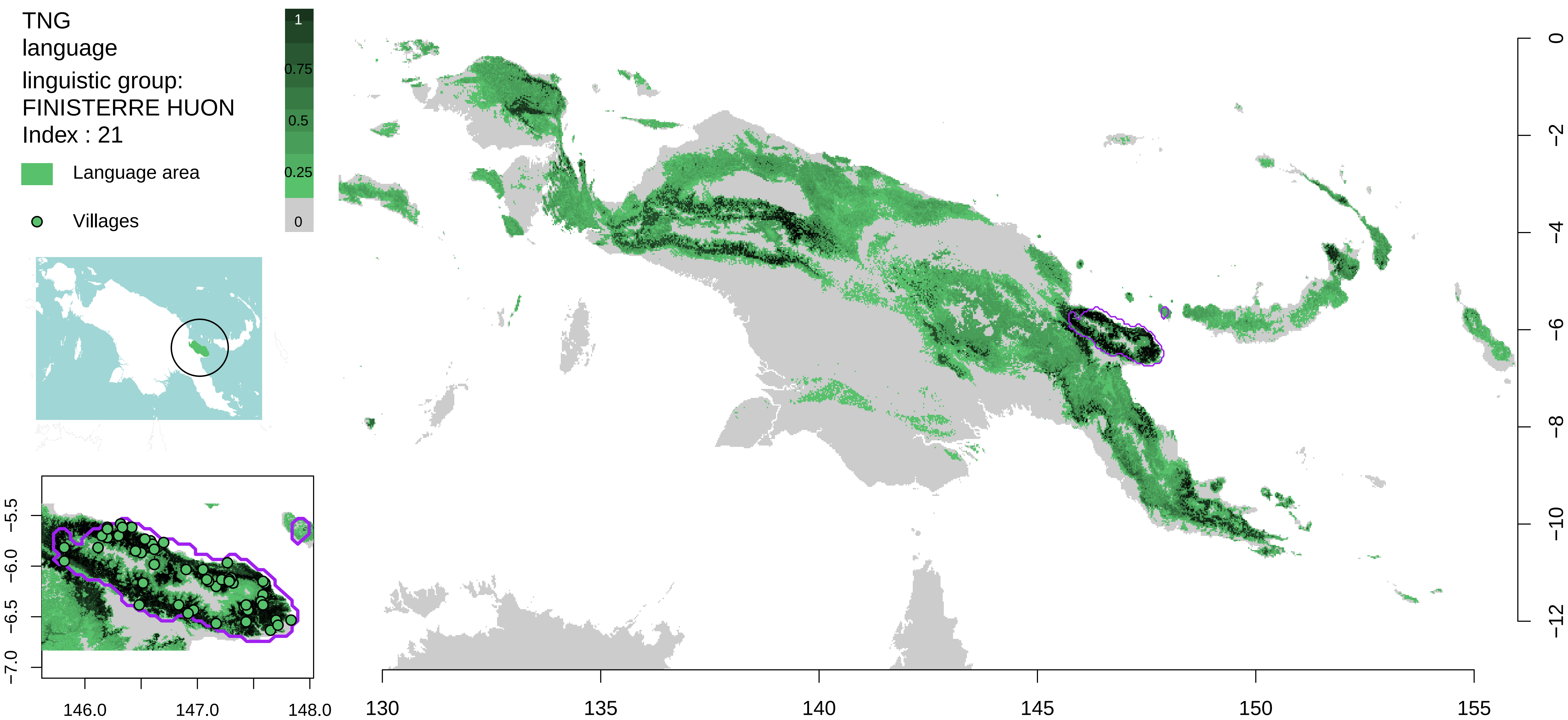

TNG
language
linguistic group:
FINISTERRE HUON
Index : 21
Language area
Villages
1
0.75
0.5
0.25
0
−5.5
−6.0
−6.5
−7.0
146.0
147.0
148.0
130
135
140
145
150
155
0
−2
−4
−6
−8
−10
−12

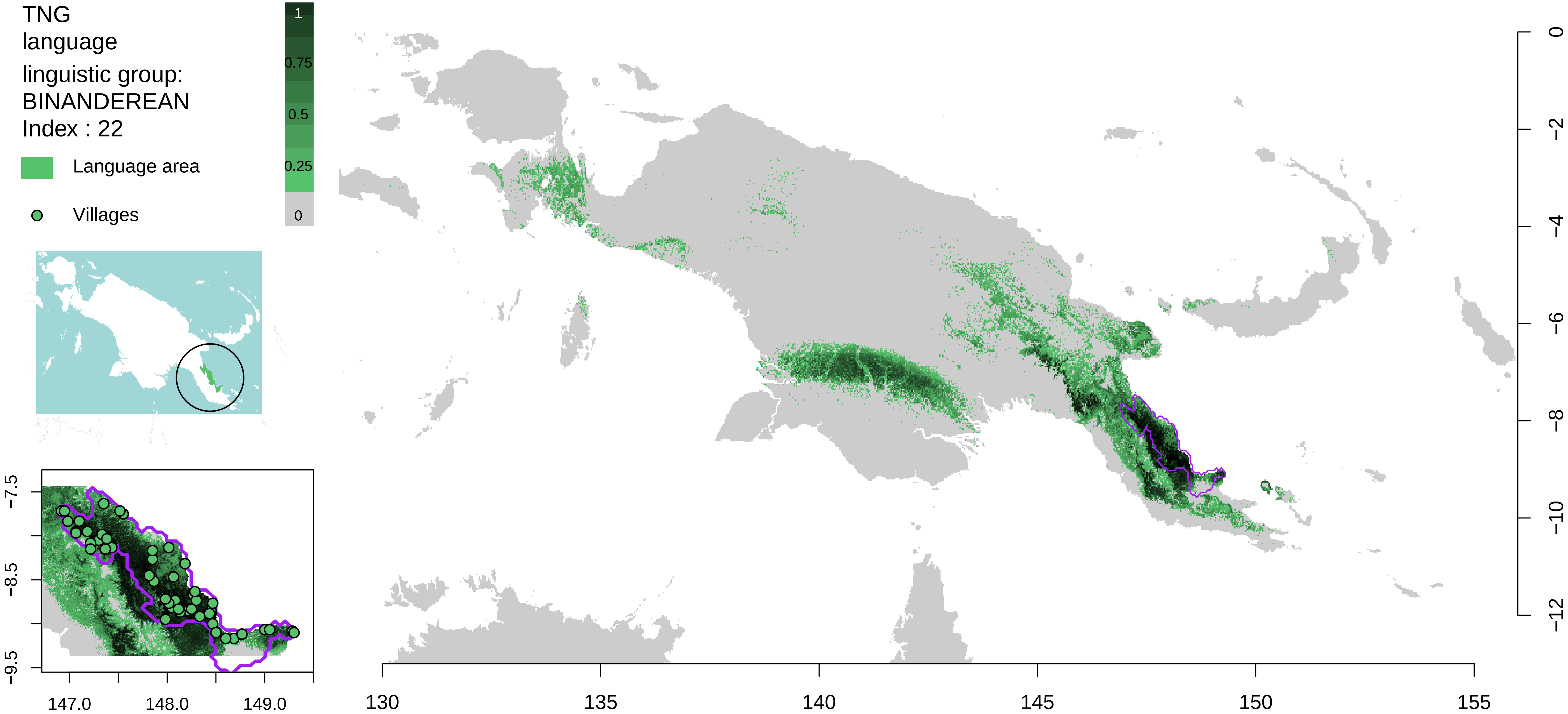
TNG
language
linguistic group:
BINANDEREAN
Index : 22
Language area
Villages
1
0.75
0.5
0.25
0
−7.5
−8.5
−9.5
147.0
148.0
149.0
130
135
140
145
150
155
0
−2
−4
−6
−8
−10
−12

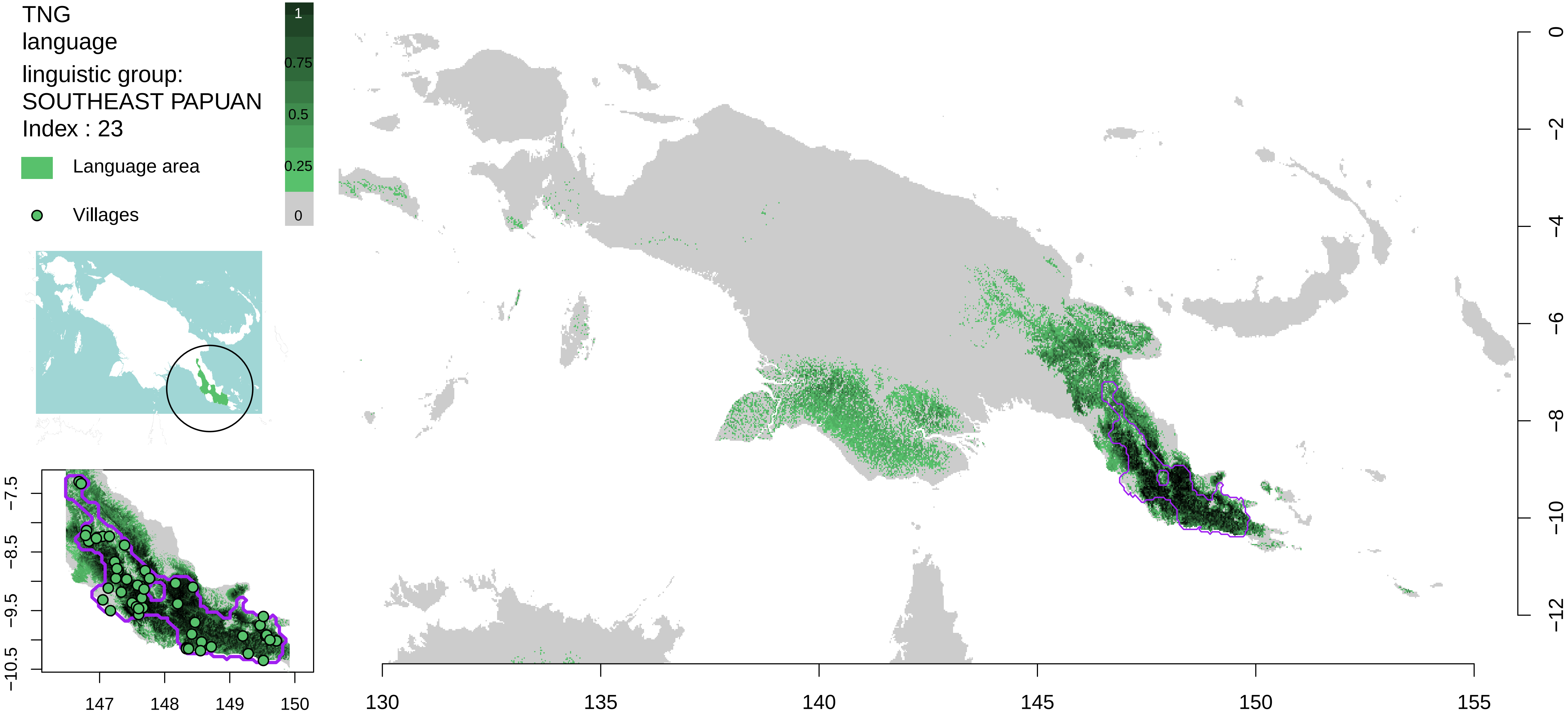

TNG
language
linguistic group:
SOUTHEAST PAPUAN
Index : 23
Language area
Villages
1
0.75
0.5
0.25
0
130
135
140
145
150
155
0
−2
−4
−6
−8
−10
−12
147
148
149
150
−7.5
−8.5
−9.5
−10.5

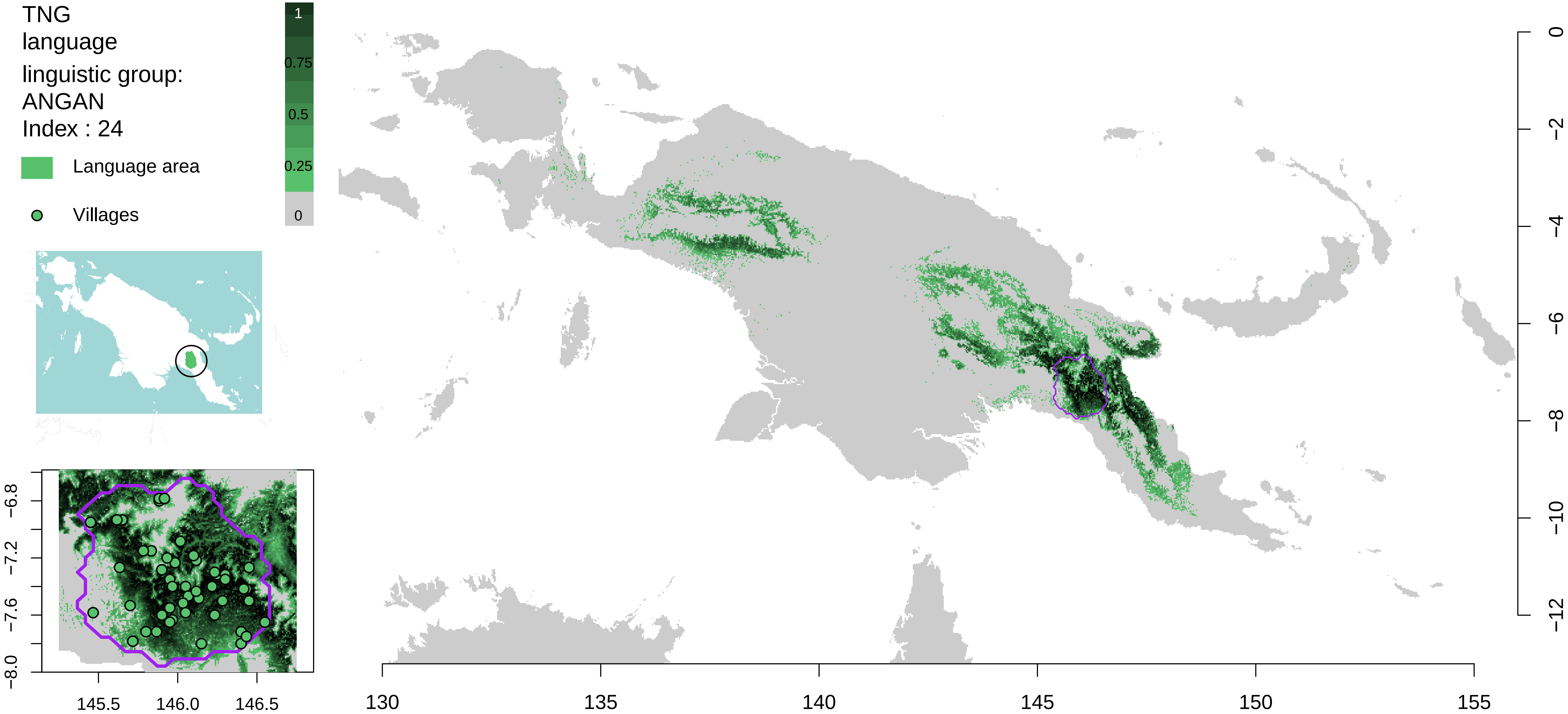

TNG
language
linguistic group:
ANGAN
Index : 24
Language area
Villages
1
0.75
0.5
0.25
0
−6.8
−7.2
−7.6
−8.0
145.5
146.0
146.5
130
135
140
145
150
155
0
−2
−4
−6
−8
−10
−12

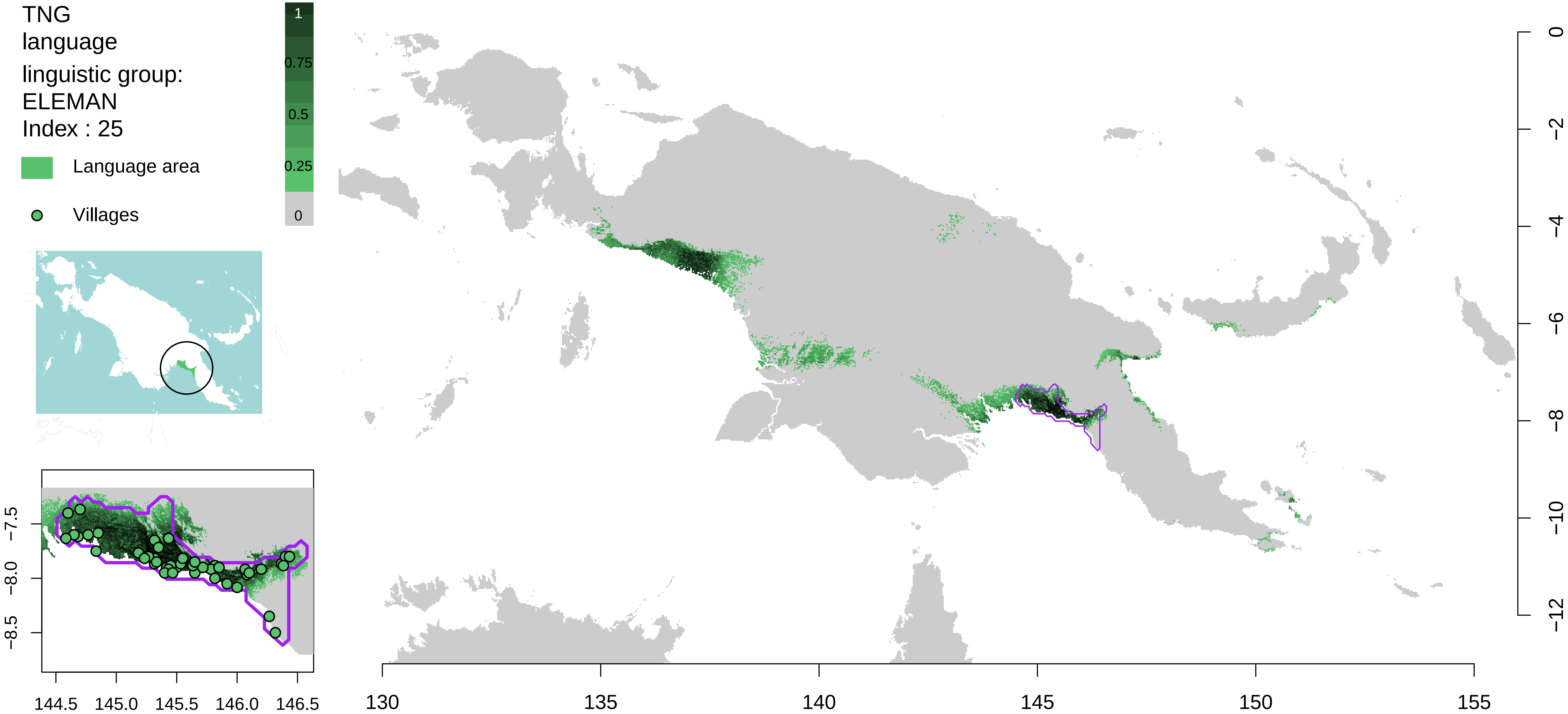
TNG
language
linguistic group:
ELEMAN
Index : 25
Language area
Villages
1
0.75
0.5
0.25
0
−7.5
−8.0
−8.5
144.5
145.0
145.5
146.0
146.5
130
135
140
145
150
155
0
−2
−4
−6
−8
−10
−12

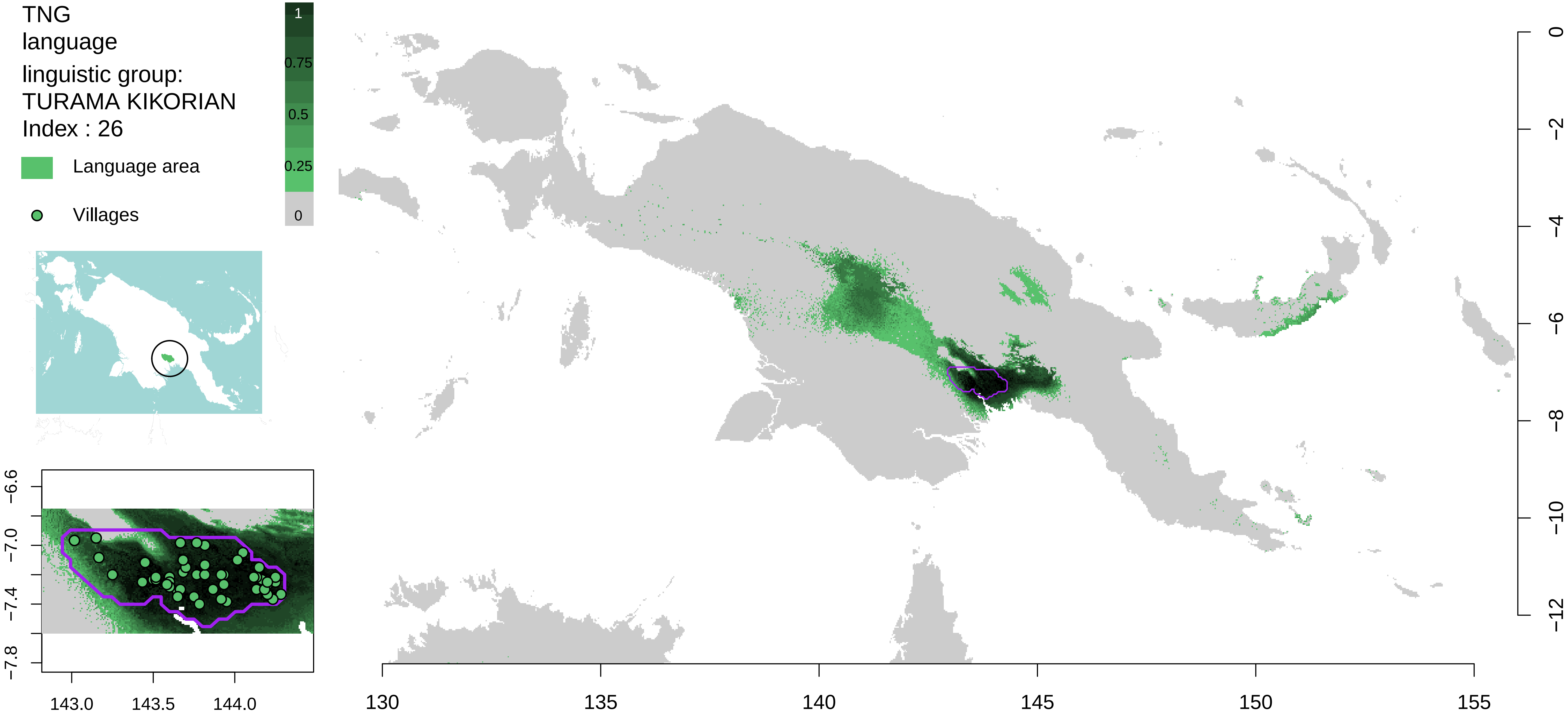
TNG
language
linguistic group:
TURAMA KIKORIAN
Index : 26
Language area
Villages
1
0.75
0.5
0.25
0
−6.6
−7.0
−7.4
−7.8
143.0
143.5
144.0
0
−2
−4
−6
−8
−10
−12
130
135
140
145
150
155

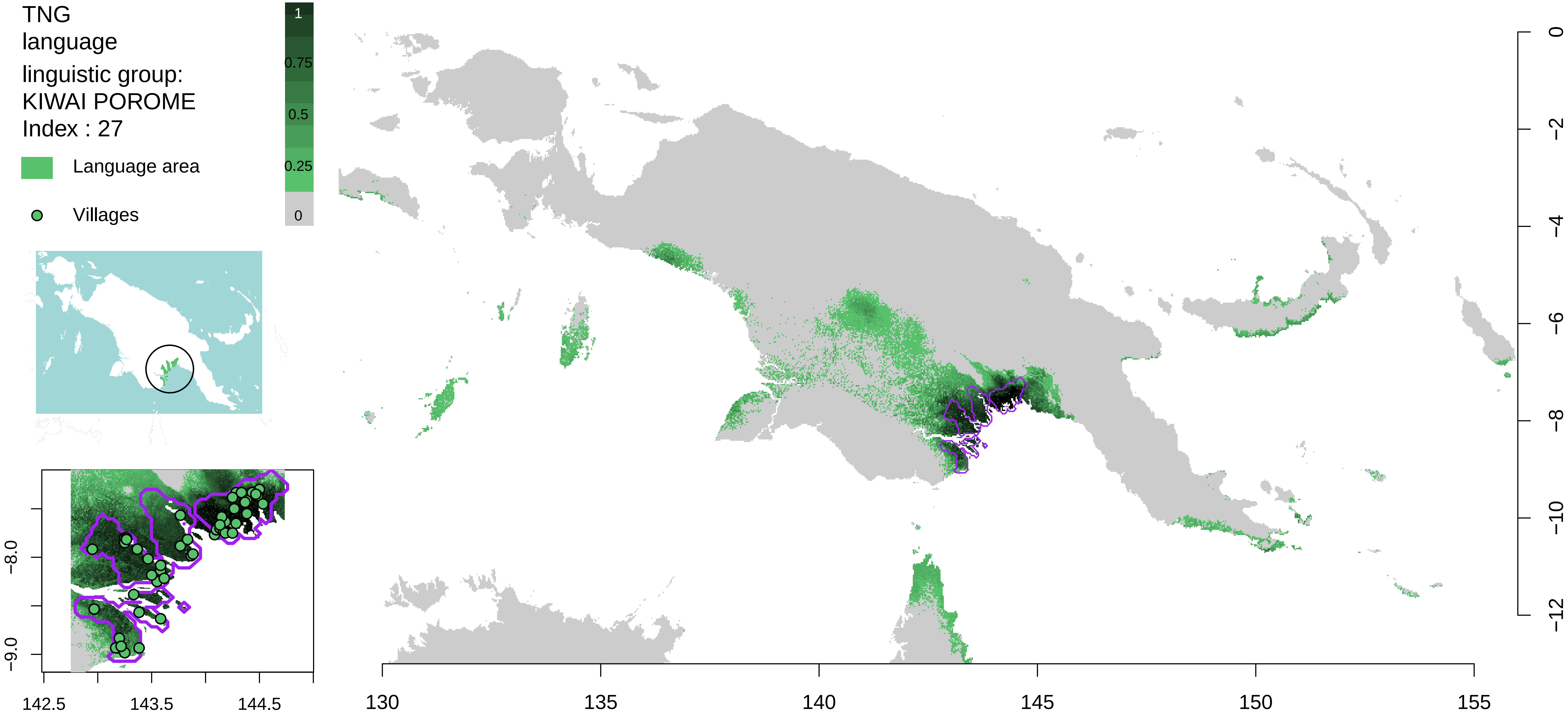

TNG
language
linguistic group:
KIWAI POROME
Index : 27
Language area
Villages
1
0.75
0.5
0.25
0
−8.0
−9.0
142.5
143.5
144.5
130
135
140
145
150
155
0
−2
−4
−6
−8
−10
−12

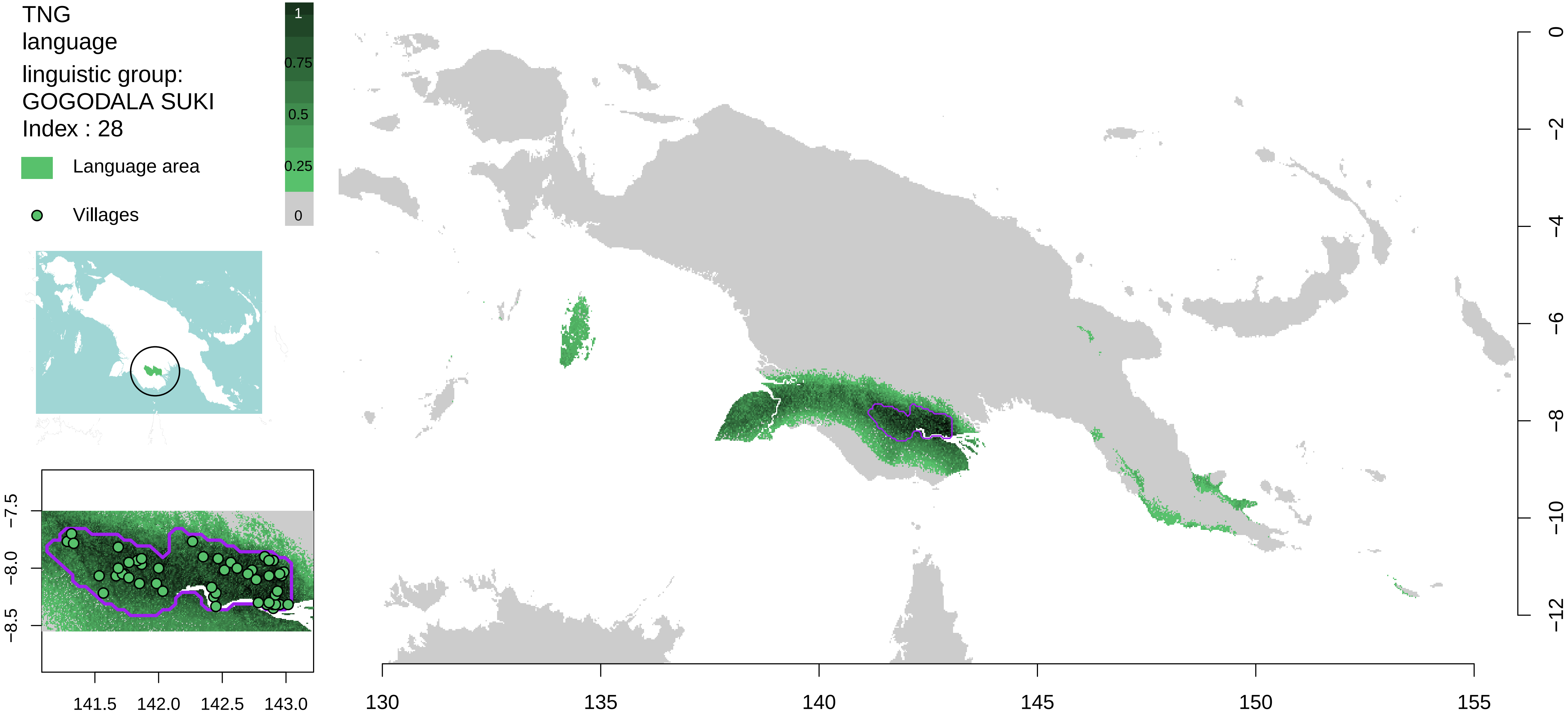

TNG
language
linguistic group:
GOGODALA SUKI
Index : 28
Language area
Villages
1
0.75
0.5
0.25
0
−7.5
−8.0
−8.5
141.5
142.0
142.5
143.0
130
135
140
145
150
155
0
−2
−4
−6
−8
−10
−12

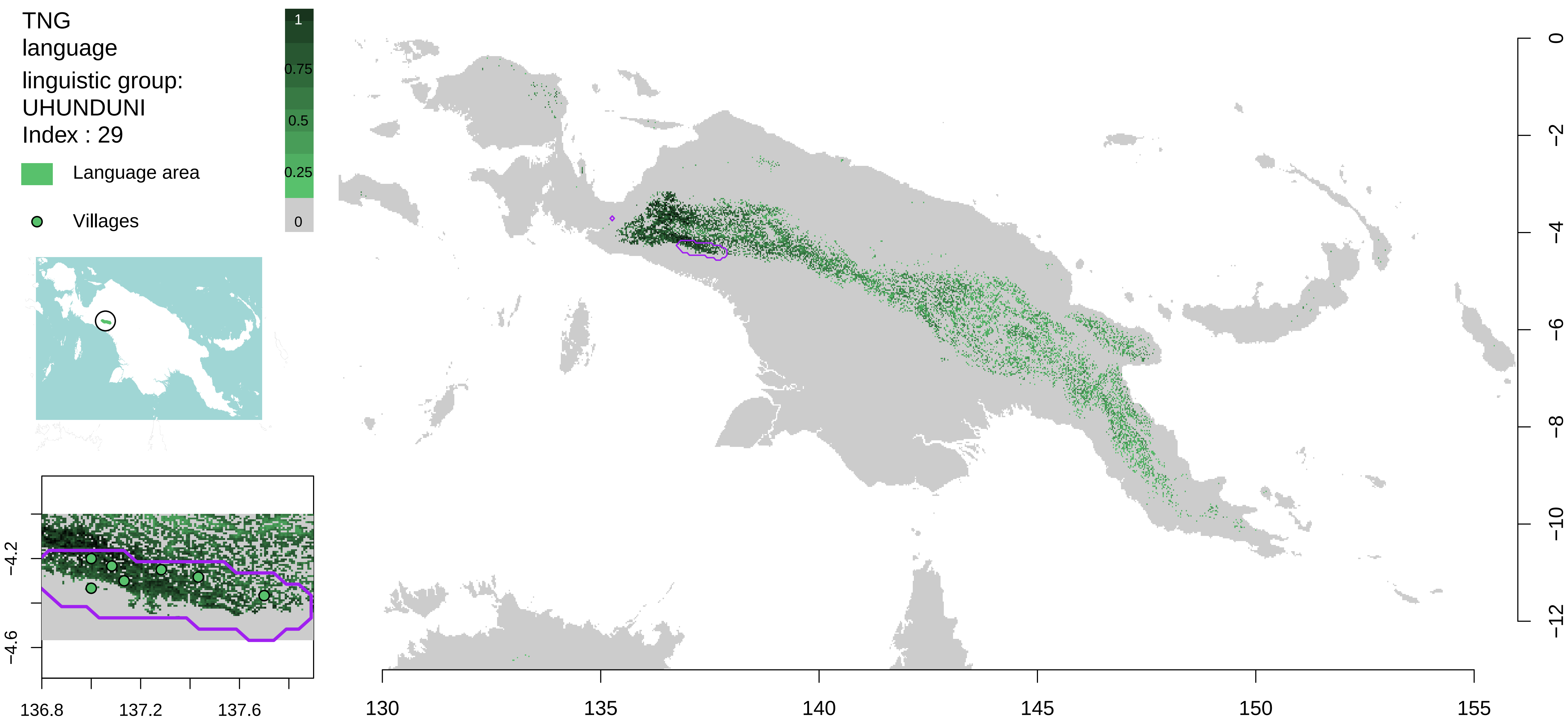

TNG
language
linguistic group:
UHUNDUNI
Index : 29
Language area
Villages
1
0.75
0.5
0.25
0
−4.2
−4.6
136.8
137.2
137.6
130
135
140
145
150
155
0
−2
−4
−6
−8
−10
−12

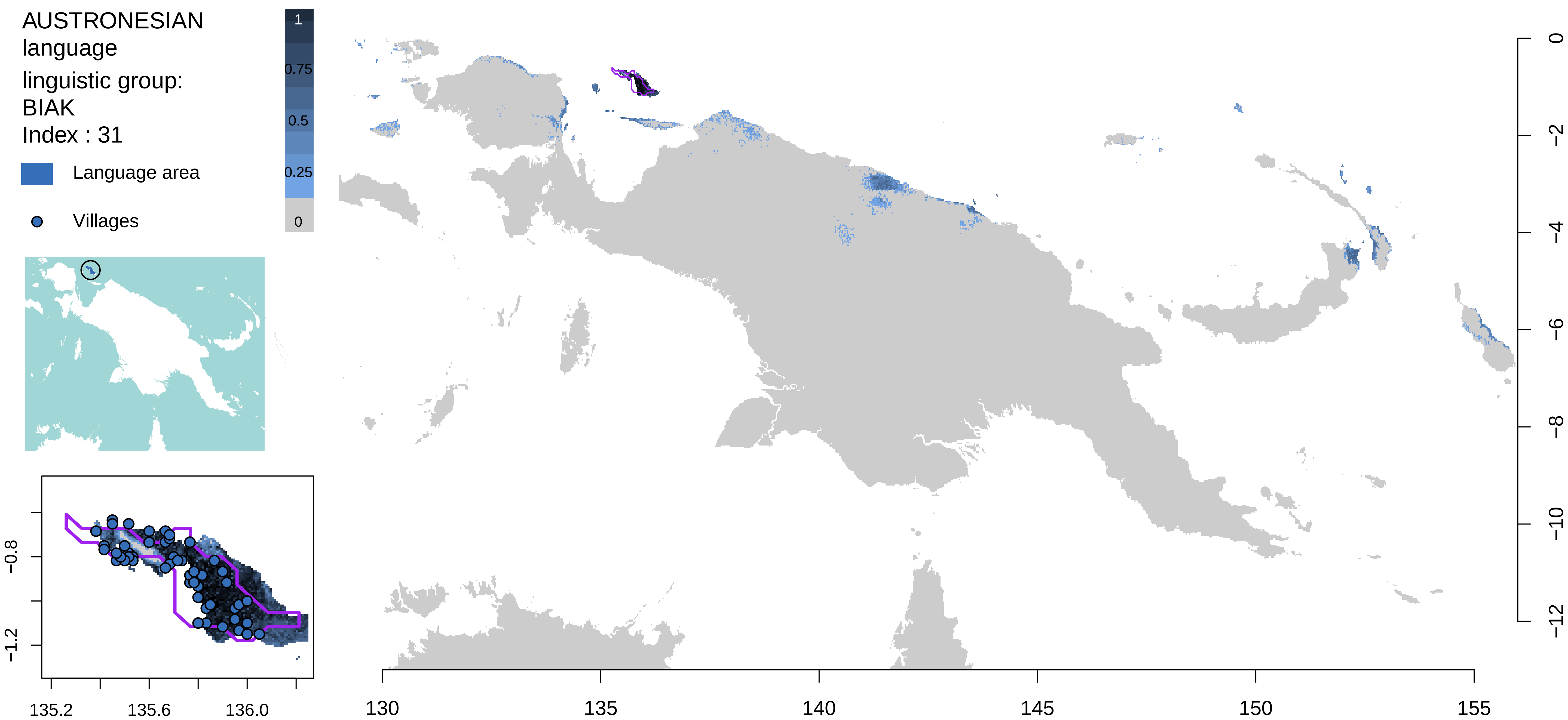

AUSTRONESIAN
language
linguistic group:
BIAK
Index : 31
Language area
Villages
1
0.75
0.5
0.25
0
−0.8
−1.2
135.2
135.6
136.0
130
135
140
145
150
155
0
−2
−4
−6
−8
−10
−12

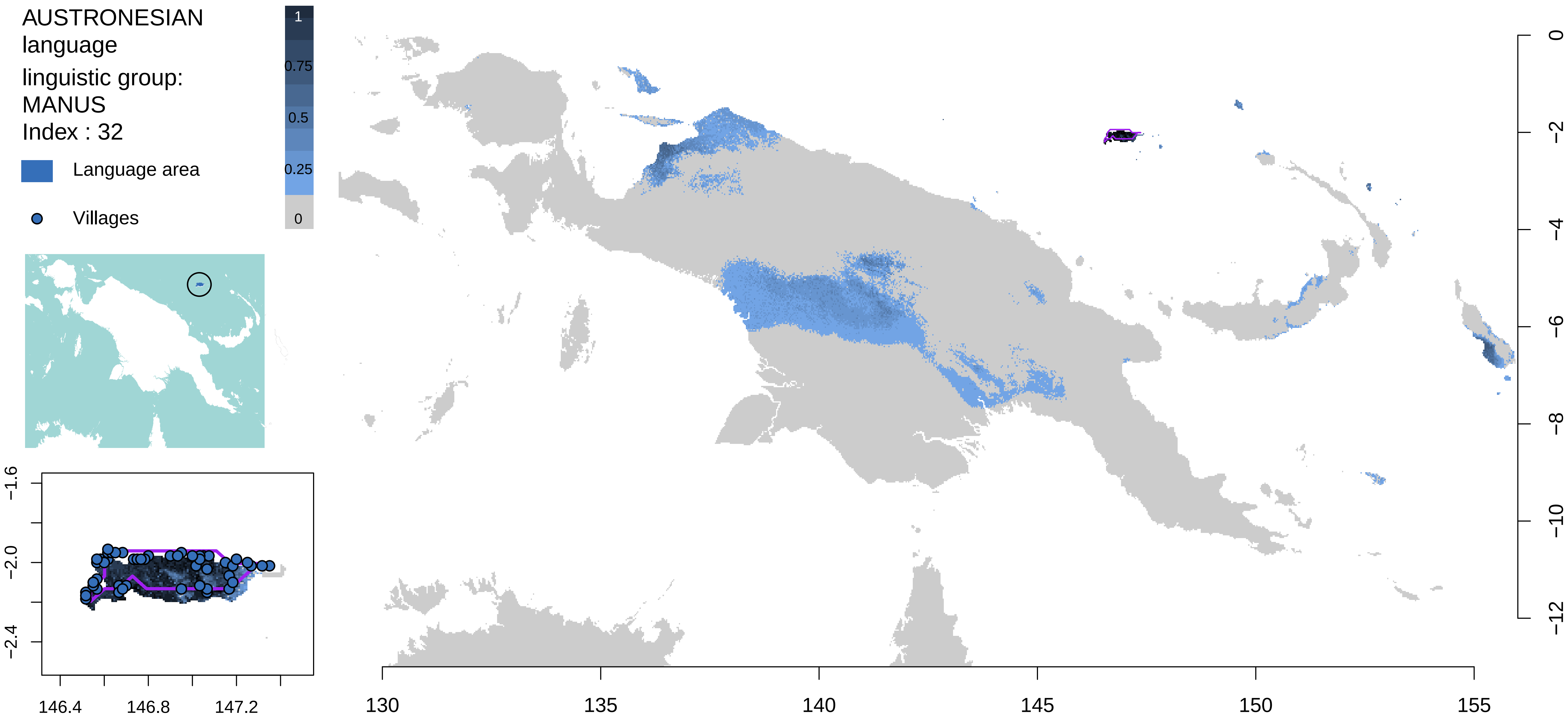
AUSTRONESIAN
language
linguistic group:
MANUS
Index : 32
Language area
Villages
1
0.75
0.5
0.25
0
−1.6
−2.0
−2.4
146.4
146.8
147.2
130
135
140
145
150
155
0
−2
−4
−6
−8
−10
−12

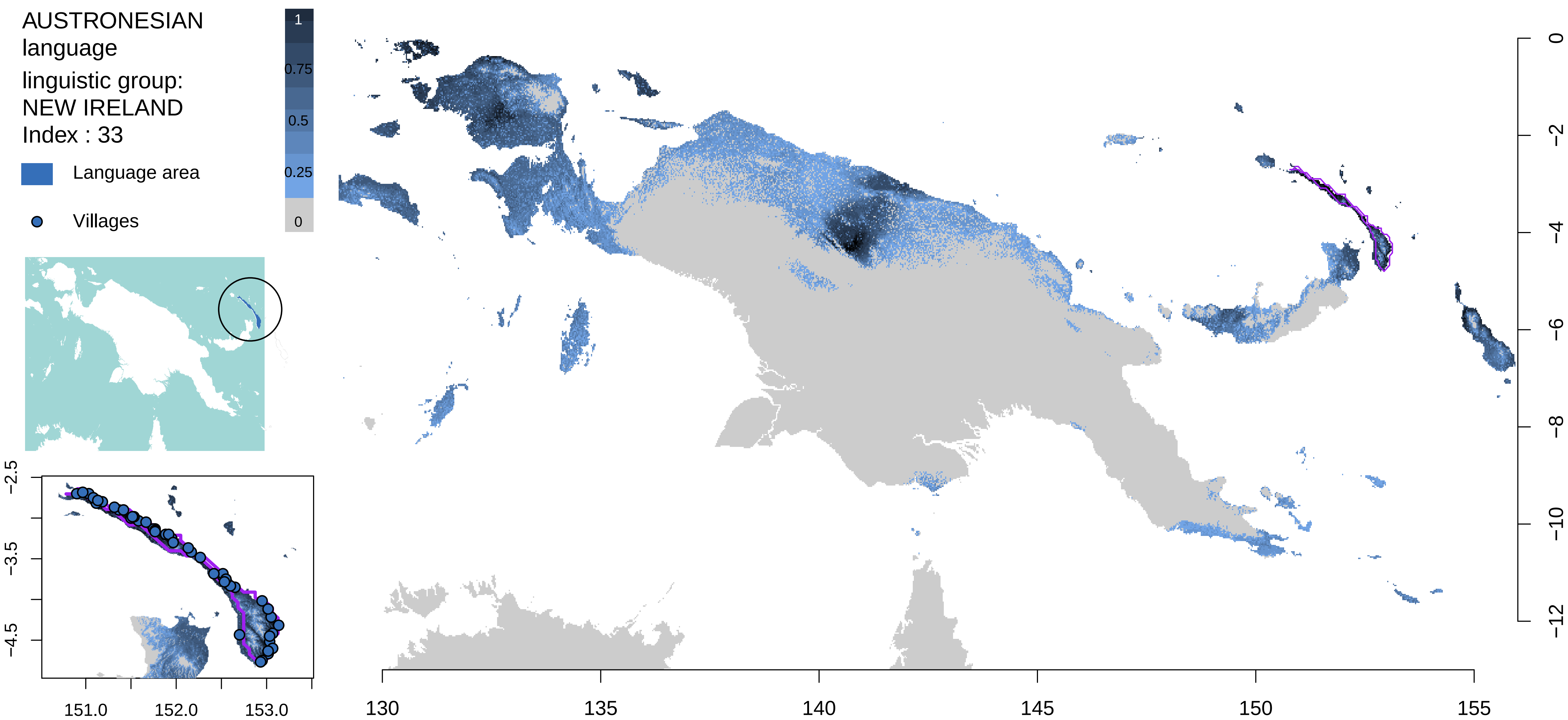

AUSTRONESIAN
language
linguistic group:
NEW IRELAND
Index : 33
Language area
Villages
1
0.75
0.5
0.25
0
−2.5
−3.5
−4.5
151.0
152.0
153.0
130
135
140
145
150
155
0
−2
−4
−6
−8
−10
−12

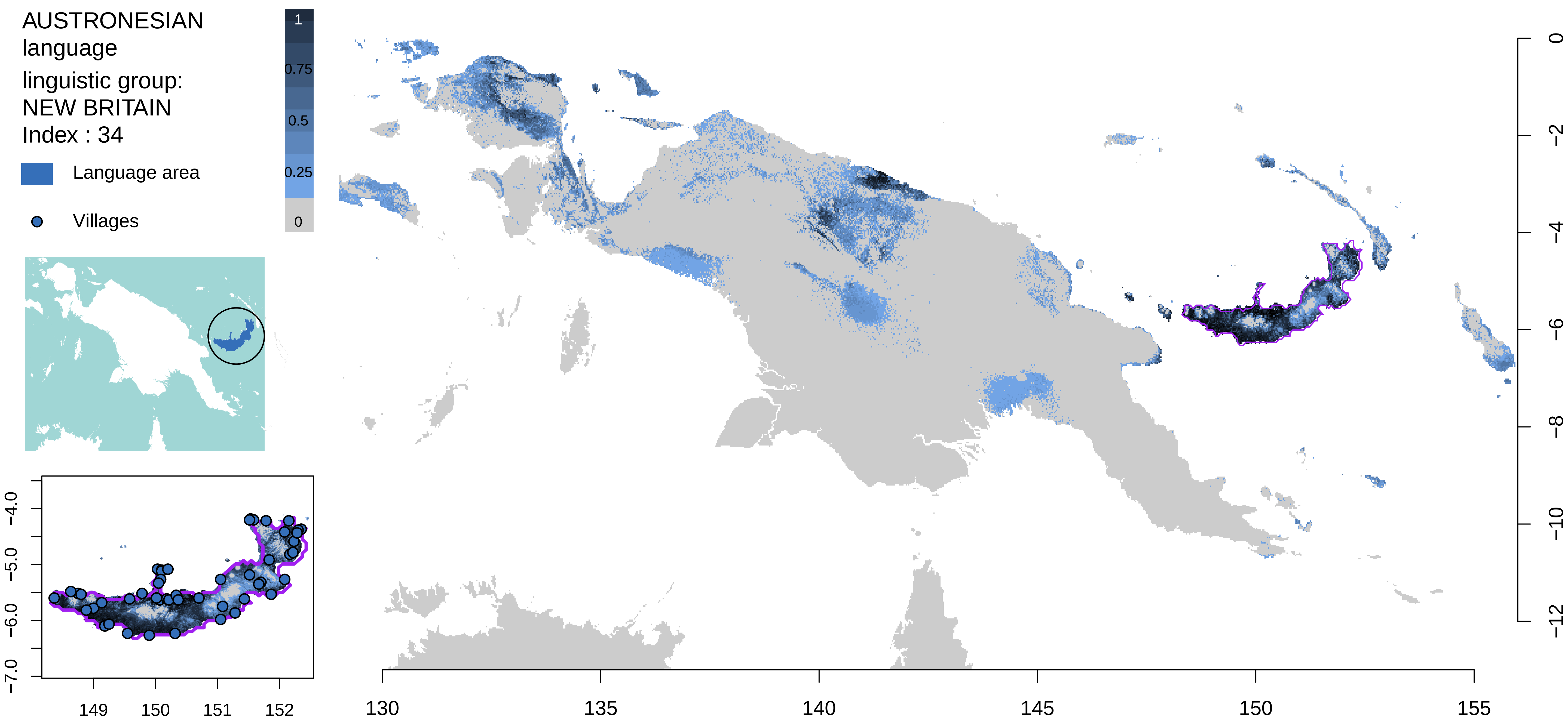

AUSTRONESIAN
language
linguistic group:
NEW BRITAIN
Index : 34
Language area
Villages
1
0.75
0.5
0.25
0
−4.0
−5.0
−6.0
−7.0
149
150
151
152
130
135
140
145
150
155
0
−2
−4
−6
−8
−10
−12

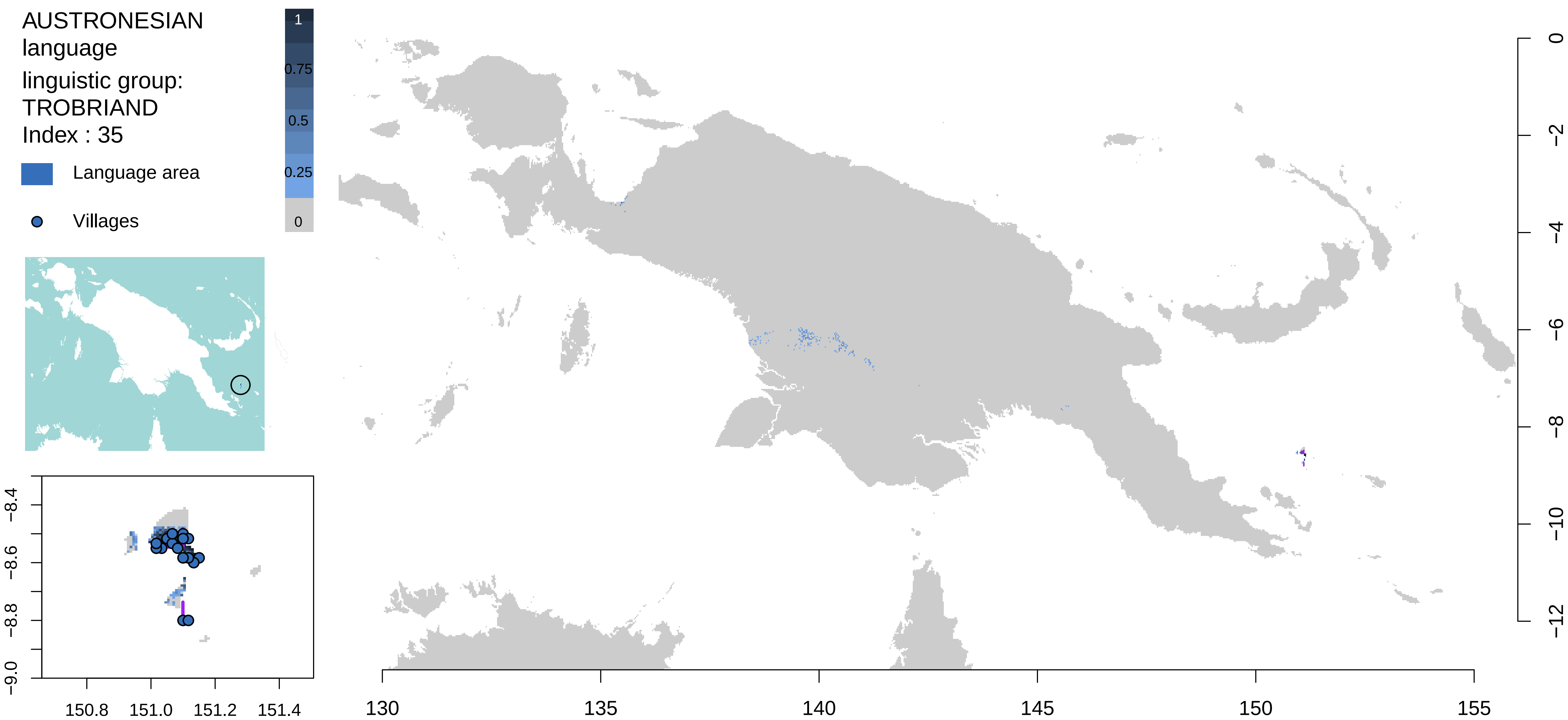
AUSTRONESIAN
language
linguistic group:
TROBRIAND
Index : 35
Language area
Villages
1
0.75
0.5
0.25
0
−8.4
−8.6
−8.8
−9.0
150.8
151.0
151.2
151.4
130
135
140
145
150
155
0
−2
−4
−6
−8
−10
−12

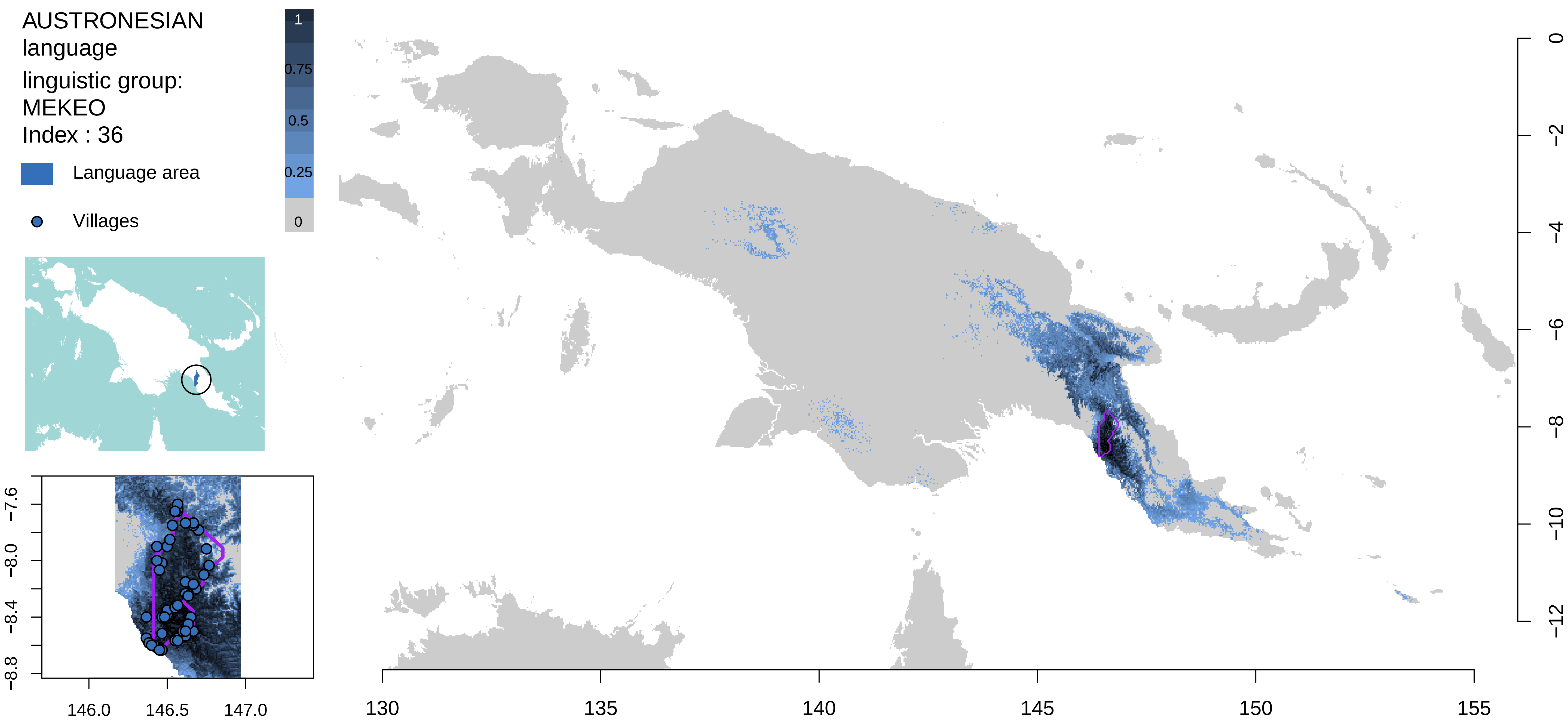
AUSTRONESIAN language
linguistic group: MEKEO
Index : 36
Language area
Villages
1
0.75
0.5
0.25
0
−7.6
−8.0
−8.4
−8.8
146.0
146.5
147.0
130
135
140
145
150
155
0
−2
−4
−6
−8
−10
−12

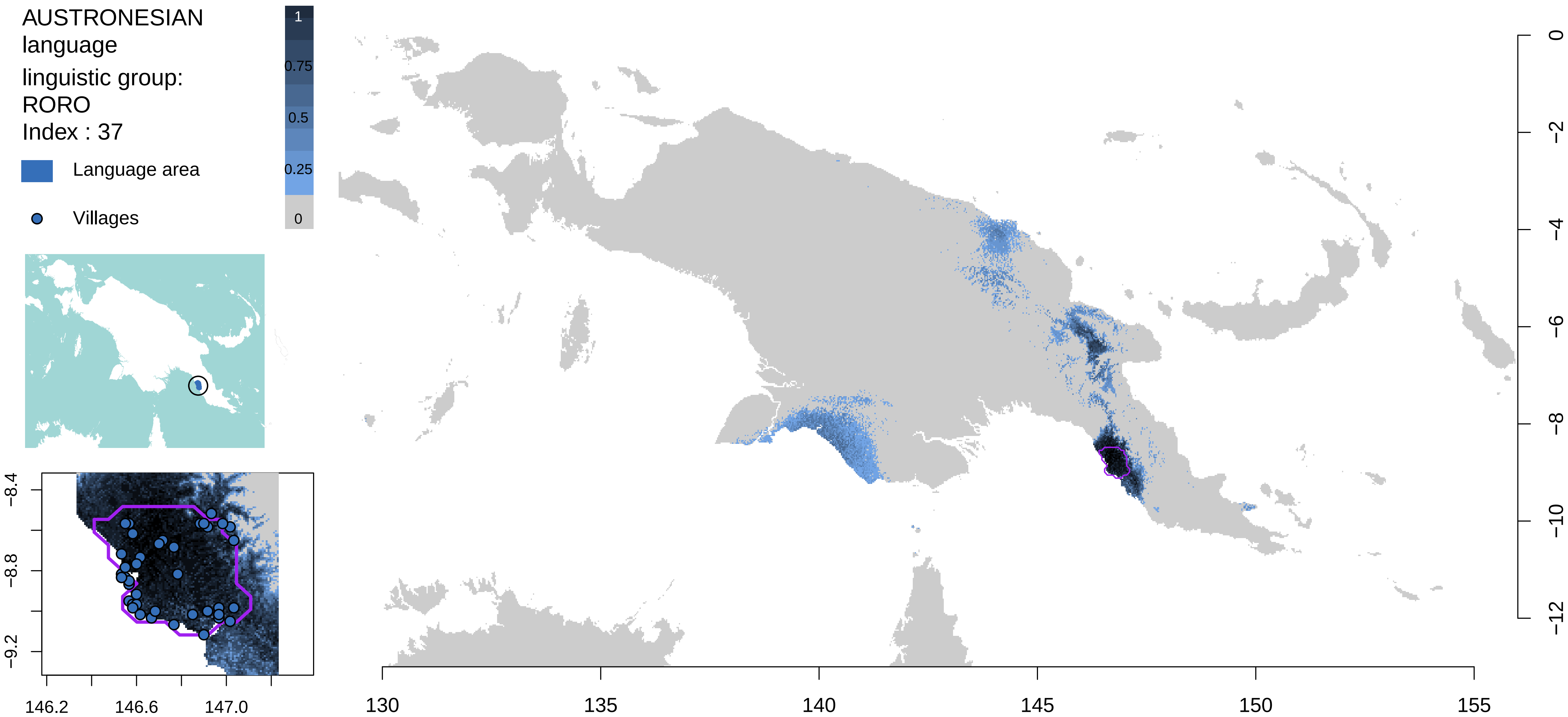

AUSTRONESIAN
language
linguistic group:
RORO
Index : 37
Language area
Villages
1
0.75
0.5
0.25
0
−8.4
−8.8
−9.2
146.2
146.6
147.0
130
135
140
145
150
155
0
−2
−4
−6
−8
−10
−12

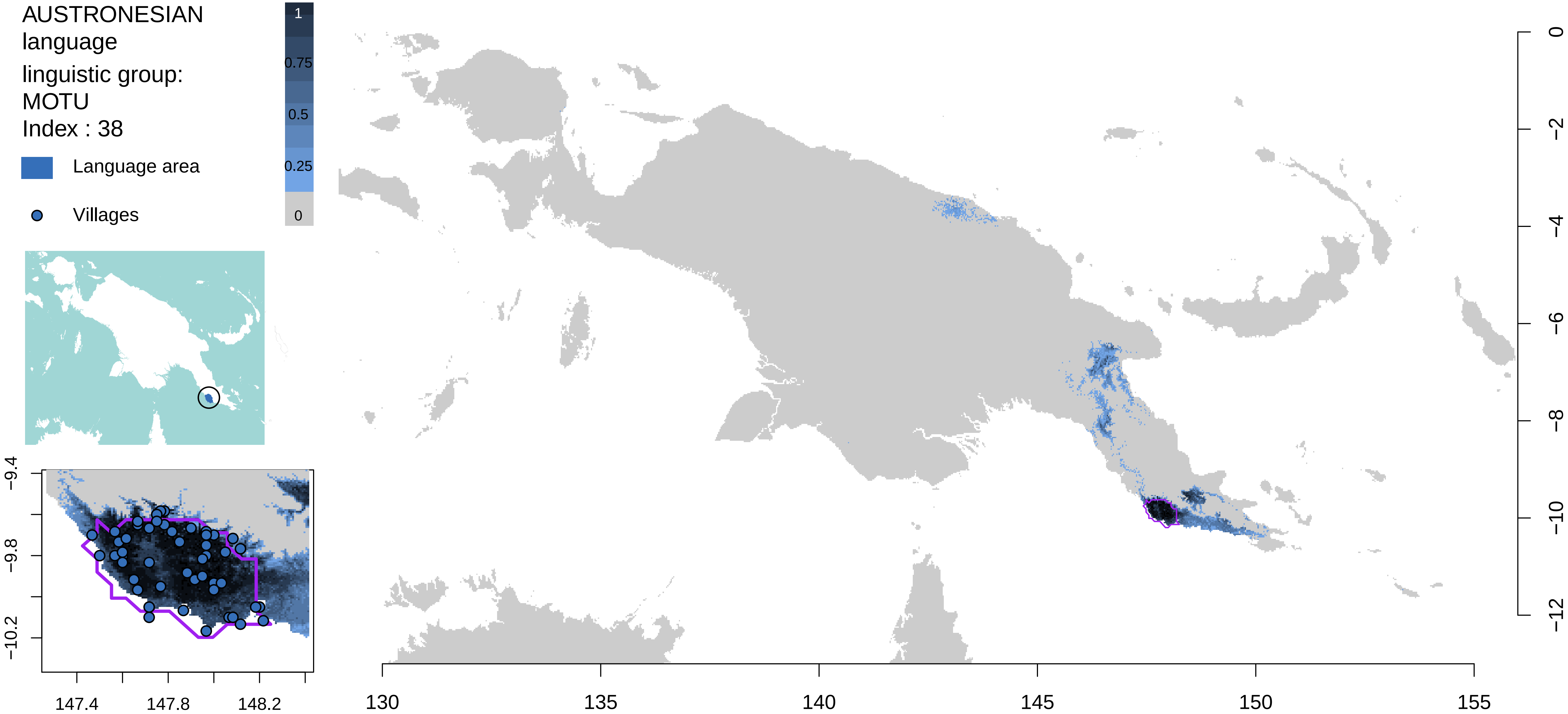

AUSTRONESIAN
language
linguistic group:
MOTU
Index : 38
Language area
Villages
1
0.75
0.5
0.25
0
−9.4
−9.8
−10.2
147.4
147.8
148.2
130
135
140
145
150
155
0
−2
−4
−6
−8
−10
−12

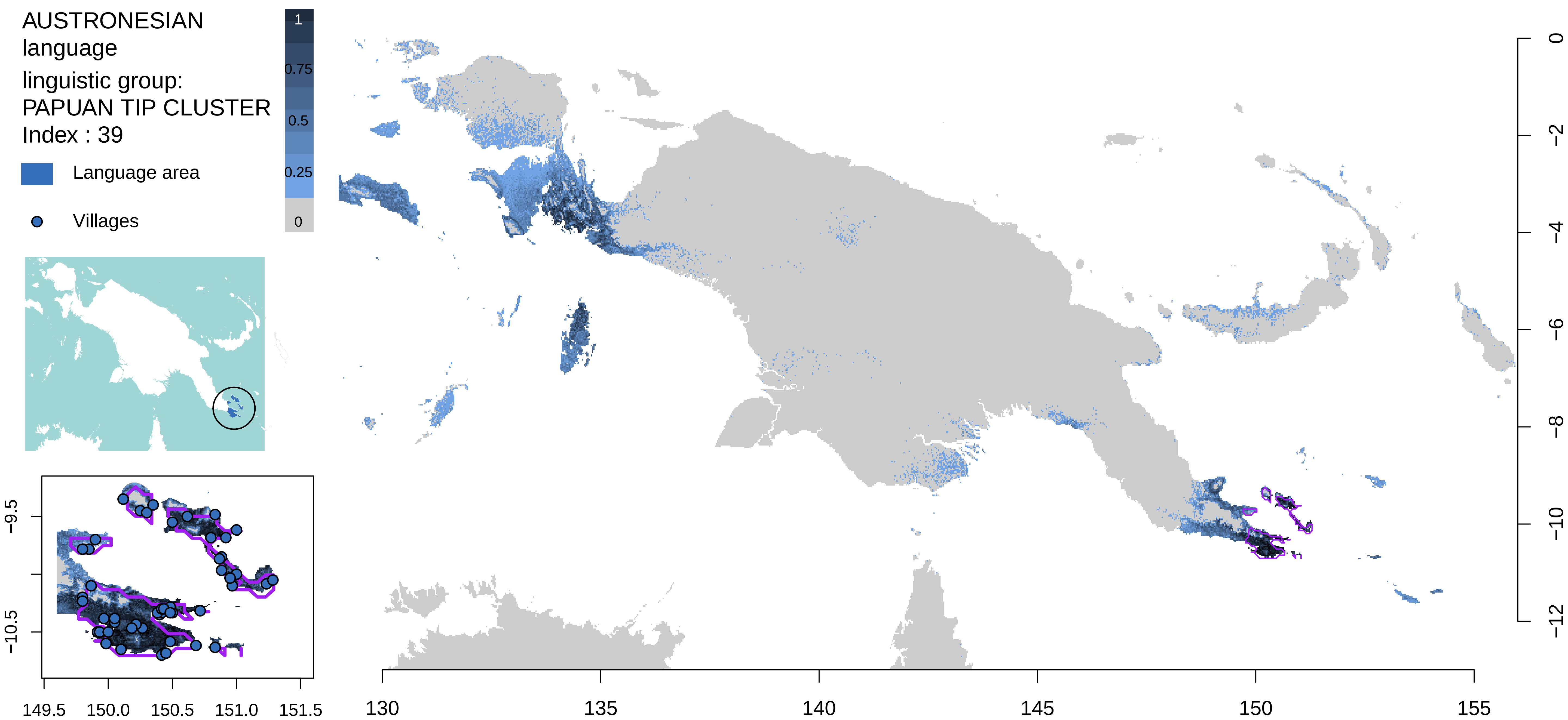
AUSTRONESIAN
language
linguistic group:
PAPUAN TIP CLUSTER
Index : 39
Language area
Villages
1
0.75
0.5
0.25
0
−9.5
−10.5
149.5
150.0
150.5
151.0
151.5
130
135
140
145
150
155
0
−2
−4
−6
−8
−10
−12
