# Supplementary material for: Quantitative methods demonstrate that environment alone is an insufficient predictor of present-day language distributions in New Guinea
Source: PLoS One. 2020 Oct 7;15(10):e0239359. doi: 10.1371/journal.pone.0239359 (PMC7540881; doi:10.1371/journal.pone.0239359)
Supplement: S1 File — Eco-Linguistic Niche Modeling (ELNM); Text 2. Papuan and Austronesian settlement, language diversity and phylogeny in New Guinea (NG); Table 1. Characterisation of the Eco-Linguistic Niche (ELN) of each language group; Table 2. Matrices of overlap values between Eco-linguistic niches of language groups; Table 3. Algorithms from OpenModeler with their parameter settings used for the prediction of Eco-Linguistic Niches of language groups; Table 4. Description of the consensus building method used for Eco-Linguistic Niches Modeling and obtained values for Accuracy (ACC), Area Under the “Receiver Operating Characteristics (ROC)” Curve (AUC) and Partial ROC ratio (P-ROC-ratio); Table 5. Phylolinguistic classification of Papuan Trans New Guinean language groups; Table 6. Geographic coordinates of the selected villages. (PDF) [file pone.0239359.s002.pdf]

PLOS ONE

S2 File. Supporting Information for:

Quantitative methods demonstrate that environment alone is an insufficient predictor of present-day language distributions in New Guinea

Nicolas Antunes, Wulf Schiefenhövel, Francesco d'Errico, William E. Banks, Marian Vanhaeren

Correspondence to: antunes@rgzm.de

List of Supplementary Texts

1. Eco-Linguistic Niche Modelling (ELNM) 2
2. Settlement, language diversity and phylogeny in New Guinea (NG) 3

List of Tables

1 Characterisation of the Eco-Linguistic Niche (ELN) of each language group 6
2 Matrices of overlaps between Eco-Linguistic niches 7
3 Algorithms parameter settings 8
4 Consensus building method 9
5 Phylolinguistic classification of Trans New Guinean language groups 23
6 Geographical coordinates of villages 26

S2 Text 1. Eco-Linguistic Niche Modelling (ELNM)

Our approach, derived from Ecological Niche Modelling (ENM) developed by biodiversity researchers [1, 2], allows one to correlate linguistic diversity with a range of environmental variables.

Over the last decade, ENM has been applied to past human populations [3–13], termed Eco-Cultural Niche Modelling (ECNM), in order to investigate the potential influence of environmental factors on cultural trajectories and evaluate the suitability of specific environments for specific cultures. ECNM may also inform on settlement history. For example, for the Norse colonization of Greenland, this method has allowed to evaluate the impact of climate change and the identification of the most influential environmental factors on the Norse settlement history in that region [14].

ENM and ECNM employ algorithms based on various statistical and mathematical methods (e.g. [15–22]) to establish predictions. Numerous algorithms have been developed, each of which has its own specificities and performance varies according to the environments and the characteristics of modeled populations [23]. The use of several different algorithms can generate nuanced niche predictions for the same population and the same environment. Algorithms providing the best predictions may be different according to the case study. However, there is no algorithm that provides optimal predictions in all circumstances) [23]. To improve precision, authors either use selected algorithms [24, 25] and multiply models to select the most robust predictions [23] or produce a consensus prediction by combining predictions from several architectures [26–30].

Application of these algorithms in ecology requires geographic coordinates of the locations where a target species has been observed, and raster GIS layers summarising environmental dimensions potentially relevant to shaping the geographic distribution of that species. Similarly, the data required for ECNM include geographical coordinates of historically or archaeologically visible cultural traits and the palaeoenvironmental variables (climatic and topographic) that potentially played a role in the geographical distribution of the human population linked to those traits.

ECNM has been profiled as a promising approach to identify environmental and cultural mechanisms behind cultures trajectories [10]. The present study represents the first attempt to apply ECNM to New Guinean languages. We use a new consensus method [31], which evaluates the performance of each algorithm and retains only the most robust predictions for each language

group niche.

S2 Text 2. Settlement, language diversity and phylogeny in New Guinea (NG)

Linguistic studies, often in combination with genetics and/or archaeology, have been used to reconstruct the settlement of NG and, in particular, the expansion of the Austronesian language family throughout the Pacific and Indian oceans [32–39].

More than one thousand traditional languages were spoken in NG when scientists started to document the linguistic diversity of the world's second largest island in the 20th century [40–45]. They are attributed to the Papuan (sometimes the term Non-Austronesian languages of New Guinea is used) and Austronesian language families [41, 44, 46]. The Austronesian language family constitutes a monophyletic clade, i.e. the similarities between the constituent languages indicate that they derive from the same common ancestral language [44, 47–51]. The Papuan languages constitute, to the contrary, a paraphyletic clade, i.e. they belong to different language families, which do not share enough common features to trace them back to a single common origin. Papuan languages include the large Trans New Guinea (TNG) language family, and 22 smaller languages families as well as 9 to 13 language isolates [44, 52].

Classification of the Papuan languages varies according to authors and investigated linguistic feature (pronouns, phonemes, cognates, grammatical construction, and lexicon) [36, 42–44]. For example, Wurm's [1975] [41] and Ross' [2005] [44] disagree on the languages that must be considered as members of the Trans New Guinea (TNG) language family. Furthermore, in the more recent record, named Glottolog of the World's languages [53], the unity of the TNG family is only partially maintained in the relabeled Nuclear Trans New Guinean (NTNG) language family. A systematic classification of Papuan languages based on multiple linguistic features and an in-depth linguistic comparative method [54] is still absent and it is clear that the existing classifications, identifying between 10 [41], 40-60 [42] and 70 [53] Papuan language families and up to 68 isolates [53], are not definite.

Papuan languages are traditionally linked to the first human populations thought to arrive in NG through island hopping via present-day Indonesia [55, 56] some 50 000 years ago [39, 41, 42, 44, 46, 57–67]. The Papuan TNG family, currently occupying most of the interior of New Guinea, is possibly the third largest in the world with 400 languages and is tentatively thought

to have originated with root-crop agriculture around 10,000 years ago [68–74]. However, little is known about the history of this language family [45].

Recently, a consortium of researchers in genetics of human populations [75] concluded that the expansion of agricultural human groups from a single founder population could explain the present day genetic structure of Papua New Guinea populations, thereby confirming previous results [76] of an isolated evolution of human populations in the Sahul for most of the last 50 ky and the independence of New Guinea from Australia for much of this time [76, 77]. In addition, genetic studies of individuals belonging to 85 language groups of Papua New Guinea show that highland populations had a demographic growth and grew isolated from the lowland populations for some 10-20,000 years [76]. Earlier this year, another study concluded that two distinct Denisovan lineages contributed to present day Papuan genomes suggesting a more complex settlement history of Melanesia [Jacobs et al. 2019]. The large genetic diversity both in highland and lowland populations points, however, to an absence of massive population replacements and a cultural-linguistic rather than terrain driven differentiation [76]. These results confirm that the correlation between languages, cultures and genes is worthy to be studied in NG as in other regions of the world.

The Austronesian language family has been brought to NG much more recently, some 3500 years ago, by populations with elaborate navigation techniques [33, 44, 49, 66, 78–83]. The precise modalities of the Pleistocene and Holocene colonisations of NG are still largely unknown, and it is not clear if all non-Austronesian languages of NG (i.e., Papuan languages) are linked to a single event or the different Papuan language families may be linked to multiple waves of genetically distinct populations [44, 53]. Similarly, several hypotheses (e.g. “Nusantao” [84], “Pacific Express Train” [78], “Slow Boat” [85], “Triple I” [86], “Slow Train” [87], “Entangled Bank” [88], and “Pulse Pause” [37]) have been proposed for the origin and expansion of Austronesian speaking populations in NG and other Islands of the Pacific and Indian oceans. The more than 300 Austronesian languages of mainland and island NG belong to the Central-Eastern Malayo-Polynesian subgroup of the Austronesian language family [44, 89, 90]. The Eastern Malayo-Polynesian subgroup comprises two separate Phyla, Greater South Halmahera-West New Guinea and Oceanic [53].

Population genetics in Papua New Guinea (PNG) have shown that all lowland populations bear various degrees of South East Asian ancestry; not surprisingly these traces are particularly present in populations who speak Aus-

tronesian languages [76]. Among the non-Austronesian language groups in the lowlands of PNG, the northern groups speaking Sepik-Ramu languages show the lowest rates of such admixture [76]. As mentioned above, autochthonous NG highlanders show no Austronesian genes [76]. Friedländer et al. [2008] [91] have shown that groups in the Bismarck Archipelago have a great genetic diversity. Some groups there are of Austronesian ancestry, whereas Papuan speaking groups on New Britain and, to a lesser degree, groups on Bougainville and New Ireland, are genetically different among themselves and from the Papuan groups who today live in the main island of New Guinea.

S2 Table 1: Characterisation of the Eco-Linguistic Niche (ELN) of each language group. "Case 1": geographically continuous ELN prediction covering an area much larger than the linguistic area. "Case 2": ELN prediction and linguistic area largely coincide. Number of languages and philolinguistic ranks were calculated according to Glottolog 3.0 [53]

Language group	Linguistic area (km^2)	ELN surface (km^2)	Linguistic area / ELN surface	Case	n lang.	Philolinguistic Rank
00-West TNG linkage	50102	119353	0.42	1	22	-
08-Mek	10394	234309	0.04	1	8	2
09-Asmat-Kamoro	52395	240063	0.22	2	11	3
10-Awyu-Domot	35601	249765	0.14	1	20	3
11-Ok	20990	149481	0.14	1	18	3
12-Marind	36244	172476	0.21	1	2	3
15-Bosavi	10705	101551	0.11	1	2	3
16-Engan	24182	143940	0.17	1	6	3
18-Chimbu-Wahgi	15073	118860	0.13	1	17	2
19-Kainantu-Goroka	10136	114272	0.09	1	28	2
20-Madang	14103	228634	0.06	1	107	2
21-Finisterre-Huon	14859	606106	0.02	1	61	2
22-Binanderean	13071	239265	0.05	1	13	2
23-Southeast-Papuan	31085	222765	0.14	1	30	na
24-Angan	13229	159471	0.08	1	13	1
25-Eleman	8522	105925	0.08	2	5	1
26-Turama-Kikorian	6048	153728	0.04	1	4	1
27-Kiwai-Porome	11787	249608	0.05	1	6	1
28-Gogodala-Suki	10266	111260	0.09	1	4	1
29-Uhunduni	2918	66722	0.04	1	1	1
31-Biak	2804	50918	0.06	2	1	10
32-Manus	2009	162475	0.01	2	23	8
33-New Ireland	7461	307388	0.02	1	21	6
34-New Britain	36217	220078	0.16	1	38	6
35-Trobriand	336	7353	0.05	2	1	10
36-Mekeo	3707	124863	0.03	1	5	11
37-Roro	4365	174135	0.03	1	3	10
38-Motu	4323	51321	0.08	1	1	11
39-Papuan tip	9131	181430	0.05	2	43	8

na: not applicable

S2 Table 2: Two matrices of overlap values, the Schoener's D (top right) and the Hellinger I (bottom left) distances, between Eco-Linguistic niches of language groups.

	31 Biak	32 Manus	33 New Ireland	34 New Britain	35 Trobriand	36 Mekeo	37 Roro	38 Motu	39 Papuan ticluster	00 West TNG linkage	08 Mek	09 Asmat Kamoro	10 Awyu Domot	11 Ok	12 Marind	15 Bosavi	16 Engan	18 Chimbu Wahgi	19 Kainantu Goroka	20 Madang	21 Finisterre Huon	22 Binanderean	23 Southeast Papuan	24 Angan	25 Eleman	26 TuramKikorian	27 Kiwai Porome	28 GogodalSuki	29 Uhunduni
**31**	1	0.65	0.78	0.71	0.6	0.56	0.55	0.62	0.64	0.57	0.59	0.63	0.57	0.52	0.52	0.49	0.49	0.49	0.5	0.57	0.67	0.56	0.51	0.55	0.62	0.52	0.53	0.5	0.05
**32**	0.9	1	0.61	0.65	0.63	0.47	0.49	0.5	0.55	0.53	0.61	0.66	0.67	0.68	0.49	0.63	0.48	0.45	0.45	0.63	0.58	0.5	0.44	0.53	0.66	0.73	0.6	0.43	0.05
**33**	0.96	0.88	1	0.75	0.61	0.54	0.55	0.61	0.71	0.57	0.58	0.68	0.57	0.51	0.51	0.47	0.48	0.48	0.49	0.56	0.69	0.56	0.51	0.53	0.62	0.52	0.54	0.51	0.03
**34**	0.93	0.91	0.95	1	0.61	0.5	0.51	0.55	0.66	0.56	0.61	0.66	0.59	0.6	0.49	0.55	0.48	0.47	0.48	0.63	0.67	0.56	0.48	0.57	0.63	0.6	0.56	0.45	0.03
**35**	0.87	0.9	0.89	0.88	1	0.55	0.58	0.57	0.67	0.5	0.53	0.7	0.78	0.55	0.64	0.52	0.45	0.46	0.45	0.55	0.6	0.62	0.55	0.54	0.74	0.65	0.7	0.58	0.04
**36**	0.85	0.79	0.84	0.82	0.84	1	0.75	0.78	0.59	0.63	0.53	0.48	0.53	0.5	0.56	0.53	0.61	0.68	0.71	0.52	0.72	0.68	0.77	0.71	0.56	0.48	0.53	0.59	0.12
**37**	0.85	0.82	0.85	0.82	0.87	0.95	1	0.74	0.61	0.55	0.49	0.5	0.56	0.5	0.63	0.52	0.52	0.57	0.58	0.56	0.65	0.61	0.68	0.59	0.58	0.52	0.61	0.62	0.09
**38**	0.88	0.82	0.88	0.85	0.85	0.96	0.94	1	0.65	0.59	0.52	0.54	0.56	0.49	0.58	0.51	0.53	0.58	0.6	0.52	0.7	0.64	0.73	0.63	0.6	0.5	0.56	0.6	0.09
**39**	0.9	0.86	0.94	0.91	0.91	0.87	0.88	0.91	1	0.54	0.52	0.7	0.65	0.51	0.62	0.5	0.45	0.46	0.47	0.52	0.67	0.62	0.6	0.55	0.67	0.57	0.67	0.64	0.03
**0**	0.85	0.83	0.86	0.84	0.82	0.9	0.84	0.86	0.83	1	0.73	0.5	0.48	0.59	0.44	0.59	0.77	0.69	0.71	0.53	0.75	0.62	0.58	0.73	0.53	0.51	0.46	0.43	0.22
**8**	0.86	0.88	0.86	0.87	0.83	0.84	0.8	0.82	0.81	0.95	1	0.53	0.51	0.7	0.42	0.65	0.7	0.6	0.59	0.59	0.68	0.55	0.5	0.63	0.54	0.58	0.47	0.39	0.18
**9**	0.89	0.91	0.91	0.91	0.93	0.79	0.81	0.83	0.93	0.81	0.83	1	0.7	0.51	0.54	0.48	0.43	0.43	0.43	0.5	0.6	0.57	0.48	0.54	0.75	0.59	0.61	0.5	0.03
**10**	0.85	0.91	0.86	0.88	0.96	0.82	0.85	0.83	0.91	0.8	0.82	0.93	1	0.57	0.68	0.54	0.43	0.45	0.44	0.55	0.59	0.62	0.55	0.52	0.76	0.69	0.71	0.59	0.04
**11**	0.82	0.92	0.82	0.87	0.85	0.82	0.8	0.81	0.82	0.88	0.93	0.82	0.86	1	0.46	0.81	0.59	0.53	0.52	0.63	0.6	0.54	0.48	0.58	0.57	0.72	0.55	0.4	0.12
**12**	0.82	0.81	0.83	0.8	0.9	0.86	0.9	0.87	0.89	0.76	0.74	0.84	0.92	0.76	1	0.45	0.4	0.42	0.42	0.47	0.55	0.61	0.61	0.47	0.62	0.51	0.65	0.76	0.04
**15**	0.79	0.9	0.79	0.84	0.83	0.83	0.82	0.81	0.81	0.87	0.91	0.8	0.84	0.97	0.75	1	0.6	0.54	0.54	0.62	0.59	0.54	0.49	0.59	0.55	0.73	0.55	0.41	0.13
**16**	0.8	0.8	0.79	0.79	0.78	0.87	0.79	0.81	0.76	0.96	0.93	0.76	0.76	0.86	0.7	0.87	1	0.8	0.78	0.52	0.64	0.59	0.55	0.69	0.47	0.49	0.41	0.37	0.23
**18**	0.8	0.77	0.79	0.78	0.78	0.91	0.84	0.85	0.78	0.92	0.88	0.74	0.76	0.82	0.75	0.83	0.97	1	0.84	0.52	0.63	0.61	0.61	0.67	0.48	0.48	0.44	0.42	0.23
**19**	0.81	0.76	0.79	0.79	0.78	0.93	0.85	0.87	0.79	0.94	0.88	0.74	0.75	0.82	0.75	0.83	0.96	0.98	1	0.51	0.67	0.63	0.63	0.71	0.48	0.46	0.42	0.41	0.21
**20**	0.86	0.89	0.86	0.89	0.84	0.83	0.85	0.83	0.83	0.82	0.86	0.82	0.85	0.89	0.78	0.88	0.82	0.82	0.81	1	0.61	0.55	0.47	0.55	0.56	0.62	0.55	0.41	0.07
**21**	0.91	0.87	0.92	0.92	0.88	0.94	0.91	0.93	0.92	0.95	0.92	0.88	0.87	0.89	0.85	0.88	0.91	0.9	0.92	0.89	1	0.72	0.68	0.74	0.64	0.56	0.56	0.53	0.1
**22**	0.85	0.82	0.85	0.85	0.89	0.92	0.88	0.89	0.89	0.88	0.85	0.86	0.88	0.83	0.87	0.84	0.87	0.89	0.9	0.86	0.94	1	0.71	0.7	0.64	0.54	0.57	0.54	0.1
**23**	0.83	0.78	0.83	0.81	0.84	0.96	0.91	0.94	0.88	0.87	0.82	0.8	0.84	0.8	0.89	0.81	0.83	0.88	0.9	0.8	0.93	0.93	1	0.65	0.56	0.48	0.54	0.63	0.11
**24**	0.84	0.83	0.84	0.85	0.84	0.93	0.86	0.89	0.85	0.94	0.89	0.83	0.83	0.86	0.79	0.87	0.92	0.91	0.93	0.84	0.95	0.93	0.9	1	0.58	0.54	0.49	0.45	0.14
**25**	0.88	0.91	0.89	0.9	0.95	0.84	0.86	0.87	0.92	0.84	0.84	0.95	0.95	0.86	0.89	0.84	0.8	0.79	0.79	0.85	0.9	0.9	0.85	0.87	1	0.67	0.7	0.56	0.05
**26**	0.82	0.94	0.83	0.88	0.9	0.81	0.83	0.82	0.85	0.83	0.87	0.87	0.92	0.94	0.82	0.94	0.8	0.78	0.77	0.89	0.86	0.84	0.8	0.84	0.91	1	0.69	0.46	0.09
**27**	0.84	0.88	0.85	0.86	0.93	0.82	0.87	0.85	0.91	0.78	0.79	0.89	0.94	0.85	0.91	0.85	0.73	0.75	0.74	0.85	0.86	0.85	0.83	0.8	0.93	0.92	1	0.64	0.06
**28**	0.81	0.77	0.83	0.78	0.87	0.86	0.89	0.88	0.9	0.76	0.72	0.82	0.87	0.73	0.96	0.72	0.68	0.73	0.74	0.74	0.85	0.84	0.89	0.77	0.85	0.77	0.9	1	0.04
**29**	0.21	0.21	0.18	0.17	0.2	0.33	0.26	0.27	0.17	0.46	0.4	0.17	0.18	0.31	0.18	0.33	0.46	0.45	0.43	0.23	0.31	0.29	0.3	0.35	0.22	0.27	0.22	0.19	1

S2 Table 3: Algorithms from OpenModeler [92] with their parameter settings used for the prediction of Eco-Linguistic Niches of language groups.

**Algorithm name**	***OpenModeler* parameter settings**
Bioclim	Algorithm: BIOCLIM, StandardDeviationCutoff 0.2
NEW-GARP	Algorithm: GARP, MaxGenerations 400, ConvergenceLimit 0.01, PopulationSize 50, Resamples 2500
NEW-GARP-light	Algorithm: GARP, MaxGenerations 200, ConvergenceLimit 0.1, PopulationSize 20, Resamples 1000
NEW-GARP-BS	Algorithm: GARP-BS, TrainingProportion 50, TotalRuns 20, HardOmissionThreshold 100, ModelsUnderOmissionThreshold 20, CommissionThreshold 50, CommissionSampleSize 10000, MaxThreads 1, MaxGenerations 400, ConvergenceLimit 0.01, PopulationSize 50, Resamples 2500
NEW-GARP-BS-light	Algorithm: GARP-BS, TrainingProportion 50, TotalRuns 20, HardOmissionThreshold 100, ModelsUnderOmissionThreshold 20, CommissionThreshold 50, CommissionSampleSize 1000, MaxThreads 1, MaxGenerations 200, ConvergenceLimit 0.01, PopulationSize 50, Resamples 100
DESKTOP-GARP-BS	Algorithm: DG-GARP-BS, TrainingProportion 50, TotalRuns 20, HardOmissionThreshold 100, ModelsUnderOmissionThreshold 20, CommissionThreshold 50, CommissionSampleSize 10000, MaxThreads 1, MaxGenerations 400, ConvergenceLimit 0.01, PopulationSize 50, Resamples 2500
DESKTOP-GARP-BS-light	Algorithm: DG-GARP-BS, TrainingProportion 80, TotalRuns 20, HardOmissionThreshold 1, ModelsUnderOmissionThreshold 18, CommissionThreshold 30, CommissionSampleSize 100, MaxGenerations 200, ConvergenceLimit 0.05, PopulationSize 50, Resamples 50, MutationRate 0.25, CrossoverRate 0.25
Chebyshev-Environmental-distance	Algorithm: ENVDIST, DistanceType 4, NearestPoints 0, MaxDistance 0.1
SVM	Algorithm: SVM, SvmType 0, KernelType 2, Degree 3, Gamma 0, C 1, Coef0 0, Nu 0.5, ProbabilisticOutput 0, NumberOfPseudoAbsences 500
MAXENT	Algorithm: MAXENT, NumberOfBackgroundPoints 10000, UseAbsencesAsBackground 0, IncludePresencePointsInBackground 1, NumberOfIterations 500, TerminateTolerance 0.00001, OutputFormat 2, QuadraticFeatures 1, ProductFeatures 1, HingeFeatures 1, ThresholdFeatures 1, AutoFeatures 1, MinSamplesForProductThreshold 80, MinSamplesForQuadratic 10, MinSamplesForHinge 15
ANN	Algorithm: ANN, HiddenLayerNeurons 14, LearningRate 0.3, Momentum 0.05, Choice 1, Epoch 5000000, MinimunError 0.01
ANN-light	Algorithm: ANN, HiddenLayerNeurons 5, LearningRate 0.3, Momentum 0.05, Choice 1, Epoch 1000, MinimunError 0.01
EnvelopeScore	Algorithm: ENVSCORE
NicheMosaic	Algorithm: NICHE-MOSAIC, NumberOfIterations 2000
RandomForests	Algorithm: RF, NumTrees 20, VarsPerTree 3, ForceUnsupervisedLearning 0

S2 Table 4: Description of the consensus building method used for Eco-Linguistic Niches Modelling and obtained values for Accuracy (ACC), Area Under the "Receiver Operating Characteristics (ROC)" Curve (AUC) and Partial ROC ratio (P-ROC-ratio). A: Steps of the consensus building method. B: Distribution of the ACC and AUC values for the 15 modelling techniques (Table 2).C: ACC, AUC and P-ROC-ratio values for the Eco-Linguistic Niches of language groups according to modelling technique.

A.

Step 1: Selection Accuracy (ACC) is the proportion of occurrences correctly classified by the model (i.e predicted presence at presence location), see simple accuracy definition [93]. The Receiver Operating Characteristic Curve (ROC – [94]) reflects the fraction of true positives (sensitivity) depending on the fraction of false positives (1-specificity). The measurement of the Area Under this Curve (AUC) makes it possible to evaluate the performance of a predictive algorithm for a given dataset [95]. AUC ranges from 0 (null performance) to 1 (maximum performance). In the selection stage, we only selected the predictions with an ACC > 50 and an AUC ≥ 0.95.

Step 2: Weighting The partial ROC ratio calculates, at the very high sensitivity degree part of a prediction ROC curve, the difference in performance (AUC) between this curve and the ROC curve of a model that corresponds to random predictions [96]. This measure is considered as a good indicator to assess prediction reliability [97]. In order to take into account the performance of each predictive algorithm in the consensus prediction, predictions selected in the Step 1 (Selection) have been weighted by the value of their partial ROC ratio. In order to avoid distorting the mean highly discriminating certain predictions, the values of partial Roc less than 1 have been elevated to 1.

Step 3: Addition Finally, the predictions retained and weighted were summed, then normalized to obtain probabilities of presence between 0 and 1.

B.

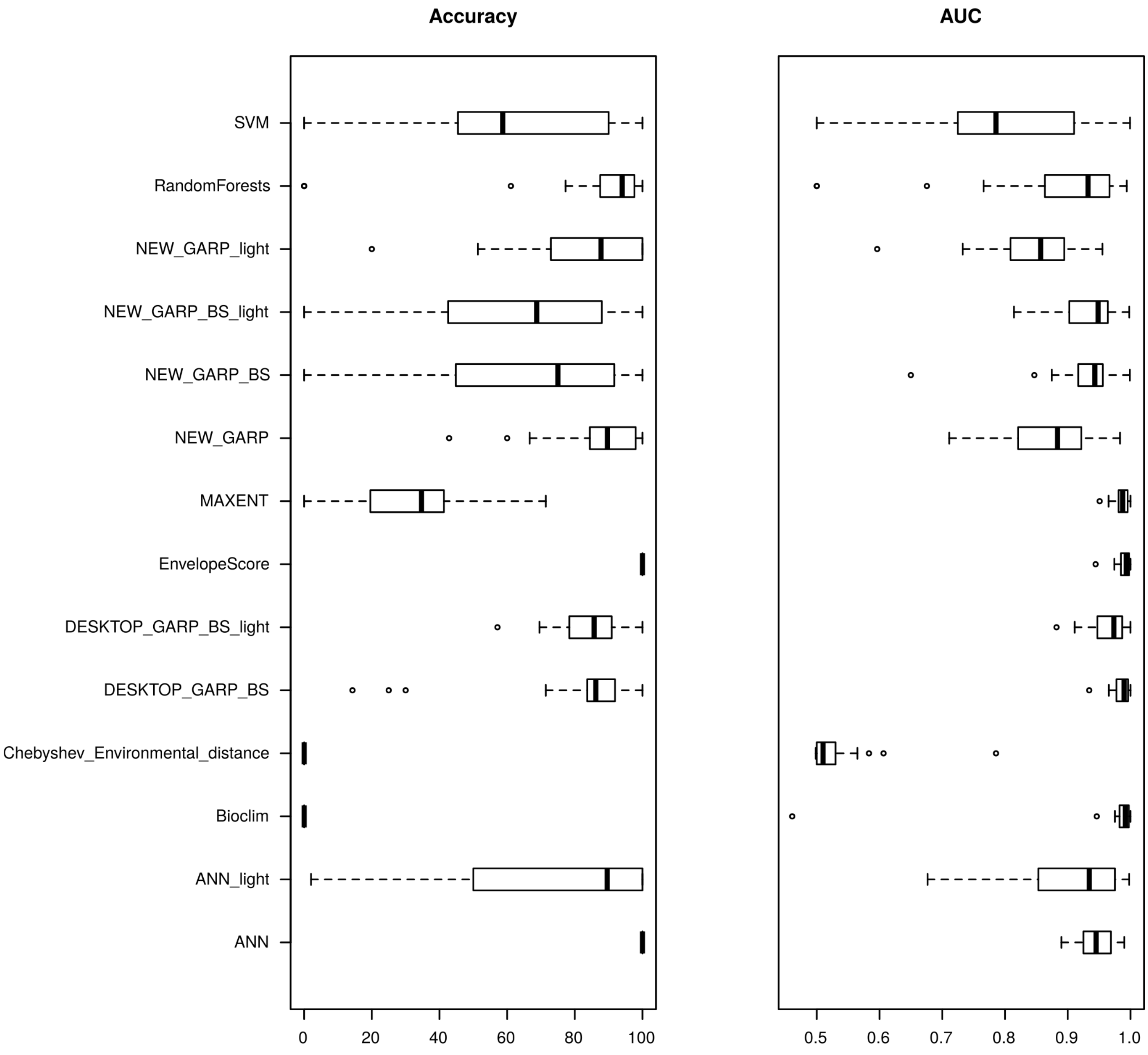
Accuracy
AUC
SVM
RandomForests
NEW_GARP_light
NEW_GARP_BS_light
NEW_GARP_BS
NEW_GARP
MAXENT
EnvelopeScore
DESKTOP_GARP_BS_light
DESKTOP_GARP_BS
Chebyshev_Environmental_distance
Bioclim
ANN_light
ANN
0
20
40
60
80
100
0.5
0.6
0.7
0.8
0.9
1.0

C.

Linguistic group	Algorithm	ACC	AUC	P-ROC-ratio
00-west-TNG	ANN	100	0.934	1.867
00-west-TNG	ANN-light	100	0.975	1.95
00-west-TNG	Bioclim	0	461	1
00-west-TNG	Chebyshev-Environmental-distance	0	0.5	1
00-west-TNG	DESKTOP-GARP-BS	25	0.999	1.998
00-west-TNG	DESKTOP-GARP-BS-light	100	0.968	1
00-west-TNG	EnvelopeScore	100	1	2
00-west-TNG	MAXENT	37.5	0.992	1.984
00-west-TNG	NEW-GARP	87.5	0.891	1
00-west-TNG	NEW-GARP-BS	75	0.98	1
00-west-TNG	NEW-GARP-BS-light	87.5	0.971	1
00-west-TNG	NEW-GARP-light	75	0.868	1
00-west-TNG	RandomForests	100	0.873	1.747
00-west-TNG	SVM	0	0.5	1
08-Mek	ANN	100	0.906	1.813
08-Mek	ANN-light	90.909	0.974	1.948
08-Mek	Bioclim	0	0.978	1
08-Mek	Chebyshev-Environmental-distance	0	0.523	1
08-Mek	DESKTOP-GARP-BS	72.727	0.989	1
08-Mek	DESKTOP-GARP-BS-light	90.909	0.945	1
08-Mek	EnvelopeScore	100	0.98	1.96
08-Mek	MAXENT	50	0.951	1.902
08-Mek	NEW-GARP	90.909	0.936	1
08-Mek	NEW-GARP-BS	86.364	0.942	1
08-Mek	NEW-GARP-BS-light	90.909	0.939	1
08-Mek	NEW-GARP-light	81.818	0.894	1
08-Mek	RandomForests	90.909	0.833	1.665
08-Mek	SVM	77.273	0.882	1
09-Asmat-Kamoro	ANN	100	0.916	1.831
09-Asmat-Kamoro	ANN-light	25	0.757	1.513
09-Asmat-Kamoro	Bioclim	0	0.979	1
09-Asmat-Kamoro	Chebyshev-Environmental-distance	0	0.53	1.06
09-Asmat-Kamoro	DESKTOP-GARP-BS	84.375	0.972	1
09-Asmat-Kamoro	DESKTOP-GARP-BS-light	81.25	0.952	1
09-Asmat-Kamoro	EnvelopeScore	100	0.98	1.959

Linguistic group	Algorithm	ACC	AUC	P-ROC-ratio
09-Asmat-Kamoro	MAXENT	46.875	0.985	1.969
09-Asmat-Kamoro	NEW-GARP	87.5	0.788	1
09-Asmat-Kamoro	NEW-GARP-BS	84.375	0.847	1
09-Asmat-Kamoro	NEW-GARP-BS-light	68.75	0.916	1
09-Asmat-Kamoro	NEW-GARP-light	100	0.749	1
09-Asmat-Kamoro	NicheMosaic	0	0.973	1
09-Asmat-Kamoro	RandomForests	84.375	0.953	1.906
09-Asmat-Kamoro	SVM	31.25	0.653	1
10-Awyu-Domot	ANN	100	0.893	1.786
10-Awyu-Domot	ANN-light	2.041	0.677	1.353
10-Awyu-Domot	Bioclim	0	0.981	1
10-Awyu-Domot	Chebyshev-Environmental-distance	0	0.51	1
10-Awyu-Domot	DESKTOP-GARP-BS	85.714	0.971	1
10-Awyu-Domot	DESKTOP-GARP-BS-light	75.51	0.952	1
10-Awyu-Domot	EnvelopeScore	100	0.982	1.963
10-Awyu-Domot	MAXENT	20.408	0.975	1.951
10-Awyu-Domot	NEW-GARP	71.429	0.824	1
10-Awyu-Domot	NEW-GARP-BS	87.755	0.928	1
10-Awyu-Domot	NEW-GARP-BS-light	61.224	0.896	1
10-Awyu-Domot	NEW-GARP-light	97.959	0.749	1
10-Awyu-Domot	NicheMosaic	0	0.954	1
10-Awyu-Domot	RandomForests	100	0.98	1.959
10-Awyu-Domot	SVM	0	0.5	1
11-Ok	ANN	100	0.948	1.895
11-Ok	ANN-light	94	0.953	1.905
11-Ok	Bioclim	0	0.975	1
11-Ok	Chebyshev-Environmental-distance	0	0.51	1
11-Ok	DESKTOP-GARP-BS	84	0.966	1
11-Ok	DESKTOP-GARP-BS-light	90	0.911	1
11-Ok	EnvelopeScore	100	0.974	1.948
11-Ok	MAXENT	36	0.971	1.942
11-Ok	NEW-GARP	92	0.884	1
11-Ok	NEW-GARP-BS	88	0.943	1
11-Ok	NEW-GARP-BS-light	86	0.949	1
11-Ok	NEW-GARP-light	90	0.912	1
11-Ok	NicheMosaic	0	0.973	1

Linguistic group	Algorithm	ACC	AUC	P-ROC-ratio
11-Ok	RandomForests	100	0.766	1.532
11-Ok	SVM	78	0.882	1
12-Marind	ANN	100	0.916	1.832
12-Marind	ANN-light	27.66	0.945	1.889
12-Marind	Bioclim	0	0.98	1
12-Marind	Chebyshev-Environmental-distance	0	0.499	1
12-Marind	DESKTOP-GARP-BS	91.489	0.965	1
12-Marind	DESKTOP-GARP-BS-light	82.979	0.958	1
12-Marind	EnvelopeScore	100	0.98	1.959
12-Marind	MAXENT	12.766	0.985	1.97
12-Marind	NEW-GARP	91.489	0.879	1
12-Marind	NEW-GARP-BS	46.809	0.875	1
12-Marind	NEW-GARP-BS-light	42.553	0.876	1
12-Marind	NEW-GARP-light	93.617	0.876	1
12-Marind	NicheMosaic	0	0.966	1
12-Marind	RandomForests	95.745	0.963	1.926
12-Marind	SVM	0	0.5	1
15-Bosavi	ANN	100	0.973	1.945
15-Bosavi	ANN-light	93.878	0.957	1.914
15-Bosavi	Bioclim	0	0.997	1
15-Bosavi	Chebyshev-Environmental-distance	0	0.51	1
15-Bosavi	DESKTOP-GARP-BS	91.837	0.994	1
15-Bosavi	DESKTOP-GARP-BS-light	79.592	0.995	1
15-Bosavi	EnvelopeScore	100	0.997	1.993
15-Bosavi	MAXENT	34.694	0.987	1.975
15-Bosavi	NEW-GARP	100	0.899	1
15-Bosavi	NEW-GARP-BS	97.959	0.954	1
15-Bosavi	NEW-GARP-BS-light	97.959	0.956	1
15-Bosavi	NEW-GARP-light	87.755	0.91	1
15-Bosavi	NicheMosaic	12.245	0.967	1
15-Bosavi	RandomForests	100	0.994	1.988
15-Bosavi	SVM	83.673	0.91	1
16-Engan	ANN	100	0.945	1.889
16-Engan	ANN-light	89.583	0.957	1.914
16-Engan	Bioclim	0	0.987	1
16-Engan	Chebyshev-Environmental-distance	0	0.51	1

Linguistic group	Algorithm	ACC	AUC	P-ROC-ratio
16-Engan	DESKTOP-GARP-BS	93.75	0.987	1
16-Engan	DESKTOP-GARP-BS-light	89.583	0.932	1
16-Engan	EnvelopeScore	100	0.986	1.972
16-Engan	MAXENT	31.25	0.971	1.941
16-Engan	NEW-GARP	89.583	0.908	1
16-Engan	NEW-GARP-BS	91.667	0.96	1
16-Engan	NEW-GARP-BS-light	75	0.949	1
16-Engan	NEW-GARP-light	72.917	0.857	1
16-Engan	NicheMosaic	0	0.972	1
16-Engan	RandomForests	93.75	0.932	1.865
16-Engan	SVM	72.917	0.852	1
18-Chimbu-Wahgi	ANN	100	0.979	1.959
18-Chimbu-Wahgi	ANN-light	59.184	0.896	1.793
18-Chimbu-Wahgi	Bioclim	0	0.989	1
18-Chimbu-Wahgi	Chebyshev-Environmental-distance	0	0.5	1
18-Chimbu-Wahgi	DESKTOP-GARP-BS	85.714	0.985	1
18-Chimbu-Wahgi	DESKTOP-GARP-BS-light	83.673	0.978	1
18-Chimbu-Wahgi	EnvelopeScore	100	0.989	1.978
18-Chimbu-Wahgi	MAXENT	34.694	0.995	1.991
18-Chimbu-Wahgi	NEW-GARP	100	0.946	1
18-Chimbu-Wahgi	NEW-GARP-BS	100	0.956	1
18-Chimbu-Wahgi	NEW-GARP-BS-light	97.959	0.952	1
18-Chimbu-Wahgi	NEW-GARP-light	100	0.92	1
18-Chimbu-Wahgi	NicheMosaic	0	0.949	1
18-Chimbu-Wahgi	RandomForests	95.918	0.967	1.933
18-Chimbu-Wahgi	SVM	55.102	0.766	1
19-Kainantu-Goroka	ANN	100	0.963	1.925
19-Kainantu-Goroka	ANN-light	92	0.982	1.964
19-Kainantu-Goroka	Bioclim	0	0.993	1
19-Kainantu-Goroka	Chebyshev-Environmental-distance	0	0.53	1.06
19-Kainantu-Goroka	DESKTOP-GARP-BS	96	0.994	1
19-Kainantu-Goroka	DESKTOP-GARP-BS-light	92	0.962	1
19-Kainantu-Goroka	EnvelopeScore	100	0.993	1.986
19-Kainantu-Goroka	MAXENT	20	0.985	1.97
19-Kainantu-Goroka	NEW-GARP	98	0.94	1
19-Kainantu-Goroka	NEW-GARP-BS	92	0.963	1

Linguistic group	Algorithm	ACC	AUC	P-ROC-ratio
19-Kainantu-Goroka	NEW-GARP-BS-light	88	0.975	1
19-Kainantu-Goroka	NEW-GARP-light	66	0.825	1
19-Kainantu-Goroka	NicheMosaic	0	0.977	1
19-Kainantu-Goroka	RandomForests	98	0.99	1.98
19-Kainantu-Goroka	SVM	90	0.937	1
20-Madang	ANN	100	0.943	1.886
20-Madang	ANN-light	69.565	0.953	1.906
20-Madang	Bioclim	0	0.991	1
20-Madang	Chebyshev-Environmental-distance	0	0.521	1
20-Madang	DESKTOP-GARP-BS	86.957	0.983	1
20-Madang	DESKTOP-GARP-BS-light	69.565	0.974	1
20-Madang	EnvelopeScore	100	0.991	1.981
20-Madang	MAXENT	19.565	0.993	1.986
20-Madang	NEW-GARP	100	0.833	1
20-Madang	NEW-GARP-BS	97.826	0.917	1
20-Madang	NEW-GARP-BS-light	95.652	0.966	1
20-Madang	NEW-GARP-light	93.478	0.864	1
20-Madang	NicheMosaic	0	0.958	1
20-Madang	RandomForests	100	0.962	1.925
20-Madang	SVM	58.696	0.786	1
21-Finisterre-Huon	ANN	100	0.933	1.867
21-Finisterre-Huon	ANN-light	62.222	0.846	1.692
21-Finisterre-Huon	Bioclim	0	0.992	1
21-Finisterre-Huon	Chebyshev-Environmental-distance	0	0.5	1
21-Finisterre-Huon	DESKTOP-GARP-BS	84.444	0.993	1
21-Finisterre-Huon	DESKTOP-GARP-BS-light	75.556	0.947	1
21-Finisterre-Huon	EnvelopeScore	100	0.992	1.984
21-Finisterre-Huon	MAXENT	37.778	0.997	1.993
21-Finisterre-Huon	NEW-GARP	84.444	0.897	1
21-Finisterre-Huon	NEW-GARP-BS	71.111	0.939	1
21-Finisterre-Huon	NEW-GARP-BS-light	51.111	0.962	1
21-Finisterre-Huon	NEW-GARP-light	77.778	0.877	1
21-Finisterre-Huon	NicheMosaic	0	0.979	1
21-Finisterre-Huon	RandomForests	95.556	0.864	1.727
21-Finisterre-Huon	SVM	51.111	0.751	1
22-Binanderean	ANN	100	0.89	1.779

Linguistic group	Algorithm	ACC	AUC	P-ROC-ratio
22-Binanderean	ANN-light	50	0.913	1.825
22-Binanderean	Bioclim	0	0.985	1
22-Binanderean	Chebyshev-Environmental-distance	0	0.5	1
22-Binanderean	DESKTOP-GARP-BS	87.5	0.975	1
22-Binanderean	DESKTOP-GARP-BS-light	75	0.938	1
22-Binanderean	EnvelopeScore	100	0.985	1.969
22-Binanderean	MAXENT	39.583	0.981	1.962
22-Binanderean	NEW-GARP	85.417	0.856	1
22-Binanderean	NEW-GARP-BS	50	0.939	1
22-Binanderean	NEW-GARP-BS-light	47.917	0.93	1
22-Binanderean	NEW-GARP-light	77.083	0.821	1
22-Binanderean	NicheMosaic	0	0.975	1
22-Binanderean	RandomForests	87.5	0.904	1.808
22-Binanderean	SVM	0	0.5	1
23-Southeast-Papuan	ANN	100	0.919	1.839
23-Southeast-Papuan	ANN-light	47.826	0.834	1.667
23-Southeast-Papuan	Bioclim	0	0.946	1
23-Southeast-Papuan	Chebyshev-Environmental-distance	0	0.5	1
23-Southeast-Papuan	DESKTOP-GARP-BS	89.13	0.934	1
23-Southeast-Papuan	DESKTOP-GARP-BS-light	80.435	0.937	1
23-Southeast-Papuan	EnvelopeScore	100	0.944	1.889
23-Southeast-Papuan	MAXENT	41.304	0.965	1.93
23-Southeast-Papuan	NEW-GARP	71.739	0.821	1
23-Southeast-Papuan	NEW-GARP-BS	58.696	0.906	1
23-Southeast-Papuan	NEW-GARP-BS-light	58.696	0.894	1
23-Southeast-Papuan	NEW-GARP-light	71.739	0.815	1
23-Southeast-Papuan	NicheMosaic	0	0.94	1
23-Southeast-Papuan	RandomForests	93.478	0.942	1.884
23-Southeast-Papuan	SVM	41.304	0.699	1
24-Angan	ANN	100	0.94	1.879
24-Angan	ANN-light	73.469	0.878	1.755
24-Angan	Bioclim	0	0.984	1
24-Angan	Chebyshev-Environmental-distance	0	0.5	1
24-Angan	DESKTOP-GARP-BS	83.673	0.978	1
24-Angan	DESKTOP-GARP-BS-light	87.755	0.882	1
24-Angan	EnvelopeScore	100	0.985	1.969

Linguistic group	Algorithm	ACC	AUC	P-ROC-ratio
24-Angan	MAXENT	30.612	0.989	1.979
24-Angan	NEW-GARP	77.551	0.859	1
24-Angan	NEW-GARP-BS	65.306	0.929	1
24-Angan	NEW-GARP-BS-light	73.469	0.9	1
24-Angan	NEW-GARP-light	69.388	0.819	1
24-Angan	NicheMosaic	2.041	0.95	1
24-Angan	RandomForests	97.959	0.91	1.819
24-Angan	SVM	57.143	0.773	1
25-Eleman	ANN	100	0.982	1.965
25-Eleman	ANN-light	35.135	0.934	1.868
25-Eleman	Bioclim	0	0.996	1
25-Eleman	Chebyshev-Environmental-distance	0	0.553	1.107
25-Eleman	DESKTOP-GARP-BS	91.892	0.989	1
25-Eleman	DESKTOP-GARP-BS-light	78.378	0.99	1
25-Eleman	EnvelopeScore	100	0.996	1.991
25-Eleman	MAXENT	51.351	0.984	1.967
25-Eleman	NEW-GARP	100	0.922	1
25-Eleman	NEW-GARP-BS	40.541	0.956	1
25-Eleman	NEW-GARP-BS-light	0	0.944	1.887
25-Eleman	NEW-GARP-light	51.351	0.753	1
25-Eleman	NicheMosaic	0	0.999	1
25-Eleman	RandomForests	94.595	0.968	1.936
25-Eleman	SVM	64.865	0.817	1
26-TuramKikorian	ANN	100	0.983	1.967
26-TuramKikorian	ANN-light	100	0.98	1.961
26-TuramKikorian	Bioclim	0	0.998	1
26-TuramKikorian	Chebyshev-Environmental-distance	0	0.55	1.1
26-TuramKikorian	DESKTOP-GARP-BS	98	0.996	1
26-TuramKikorian	DESKTOP-GARP-BS-light	90	0.984	1
26-TuramKikorian	EnvelopeScore	100	0.998	1.996
26-TuramKikorian	MAXENT	2	0.988	1.975
26-TuramKikorian	NEW-GARP	100	0.804	1
26-TuramKikorian	NEW-GARP-BS	100	0.954	1
26-TuramKikorian	NEW-GARP-BS-light	100	0.964	1
26-TuramKikorian	NEW-GARP-light	100	0.955	1
26-TuramKikorian	NicheMosaic	0	0.978	1

Linguistic group	Algorithm	ACC	AUC	P-ROC-ratio
26-TuramKikorian	RandomForests	94	0.972	1.943
26-TuramKikorian	SVM	92	0.951	1
27-Kiwai-Porome	ANN	100	0.967	1.935
27-Kiwai-Porome	ANN-light	94.444	0.985	1.971
27-Kiwai-Porome	Bioclim	0	0.994	1
27-Kiwai-Porome	Chebyshev-Environmental-distance	0	0.5	1
27-Kiwai-Porome	DESKTOP-GARP-BS	86.111	0.985	1
27-Kiwai-Porome	DESKTOP-GARP-BS-light	91.667	0.979	1
27-Kiwai-Porome	EnvelopeScore	100	0.995	1.99
27-Kiwai-Porome	MAXENT	33.333	0.984	1.968
27-Kiwai-Porome	NEW-GARP	86.111	0.818	1
27-Kiwai-Porome	NEW-GARP-BS	77.778	0.943	1
27-Kiwai-Porome	NEW-GARP-BS-light	77.778	0.934	1
27-Kiwai-Porome	NEW-GARP-light	86.111	0.733	1
27-Kiwai-Porome	NicheMosaic	0	0.899	1
27-Kiwai-Porome	RandomForests	94.444	0.973	1.946
27-Kiwai-Porome	SVM	55.556	0.775	1
28-GogodalSuki	ANN	100	0.961	1.921
28-GogodalSuki	ANN-light	44.444	0.842	1.684
28-GogodalSuki	Bioclim	0	0.997	1
28-GogodalSuki	Chebyshev-Environmental-distance	0	0.565	1.13
28-GogodalSuki	DESKTOP-GARP-BS	93.333	0.994	1
28-GogodalSuki	DESKTOP-GARP-BS-light	97.778	0.987	1
28-GogodalSuki	EnvelopeScore	100	0.997	1.994
28-GogodalSuki	MAXENT	20	0.996	1.992
28-GogodalSuki	NEW-GARP	97.778	0.95	1
28-GogodalSuki	NEW-GARP-BS	22.222	0.986	1
28-GogodalSuki	NEW-GARP-BS-light	37.778	0.98	1.961
28-GogodalSuki	NEW-GARP-light	100	0.844	1
28-GogodalSuki	NicheMosaic	0	0.988	1
28-GogodalSuki	RandomForests	95.556	0.972	1.943
28-GogodalSuki	SVM	55.556	0.775	1
29-Uhunduni	ANN	100	0.934	1.868
29-Uhunduni	ANN-light	100	0.853	1.706
29-Uhunduni	Bioclim	0	0.999	1
29-Uhunduni	Chebyshev-Environmental-distance	0	0.5	1

Linguistic group	Algorithm	ACC	AUC	P-ROC-ratio
29-Uhunduni	DESKTOP-GARP-BS	14.286	0.998	1
29-Uhunduni	DESKTOP-GARP-BS-light	57.143	0.992	1
29-Uhunduni	EnvelopeScore	100	0.999	1.998
29-Uhunduni	MAXENT	71.429	0.993	1.986
29-Uhunduni	NEW-GARP	42.857	0.711	1
29-Uhunduni	NEW-GARP-BS	42.857	0.884	1.768
29-Uhunduni	NEW-GARP-BS-light	28.571	0.814	1.628
29-Uhunduni	NEW-GARP-light	71.429	0.816	1
31-Biak	ANN	100	0.99	1.98
31-Biak	ANN-light	100	0.995	1.99
31-Biak	Bioclim	0	0.999	1
31-Biak	Chebyshev-Environmental-distance	0	0.606	1.213
31-Biak	DESKTOP-GARP-BS	100	0.999	1
31-Biak	DESKTOP-GARP-BS-light	100	0.981	1
31-Biak	EnvelopeScore	100	1	1.999
31-Biak	MAXENT	0	0.999	1.999
31-Biak	NEW-GARP	71.429	0.856	1
31-Biak	NEW-GARP-BS	28.571	0.892	1
31-Biak	NEW-GARP-BS-light	35.714	0.873	1
31-Biak	NEW-GARP-light	96.429	0.926	1
31-Biak	NicheMosaic	0	0.982	1
31-Biak	RandomForests	89.286	0.868	1.736
31-Biak	SVM	100	0.996	1
32-Manus	ANN	100	0.984	1.967
32-Manus	ANN-light	100	0.998	1.996
32-Manus	Bioclim	0	1	1
32-Manus	Chebyshev-Environmental-distance	0	0.583	1.166
32-Manus	DESKTOP-GARP-BS	100	1	1
32-Manus	DESKTOP-GARP-BS-light	100	0.999	1
32-Manus	EnvelopeScore	100	1	1.999
32-Manus	MAXENT	0	0.999	1.998
32-Manus	NEW-GARP	100	0.93	1
32-Manus	NEW-GARP-BS	100	0.932	1
32-Manus	NEW-GARP-BS-light	100	0.93	1
32-Manus	NEW-GARP-light	100	0.931	1
32-Manus	NicheMosaic	0	1	1

Linguistic group	Algorithm	ACC	AUC	P-ROC-ratio
32-Manus	RandomForests	61.111	0.675	1.351
32-Manus	SVM	77.778	0.888	1
33-New-Ireland	ANN	100	0.925	1.85
33-New-Ireland	ANN-light	100	0.934	1.867
33-New-Ireland	Bioclim	0	0.998	1
33-New-Ireland	Chebyshev-Environmental-distance	0	0.498	1
33-New-Ireland	DESKTOP-GARP-BS	30	0.996	1
33-New-Ireland	DESKTOP-GARP-BS-light	90	0.984	1
33-New-Ireland	EnvelopeScore	100	0.998	1.996
33-New-Ireland	MAXENT	50	0.981	1.962
33-New-Ireland	NEW-GARP	60	0.794	1
33-New-Ireland	NEW-GARP-BS	0	0.65	1.3
33-New-Ireland	NEW-GARP-BS-light	0	0.848	1
33-New-Ireland	NEW-GARP-light	20	0.596	1
33-New-Ireland	RandomForests	50	0.929	1.859
33-New-Ireland	SVM	0	0.5	1
34-New-Britain	ANN	100	0.945	1.89
34-New-Britain	ANN-light	48.485	0.785	1.57
34-New-Britain	Bioclim	0	0.983	1
34-New-Britain	Chebyshev-Environmental-distance	0	0.5	1
34-New-Britain	DESKTOP-GARP-BS	81.818	0.966	1
34-New-Britain	DESKTOP-GARP-BS-light	75.758	0.931	1
34-New-Britain	EnvelopeScore	100	0.983	1.966
34-New-Britain	MAXENT	48.485	0.991	1.981
34-New-Britain	NEW-GARP	66.667	0.802	1
34-New-Britain	NEW-GARP-BS	30.303	0.914	1
34-New-Britain	NEW-GARP-BS-light	39.394	0.902	1
34-New-Britain	NEW-GARP-light	63.636	0.771	1
34-New-Britain	NicheMosaic	0	0.959	1
34-New-Britain	RandomForests	81.818	0.845	1.69
34-New-Britain	SVM	45.455	0.725	1
35-Trobriand	ANN	100	0.92	1.839
35-Trobriand	ANN-light	100	0.83	1.659
35-Trobriand	Bioclim	0	1	1
35-Trobriand	Chebyshev-Environmental-distance	0	0.786	1
35-Trobriand	DESKTOP-GARP-BS	71.429	1	1

Linguistic group	Algorithm	ACC	AUC	P-ROC-ratio
35-Trobriand	DESKTOP-GARP-BS-light	85.714	1	1
35-Trobriand	EnvelopeScore	100	1	2
35-Trobriand	MAXENT	0	1	1
35-Trobriand	NEW-GARP	85.714	0.926	1
35-Trobriand	NEW-GARP-BS	42.857	0.999	1
35-Trobriand	NEW-GARP-BS-light	71.429	0.998	1
35-Trobriand	NEW-GARP-light	100	0.873	1
35-Trobriand	RandomForests	71.429	0.826	1.652
35-Trobriand	SVM	0	0.5	1
36-Mekeo	ANN	100	0.969	1.938
36-Mekeo	ANN-light	50	0.985	1.971
36-Mekeo	Bioclim	0	0.994	1
36-Mekeo	Chebyshev-Environmental-distance	0	0.51	1
36-Mekeo	DESKTOP-GARP-BS	93.75	0.993	1
36-Mekeo	DESKTOP-GARP-BS-light	72.917	0.99	1
36-Mekeo	EnvelopeScore	100	0.993	1.987
36-Mekeo	MAXENT	39.583	0.988	1.975
36-Mekeo	NEW-GARP	91.667	0.816	1
36-Mekeo	NEW-GARP-BS	89.583	0.962	1
36-Mekeo	NEW-GARP-BS-light	62.5	0.948	1
36-Mekeo	NEW-GARP-light	100	0.809	1
36-Mekeo	NicheMosaic	0	0.956	1
36-Mekeo	RandomForests	93.75	0.944	1.888
36-Mekeo	SVM	75	0.868	1
37-Roro	ANN	100	0.983	1.967
37-Roro	ANN-light	51.724	0.914	1.829
37-Roro	Bioclim	0	0.997	1
37-Roro	Chebyshev-Environmental-distance	0	0.5	1
37-Roro	DESKTOP-GARP-BS	86.207	0.995	1
37-Roro	DESKTOP-GARP-BS-light	96.552	0.987	1
37-Roro	EnvelopeScore	100	0.997	1.994
37-Roro	MAXENT	17.241	0.997	1.995
37-Roro	NEW-GARP	100	0.901	1
37-Roro	NEW-GARP-BS	100	0.945	1
37-Roro	NEW-GARP-BS-light	89.655	0.966	1
37-Roro	NEW-GARP-light	100	0.914	1

Linguistic group	Algorithm	ACC	AUC	P-ROC-rati
37-Roro	NicheMosaic	20.69	0.929	1
37-Roro	RandomForests	93.103	0.944	1.887
37-Roro	SVM	55.172	0.774	1
38-Motu	ANN	100	0.968	1.935
38-Motu	ANN-light	100	0.987	1.975
38-Motu	Bioclim	0	0.999	1
38-Motu	Chebyshev-Environmental-distance	0	0.512	1
38-Motu	DESKTOP-GARP-BS	88.095	0.998	1
38-Motu	DESKTOP-GARP-BS-light	85.714	0.976	1
38-Motu	EnvelopeScore	100	0.998	1.996
38-Motu	MAXENT	4.762	0.998	1.997
38-Motu	NEW-GARP	97.619	0.983	1
38-Motu	NEW-GARP-BS	54.762	0.946	1
38-Motu	NEW-GARP-BS-light	61.905	0.997	1
38-Motu	NEW-GARP-light	76.19	0.857	1
38-Motu	NicheMosaic	0	0.974	1
38-Motu	RandomForests	97.619	0.82	1.639
38-Motu	SVM	97.619	0.981	1
39-Papuan-ticluster	ANN	100	0.961	1.923
39-Papuan-ticluster	ANN-light	93.103	0.918	1.836
39-Papuan-ticluster	Bioclim	0	0.992	1
39-Papuan-ticluster	Chebyshev-Environmental-distance	0	0.5	1
39-Papuan-ticluster	DESKTOP-GARP-BS	82.759	0.99	1
39-Papuan-ticluster	DESKTOP-GARP-BS-light	82.759	0.962	1
39-Papuan-ticluster	EnvelopeScore	100	0.992	1.984
39-Papuan-ticluster	MAXENT	41.379	0.979	1.958
39-Papuan-ticluster	NEW-GARP	89.655	0.917	1
39-Papuan-ticluster	NEW-GARP-BS	44.828	0.95	1.901
39-Papuan-ticluster	NEW-GARP-BS-light	34.483	0.949	1
39-Papuan-ticluster	NEW-GARP-light	96.552	0.741	1
39-Papuan-ticluster	NicheMosaic	0	0.879	1
39-Papuan-ticluster	RandomForests	93.103	0.93	1.86
39-Papuan-ticluster	SVM	20.69	0.603	1

S2 Table 5: Phylolinguistic classification of Trans New Guinean language groups according to Wurm [1975] [41], Ross [2005] [44], Glottolog version 3.0 [53],Ethnologue (15ed) [52], and Muturzikin [2005] [98] respectively, as well number of constituent languages according to Glottolog version 3.0.

	Philolinguistic rank according to Wurm 1975				
**Linguistic group**	**Phylum**		**Super Stock**	**Stock**	**Families**
01_West_Bomberai	TNG	Main section		West Bomberai	
02_Ekari					
03_Moni					
04_Demal					
06_Dani	TNG	Main section		Dani (or Dani-Kwerba)	
07_Yali					
08_Mek	TNG	Sub-phyla			Goliath (Mek) sub-phylum-level
09_Asmat_Kamoro	TNG	Main section	C. and S. New-Guinea Kutubuan	Central and South New-Guinea	Asmat-Kamoro
10_Awyu_Domot	TNG	Main section	C. and S. New-Guinea Kutubuan	Central and South New-Guinea	Awyu-Dumut
11_Ok	TNG	Main section	C. and S. New-Guinea Kutubuan	Central and South New-Guinea	Ok
12_Marind	TNG	Main section	(no super stock)	Marind	
15_Bosavi	TNG	Main section	C. and S. New-Guinea Kutubuan	Central and South New-Guinea	Bosavi
16_Engan	TNG	Main section	(no super stock)	East New Guinea Highlands	West Central (Engan)
17_Kutubu	TNG	Main section	C. and S. New-Guinea Kutubuan	Kutubuan	
18_Chimbu_Wahgi	TNG	Main section	(no super stock)	East New Guinea Highlands	Central (Chimbu-Wahgi)
19_Kainantu_Goroka	TNG	Main section	(no super stock)	East New Guinea Highlands	Eastern (Kainantu) ; East Central (Gorokan)
20_Madang	TNG	Sub-phyla			Madang and Adelbert Range sub-phylum
21_Finisterre_Huon	TNG	Main section	Finisterre-Huon		
22_Binanderean	TNG	Main section	Eastern Part (not a super stock)	Binandere	
23_Southeast_Papuan	TNG				
24_Angan	TNG	Main section		Angan stock-level family	
25_Eleman	TNG				
26_TuramKikorian	TNG				
27_Kiwai_Porome	TNG				
28_GogodalSuki	TNG	Main section	(no super stock)	Gogodala-Suki	
29_Uhunduni	TNG				

	Philolinguistic rank according to Ross 2005			
**Linguistic group**	**Family**		**Sub-groups**	**Micro-groups**
01_West_Bomberai	TNG		West Trans New Guinea linkage	West Bomberai
02_Ekari	TNG			
03_Moni	TNG			
04_Demal	TNG			
06_Dani	TNG		West Trans New Guinea linkage	Dani
07_Yali	TNG			
08_Mek	TNG		Mek	
09_Asmat_Kamoro	TNG	Asmat		
10_Awyu_Domot	TNG		Awyu-Dumut	
11_Ok	TNG		Ok	
12_Marind	TNG		Marind	
15_Bosavi	TNG		Bosavi	
16_Engan	TNG		Engan	
17_Kutubu	TNG	(not a sub group)	West Kutubu ; East Kutubu	
18_Chimbu_Wahgi	TNG		Chimbu–Wahgi	
19_Kainantu_Goroka	TNG		Kainantu–Goroka	Gorokan ; Kainantu
20_Madang	TNG		Madang	
21_Finisterre_Huon	TNG		Finisterre-Huon	Finisterre ; Huon
22_Binanderean	TNG		Binanderea	
23_Southeast_Papuan	TNG		Southeast Papuan	Goilalan (?) ; Koiarian ; Kwalean ; Manubaran ; Yareban ; Mailuan ; Dagan
24_Angan	TNG		Angan	Angaataha ; Nuclear Angan
25_Eleman	TNG		Eleman	
26_TuramKikorian	TNG		Turama–Kikori	
27_Kiwai_Porome	TNG		Kiwai–Porome	
28_GogodalSuki	TNG		Gogodala–Suki	
29_Uhunduni	TNG	(isolates)	Uhunduni	

	Philolinguistic rank according to Glottolog 3.0					
**Linguistic group**	**Top-level Family (1st rank)**	**Subfamily (2nd rank)**	**Subfamily (3rd rank)**	**Subfamily (4th rank)**	**Language**	**N Languages**
00_West_TNG	na					22
01_West_Bomberai	West Bomberai					3
02_Ekari	Nuclear Trans New Guinea	Paniai Lakes			Ekari	1
03_Moni	Nuclear Trans New Guinea	Paniai Lakes			Moni	1
04_Demal					Damal	1
06_Dani	Nuclear Trans New Guinea	Dani				13
07_Yali	Nuclear Trans New Guinea	Dani	Ngalik-Nduga	Yalic		3
08_Mek	Nuclear Trans New Guinea	Mek				8
09_Asmat_Kamoro	Asmat-Awyu-Ok	Asmat-Kamoro				11
10_Awyu_Domot	Asmat-Awyu-Ok	Awyu-Ok	Greater-Awyu	Awyu-Dumut		18
11_Ok	Asmat-Awyu-Ok	Awyu-Ok	Ok-Oksapim	Ok		20
12_Marind	Anim	Marind-Boazi-Yaqai	Marindic		Marind	2
15_Bosavi	Bosavi					2
16_Engan	Nuclear Trans New Guinea	Engan-Kewa-Huli	Engan			6
18_Chimbu_Wahgi	Nuclear Trans New Guinea	Chimbu-Wahgi				17
19_Kainantu_Goroka	Nuclear Trans New Guinea	Kainantu-Goroka				28
20_Madang	Nuclear Trans New Guinea	Madang				107
21_Finisterre_Huon	Nuclear Trans New Guinea	Finisterre-Huon				61
22_Binanderean	Nuclear Trans New Guinea	Greater Binanderean	Binanderean			13
23_Southeast_Papuan	Koiarian+Yareban+Mailuan+Dagan					30
24_Angan	Angan					13
25_Eleman	Elemen					5
26_Turama_Kikorian	Turama-Kikori					4
27_Kiwai_Porome	Kiwain					6
28_GogodalSuki	Suki-Gogodala					4
29_Uhunduni	(isolates)					1

	Philolinguistic rank according to Ethnologue 15th					
**Linguistic group**						
01_West_Bomberai	Trans-New Guinea	Main Section	Central and Western	West Bomberai		
02_Ekari	Trans-New Guinea	Main Section	Central and Western	Wissel Lakes-Kemandoga	Ekari-Wolani-Moni	Ekari
03_Moni	Trans-New Guinea	Main Section	Central and Western	Wissel Lakes-Kemandoga	Ekari-Wolani-Moni	Moni
04_Demal	NA					
06_Dani	Trans-New Guinea	Main Section	Central and Western	Dani-Kwerba	Southern	Dani
07_Yali	Trans-New Guinea	Main Section	Central and Western	Dani-Kwerba	Southern	Ngalik-Nduga
08_Mek	Trans-New Guinea	Mek				
09_Asmat_Kamoro	Trans-New Guinea	Main Section	Central and Western	Central and South New Guinea-	Central and South New Guinea	Asmat-Kamoro
10_Awyu_Domot	Trans-New Guinea	Main Section	Central and Western	Central and South New Guinea-	Central and South New Guinea	Awyu-Dumut
11_Ok	Trans-New Guinea	Main Section	Central and Western	Central and South New Guinea-	Central and South New Guinea	Ok
12_Marind	Trans-New Guinea	Main Section	Central and Western	Marind		
15_Bosavi	Trans-New Guinea	Main Section	Central and Western	Central and South New Guinea-	Central and South New Guinea	Bosavi
16_Engan	Trans-New Guinea	Main Section	Central and Western	East New Guinea Highlands	West-Central	Enga
17_Kutubu						
18_Chimbu_Wahgi	Trans-New Guinea	Main Section	Central and Western	East New Guinea Highlands	Central	
19_Kainantu_Goroka						
20_Madang	Trans-New Guinea	Madang-Adelbert Range	Madang			
21_Finisterre_Huon	Trans-New Guinea	Main Section	Central and Western	Huon-Finisterre		
22_Binanderean	Trans-New Guinea	Main Section	Eastern	Binanderean		
23_Southeast_Papuan						
24_Angan	Trans-New Guinea	Main Section	Central and Western	Angan		
25_Eleman	Trans-New Guinea	Eleman				
26_Turama_Kikorian	Trans-New Guinea	Turama-Kikorian				
27_Kiwai_Porome	(NA) Trans-New Guinea	Trans-Fly-Bulaka River	Trans-Fly	(only Kiwaian)		
28_Gogodala_Suki	Trans-New Guinea	Main Section	Central and Western	Gogodala-Suki		
29_Uhunduni	Trans-New Guinea	Main Section	Central and Western	Wissel Lakes-Kemandoga	Uhunduni	

	Philolinguistic rank according to Muturzikin (2005-2011)	
**Linguistic group**	**Subgroup**	
01_West_Bomberai	Trans-New Guinea	
02_Ekari	Trans-New Guinea	
03_Moni	Trans-New Guinea	
04_Demal	Isolated (Damal)	
06_Dani	Trans-New Guinea	
07_Yali	Trans-New Guinea	
08_Mek	Trans-New Guinea	
09_Asmat_Kamoro	Trans-New Guinea	
10_Awyu_Domot	Trans-New Guinea	
11_Ok	Trans-New Guinea	
12_Marind	Trans-New Guinea	
15_Bosavi	Trans-New Guinea	
16_Engan	Trans-New Guinea	
17_Kutubu		W+E Trans-New Guinea
18_Chimbu_Wahgi	Trans-New Guinea	
19_Kainantu_Goroka	Trans-New Guinea	
20_Madang	Trans-New Guinea	
21_Finisterre_Huon	Trans-New Guinea	
22_Binanderean	Trans-New Guinea	
23_Southeast_Papuan	Trans-New Guinea	
24_Angan	Trans-New Guinea	
25_Eleman	Trans-New Guinea	
26_Turama_Kikorian	Trans-New Guinea	
27_Kiwai_Porome	Trans-New Guinea	
28_Gogodala_Suki	Trans-New Guinea	
29_Uhunduni	(isolates)	

S2 Table 6: Geographical coordinates of a random sample of 7-50 villages per modeled language group.

Sub group	id	Longitude	Latitude	Sub group	id	Longitude
Biak	31	135.6666	-0.7333	Bosavi	15	143.25
Biak	31	135.95	-1.0333	Bosavi	15	142.3666
Biak	31	135.5166	-0.7833	Bosavi	15	142.3166
Biak	31	135.5333	-0.8	Bosavi	15	142.3333
Biak	31	135.8	-0.9833	Bosavi	15	142.6
Biak	31	135.5166	-0.65	Bosavi	15	142.4333
Biak	31	135.6833	-0.7166	Bosavi	15	142.6666
Biak	31	135.7	-0.8	Bosavi	15	142.4666
Biak	31	135.8166	-0.8833	Bosavi	15	143.0166
Biak	31	135.95	-1.0833	Bosavi	15	142.9333
Biak	31	135.7333	-0.8166	Bosavi	15	142.9833
Biak	31	135.9666	-1.0166	Bosavi	15	142.3333
Biak	31	135.45	-0.6333	Bosavi	15	142.2833
Biak	31	135.9166	-0.9166	Bosavi	15	142.4666
Biak	31	135.5333	-0.8166	Bosavi	15	142.8666
Biak	31	135.6833	-0.8333	Bosavi	15	142.75
Biak	31	135.3833	-0.6833	Engan	16	143.9666
Biak	31	135.9	-1.1166	Engan	16	143.95
Biak	31	136	-1.1	Engan	16	142.8333
Biak	31	135.6666	-0.6833	Engan	16	143.8
Biak	31	135.9666	-1.1333	Engan	16	143.3166
Biak	31	135.7666	-0.9166	Engan	16	143.8666
Biak	31	135.7666	-0.8833	Engan	16	142.7666
Biak	31	136.05	-1.15	Engan	16	143.4333
Biak	31	135.8666	-0.8166	Engan	16	143.4833
Biak	31	136	-1.15	Engan	16	143.35
Biak	31	135.9	-0.8666	Engan	16	143.4833
Biak	31	136	-1	Engan	16	143.7
Biak	31	135.5166	-0.8	Engan	16	143.15
Biak	31	135.8	-0.9333	Engan	16	143.8833
Biak	31	135.45	-0.65	Engan	16	143.6
Biak	31	135.8333	-1.1	Engan	16	144.0166
Biak	31	135.7666	-0.7333	Engan	16	143.0833

Sub group	id	Longitude	Latitude	Sub group	id	Longitude
Biak	31	135.4166	-0.75	Engan	16	143.65
Biak	31	135.7166	-0.8166	Engan	16	142.8166
Biak	31	135.4666	-0.8166	Engan	16	143.6166
Biak	31	135.8	-1.1	Engan	16	143.6166
Biak	31	135.5	-0.8166	Engan	16	143.4666
Biak	31	135.8333	-1.0333	Engan	16	143.8666
Biak	31	135.6833	-0.7	Engan	16	143.25
Biak	31	135.5	-0.75	Engan	16	143.9833
Biak	31	135.7833	-0.9166	Engan	16	143.7
Biak	31	135.4833	-0.8	Engan	16	143.5666
Biak	31	135.4166	-0.7666	Engan	16	143.0833
Biak	31	135.6	-0.7333	Engan	16	143.3
Biak	31	135.6	-0.6833	Engan	16	143.9666
Biak	31	135.7833	-0.8666	Engan	16	143.6333
Biak	31	135.6666	-0.85	Engan	16	143.6
Biak	31	135.85	-1.0166	Engan	16	143.8666
Biak	31	135.4666	-0.7833	Engan	16	143.3666
Manus	32	147.2666	-2.0166	Engan	16	143.55
Manus	32	146.7333	-1.9833	Engan	16	143.65
Manus	32	146.6833	-1.95	Engan	16	143.7833
Manus	32	146.95	-1.95	Engan	16	143.85
Manus	32	147.0166	-2.0166	Engan	16	142.8833
Manus	32	146.9	-1.9666	Engan	16	143.6166
Manus	32	146.5666	-2	Engan	16	143.95
Manus	32	147.05	-1.9666	Engan	16	143.0166
Manus	32	146.9333	-1.9666	Engan	16	143.5833
Manus	32	147.0666	-2.15	Engan	16	144
Manus	32	147.075	-1.9666	Engan	16	143.55
Manus	32	146.5833	-1.9833	Engan	16	143.85
Manus	32	146.6166	-1.9833	Engan	16	143.2
Manus	32	146.6666	-2.1166	Engan	16	143.5166
Manus	32	146.5666	-2.1333	Engan	16	143.7666
Manus	32	146.75	-1.9833	Engan	16	143.5833
Manus	32	146.7	-2.1166	Chimbu Wahgi	18	144.75
Manus	32	146.6166	-1.95	Chimbu Wahgi	18	144.2833
Manus	32	146.6333	-1.95	Chimbu Wahgi	18	144.3166

Sub group	id	Longitude	Latitude	Sub group	id	Longitude
Manus	32	146.65	-1.95	Chimbu Wahgi	18	144.75
Manus	32	146.6166	-1.9333	Chimbu Wahgi	18	144.15
Manus	32	147.25	-2	Chimbu Wahgi	18	145.15
Manus	32	147.1666	-2.1333	Chimbu Wahgi	18	144.5666
Manus	32	146.6666	-2.15	Chimbu Wahgi	18	144.7833
Manus	32	146.5166	-2.1833	Chimbu Wahgi	18	145.0833
Manus	32	147.0333	-1.9666	Chimbu Wahgi	18	144.8333
Manus	32	147.0333	-1.9833	Chimbu Wahgi	18	144.0833
Manus	32	146.5666	-2.0833	Chimbu Wahgi	18	144.4833
Manus	32	147.0666	-2.0333	Chimbu Wahgi	18	144
Manus	32	146.5166	-2.15	Chimbu Wahgi	18	143.9833
Manus	32	147.35	-2.0166	Chimbu Wahgi	18	144.8333
Manus	32	147.15	-2	Chimbu Wahgi	18	145.0166
Manus	32	146.6833	-2.1333	Chimbu Wahgi	18	144.95
Manus	32	147.1833	-2.0166	Chimbu Wahgi	18	145.0666
Manus	32	146.55	-2.1166	Chimbu Wahgi	18	144.9666
Manus	32	146.8	-1.9666	Chimbu Wahgi	18	144.85
Manus	32	146.55	-2.1	Chimbu Wahgi	18	145.05
Manus	32	146.6	-2	Chimbu Wahgi	18	145.1333
Manus	32	147.1666	-2.0666	Chimbu Wahgi	18	145.1
Manus	32	147.0666	-2.1333	Chimbu Wahgi	18	144.9333
Manus	32	146.7833	-1.9833	Chimbu Wahgi	18	145.05
Manus	32	146.5166	-2.1666	Chimbu Wahgi	18	144.9
Manus	32	146.7666	-1.9833	Chimbu Wahgi	18	144.4166
Manus	32	146.95	-2.1333	Chimbu Wahgi	18	144
Manus	32	147	-1.9666	Chimbu Wahgi	18	144.4833
Manus	32	147.2	-1.9833	Chimbu Wahgi	18	144.8666
Manus	32	147.0333	-2.1166	Chimbu Wahgi	18	144.9333
Manus	32	147.3166	-2.0166	Chimbu Wahgi	18	143.9833
Manus	32	146.5666	-1.9833	Chimbu Wahgi	18	144.8666
Manus	32	147.1833	-2.1	Chimbu Wahgi	18	144.8333
New Ireland	33	151.5333	-3	Chimbu Wahgi	18	144.8166
New Ireland	33	153.0333	-4.5166	Chimbu Wahgi	18	144.6
New Ireland	33	150.9	-2.7	Chimbu Wahgi	18	145.0666
New Ireland	33	153.0666	-4.6	Chimbu Wahgi	18	144.9
New Ireland	33	152.7	-4.4333	Chimbu Wahgi	18	144.9833

Sub group	id	Longitude	Latitude	Sub group	id	Longitude
New Ireland	33	151.1166	-2.7833	Chimbu Wahgi	18	145.05
New Ireland	33	151.3666	-2.9	Chimbu Wahgi	18	143.9333
New Ireland	33	152.5666	-3.8	Chimbu Wahgi	18	144.3
New Ireland	33	151.5	-3	Chimbu Wahgi	18	144.85
New Ireland	33	151.8833	-3.2	Chimbu Wahgi	18	144.0333
New Ireland	33	151.7666	-3.1333	Chimbu Wahgi	18	145.0166
New Ireland	33	151.5833	-3.0333	Chimbu Wahgi	18	144.9333
New Ireland	33	151.5333	-2.9833	Chimbu Wahgi	18	144.7
New Ireland	33	151.5166	-2.9833	Chimbu Wahgi	18	144.4333
New Ireland	33	151.75	-3.1333	Chimbu Wahgi	18	144.0833
New Ireland	33	152.5166	-3.7166	Chimbu Wahgi	18	144.5833
New Ireland	33	151.0333	-2.7	Kainantu Goroka	19	145.7
New Ireland	33	153.05	-4.2166	Kainantu Goroka	19	145.85
New Ireland	33	152.5166	-3.6833	Kainantu Goroka	19	145.4833
New Ireland	33	151.1833	-2.8	Kainantu Goroka	19	145.7166
New Ireland	33	152.95	-4.0166	Kainantu Goroka	19	145.65
New Ireland	33	152.65	-3.85	Kainantu Goroka	19	145.2666
New Ireland	33	151.6666	-3.05	Kainantu Goroka	19	145.45
New Ireland	33	153.0166	-4.6666	Kainantu Goroka	19	145.7166
New Ireland	33	152.6	-3.8333	Kainantu Goroka	19	145.4333
New Ireland	33	151.95	-3.25	Kainantu Goroka	19	144.9833
New Ireland	33	152.95	-4.75	Kainantu Goroka	19	145.7
New Ireland	33	151.3166	-2.8666	Kainantu Goroka	19	145.4
New Ireland	33	153.0666	-4.4166	Kainantu Goroka	19	145.6
New Ireland	33	151.9166	-3.2	Kainantu Goroka	19	145.9
New Ireland	33	152.1666	-3.4166	Kainantu Goroka	19	145.2333
New Ireland	33	152.2666	-3.4833	Kainantu Goroka	19	145.9333
New Ireland	33	150.9666	-2.6833	Kainantu Goroka	19	145.2666
New Ireland	33	151.7666	-3.15	Kainantu Goroka	19	145.9166
New Ireland	33	152.1333	-3.3666	Kainantu Goroka	19	145.6
New Ireland	33	151.7666	-3.1666	Kainantu Goroka	19	145.6333
New Ireland	33	151.4166	-2.9	Kainantu Goroka	19	145.2833
New Ireland	33	151.1	-2.7666	Kainantu Goroka	19	145.2
New Ireland	33	152.55	-3.75	Kainantu Goroka	19	145.1666
New Ireland	33	152.9333	-4.7666	Kainantu Goroka	19	145.9166
New Ireland	33	153.1333	-4.3166	Kainantu Goroka	19	145.65

Sub group	id	Longitude	Latitude	Sub group	id	Longitude
New Ireland	33	153.0333	-4.45	Kainantu Goroka	19	145.4666
New Ireland	33	151.1166	-2.8166	Kainantu Goroka	19	146.0166
New Ireland	33	151.0833	-2.75	Kainantu Goroka	19	145.7666
New Ireland	33	152.4166	-3.6833	Kainantu Goroka	19	145.4333
New Ireland	33	151.9666	-3.3	Kainantu Goroka	19	145.55
New Ireland	33	153.0166	-4.6333	Kainantu Goroka	19	145.6166
New Ireland	33	151.1333	-2.7833	Kainantu Goroka	19	145.7833
New Ireland	33	153.0166	-4.1166	Kainantu Goroka	19	145.55
New Ireland	33	152.5333	-3.7833	Kainantu Goroka	19	146.05
New Britain	34	148.75	-5.5166	Kainantu Goroka	19	145.6333
New Britain	34	152.0833	-5.2666	Kainantu Goroka	19	145.05
New Britain	34	151.8666	-5.5333	Kainantu Goroka	19	145.5833
New Britain	34	151.5333	-4.1833	Kainantu Goroka	19	145
New Britain	34	150.7	-5.6	Kainantu Goroka	19	145.85
New Britain	34	151.05	-5.9833	Kainantu Goroka	19	145.5666
New Britain	34	151.7833	-4.2166	Kainantu Goroka	19	145.5
New Britain	34	149	-5.7833	Kainantu Goroka	19	146
New Britain	34	150.0666	-5.6333	Kainantu Goroka	19	146.0833
New Britain	34	150.0333	-5.0833	Kainantu Goroka	19	145.7333
New Britain	34	150.1	-5.1	Kainantu Goroka	19	145.4833
New Britain	34	150.1833	-5.6166	Kainantu Goroka	19	146.0166
New Britain	34	151.8333	-4.9166	Kainantu Goroka	19	145.4
New Britain	34	150.0166	-5.6	Kainantu Goroka	19	145.5833
New Britain	34	148.8833	-5.8166	Kainantu Goroka	19	145.3166
New Britain	34	151.5833	-4.2	Kainantu Goroka	19	145.4333
New Britain	34	151.4333	-5.6166	Madang	20	145.9333
New Britain	34	150.3166	-6.2333	Madang	20	145.4833
New Britain	34	151.7	-5.3166	Madang	20	145.2666
New Britain	34	152.1666	-4.8166	Madang	20	145.65
New Britain	34	151.5166	-5.1833	Madang	20	144.9333
New Britain	34	150.2166	-5.6333	Madang	20	145.6833
New Britain	34	149.5833	-5.6166	Madang	20	145.2666
New Britain	34	149.1333	-5.6833	Madang	20	145.2333
New Britain	34	151.6666	-5.35	Madang	20	145.6166
New Britain	34	149.1833	-6.1	Madang	20	145.7
New Britain	34	149.7833	-5.5166	Madang	20	145.9833

Sub group	id	Longitude	Latitude	Sub group	id	Longitude
New Britain	34	149.25	-6.0666	Madang	20	145.5666
New Britain	34	151.5166	-4.2	Madang	20	145.5166
New Britain	34	152.35	-4.3666	Madang	20	145.2666
New Britain	34	148.3666	-5.6	Madang	20	144.3833
New Britain	34	152.15	-4.2166	Madang	20	145.2
New Britain	34	151.05	-5.2666	Madang	20	145.0666
New Britain	34	150.3333	-5.55	Madang	20	145.5333
New Britain	34	150.3666	-5.6333	Madang	20	145.6166
New Britain	34	150.1	-5.1166	Madang	20	144.85
New Britain	34	152.0833	-4.4166	Madang	20	145.4166
New Britain	34	150.2	-5.0833	Madang	20	145.4
New Britain	34	150.0833	-5.2666	Madang	20	145.7
New Britain	34	149.55	-6.2333	Madang	20	145.3333
New Britain	34	152.2333	-4.5833	Madang	20	145.6166
New Britain	34	152.3	-4.3833	Madang	20	145.0666
New Britain	34	151.2833	-5.8666	Madang	20	145.6333
New Britain	34	148.8	-5.5333	Madang	20	145.7
New Britain	34	149.9	-6.2666	Madang	20	145.5666
New Britain	34	152.2833	-4.4333	Madang	20	144.85
New Britain	34	148.6333	-5.4833	Madang	20	145.0666
New Britain	34	150.05	-5.3333	Madang	20	144.6333
New Britain	34	151.0833	-5.75	Madang	20	145.5666
New Britain	34	152.2166	-4.7833	Madang	20	145.9666
Trobriand	35	151.1	-8.8	Madang	20	145.6833
Trobriand	35	151.15	-8.5833	Madang	20	144.7666
Trobriand	35	151.05	-8.5166	Madang	20	145.4833
Trobriand	35	151.1333	-8.6	Madang	20	145.4833
Trobriand	35	151.1	-8.5	Madang	20	145.75
Trobriand	35	151.0666	-8.5333	Madang	20	145.35
Trobriand	35	151.1166	-8.5833	Madang	20	144.9333
Trobriand	35	151.1166	-8.8	Madang	20	144.9333
Trobriand	35	151.1	-8.5833	Madang	20	145.0666
Trobriand	35	151.1166	-8.5166	Madang	20	145.45
Trobriand	35	151.0333	-8.55	Madang	20	145.55
Trobriand	35	151.1	-8.5166	Madang	20	145.6666
Trobriand	35	151.0166	-8.55	Madang	20	144.95

Sub group	id	Longitude	Latitude	Sub group	id	Longitude
Trobriand	35	151.0166	-8.5333	Madang	20	145.75
Trobriand	35	151.0833	-8.55	Madang	20	144.2833
Trobriand	35	151.0666	-8.5	Madang	20	145.25
Mekeo	36	146.65	-8.4	Finisterre Huon	21	146.5833
Mekeo	36	146.5833	-8.5333	Finisterre Huon	21	146.5166
Mekeo	36	146.6666	-8.5	Finisterre Huon	21	147.05
Mekeo	36	146.45	-8.6166	Finisterre Huon	21	147.4166
Mekeo	36	146.6833	-8.2	Finisterre Huon	21	146.4
Mekeo	36	146.5	-8.3833	Finisterre Huon	21	145.8166
Mekeo	36	146.3666	-8.4	Finisterre Huon	21	147.5833
Mekeo	36	146.5333	-7.75	Finisterre Huon	21	146.3166
Mekeo	36	146.7	-7.7833	Finisterre Huon	21	146.6
Mekeo	36	146.3666	-8.55	Finisterre Huon	21	146.4166
Mekeo	36	146.5	-7.9	Finisterre Huon	21	147.3166
Mekeo	36	146.4666	-8.0166	Finisterre Huon	21	146.8333
Mekeo	36	146.6333	-8.2333	Finisterre Huon	21	146.6166
Mekeo	36	146.5666	-8.55	Finisterre Huon	21	147.65
Mekeo	36	146.5	-8.35	Finisterre Huon	21	146.2
Mekeo	36	146.4666	-8.4	Finisterre Huon	21	147.6
Mekeo	36	146.5666	-7.65	Finisterre Huon	21	146.3
Mekeo	36	146.4333	-7.9	Finisterre Huon	21	146.7
Mekeo	36	146.5666	-7.6333	Finisterre Huon	21	147.1666
Mekeo	36	146.5666	-7.6	Finisterre Huon	21	145.8166
Mekeo	36	146.4333	-8.6166	Finisterre Huon	21	147.5666
Mekeo	36	146.75	-7.9166	Finisterre Huon	21	146.15
Mekeo	36	146.55	-8.5666	Finisterre Huon	21	147.7
Mekeo	36	146.6166	-8.2333	Finisterre Huon	21	146.5
Mekeo	36	146.6666	-7.75	Finisterre Huon	21	146.1166
Mekeo	36	146.3833	-8.5833	Finisterre Huon	21	147.5833
Mekeo	36	146.55	-8.3333	Finisterre Huon	21	146.2
Mekeo	36	146.5666	-8.3166	Finisterre Huon	21	147.4333
Mekeo	36	146.6166	-8.15	Finisterre Huon	21	147.2166
Mekeo	36	146.6166	-8.5333	Finisterre Huon	21	146.45
Mekeo	36	146.4666	-8.6333	Finisterre Huon	21	147.3
Mekeo	36	146.6666	-7.7333	Finisterre Huon	21	146.3333
Mekeo	36	146.4166	-8.6	Finisterre Huon	21	146.6166

Sub group	id	Longitude	Latitude	Sub group	id	Longitude
Mekeo	36	146.6333	-8.25	Finisterre Huon	21	147.8333
Mekeo	36	146.6166	-7.7333	Finisterre Huon	21	147.1
Mekeo	36	146.4833	-8.4	Finisterre Huon	21	147.45
Mekeo	36	146.4	-8.6	Finisterre Huon	21	147.5833
Mekeo	36	146.5166	-7.85	Finisterre Huon	21	147.0833
Mekeo	36	146.45	-8.6333	Finisterre Huon	21	146.5333
Mekeo	36	146.6333	-8.45	Finisterre Huon	21	146.4833
Mekeo	36	146.4666	-8.5166	Finisterre Huon	21	147.4333
Mekeo	36	146.55	-7.65	Finisterre Huon	21	147.2833
Mekeo	36	146.7666	-8.0333	Finisterre Huon	21	147.1666
Mekeo	36	146.7333	-8.1	Finisterre Huon	21	147.2666
Mekeo	36	146.4333	-8	Finisterre Huon	21	147.7166
Mekeo	36	146.6	-8.5	Finisterre Huon	21	146.9666
Mekeo	36	146.45	-8.0666	Finisterre Huon	21	146.9166
Mekeo	36	146.6666	-8.1666	Finisterre Huon	21	147.2833
Mekeo	36	146.5666	-8.5666	Finisterre Huon	21	146.2
Mekeo	36	146.6166	-8.5	Finisterre Huon	21	146.9
Roro	37	146.7666	-8.6833	Binanderean	22	148.4666
Roro	37	146.5666	-8.95	Binanderean	22	147.85
Roro	37	147.0166	-9.05	Binanderean	22	148.1833
Roro	37	146.7166	-8.65	Binanderean	22	147.3
Roro	37	146.5333	-8.8166	Binanderean	22	147.1
Roro	37	146.85	-9.0166	Binanderean	22	149.2833
Roro	37	146.6666	-9.0333	Binanderean	22	147.2166
Roro	37	146.9333	-8.5166	Binanderean	22	149
Roro	37	146.8833	-8.5666	Binanderean	22	146.9166
Roro	37	146.9666	-8.9833	Binanderean	22	147.35
Roro	37	146.6	-8.5	Binanderean	22	147.8666
Roro	37	146.5833	-8.6166	Binanderean	22	147.1833
Roro	37	146.5666	-8.5666	Binanderean	22	148.2666
Roro	37	146.9166	-9	Binanderean	22	147.4333
Roro	37	146.4666	-8.6333	Binanderean	22	147.55
Roro	37	146.4	-8.6	Binanderean	22	148.2166
Roro	37	146.5833	-8.9666	Binanderean	22	148.45
Roro	37	147.0166	-8.5833	Binanderean	22	148.45
Roro	37	146.9	-9.1166	Binanderean	22	147.0666

Sub group	id	Longitude	Latitude	Sub group	id	Longitude
Roro	37	146.6833	-9	Binanderean	22	148.4666
Roro	37	146.6	-8.9666	Binanderean	22	147.3666
Roro	37	146.5333	-8.7166	Binanderean	22	148.25
Roro	37	146.6166	-8.7333	Binanderean	22	148.3
Roro	37	147.0333	-8.65	Binanderean	22	148.5
Roro	37	146.9666	-9.0333	Binanderean	22	148.0666
Roro	37	146.5666	-8.8666	Binanderean	22	148.05
Roro	37	146.5833	-8.9833	Binanderean	22	148.4333
Roro	37	146.45	-8.6333	Binanderean	22	147.5166
Roro	37	146.4666	-8.5166	Binanderean	22	148.6833
Roro	37	146.9166	-9.1166	Binanderean	22	148.1333
Roro	37	146.55	-8.8333	Binanderean	22	147.3333
Roro	37	146.6166	-9.0166	Binanderean	22	147.8166
Roro	37	146.9166	-8.5833	Binanderean	22	147.2166
Roro	37	146.6166	-8.5333	Binanderean	22	147.3833
Roro	37	146.95	-9.0833	Binanderean	22	146.95
Roro	37	146.6	-8.9166	Binanderean	22	148.0166
Roro	37	146.7833	-8.8166	Binanderean	22	148.6
Roro	37	146.5833	-8.5333	Binanderean	22	148.0833
Roro	37	146.5666	-8.85	Binanderean	22	146.9833
Roro	37	146.7	-8.6666	Binanderean	22	148.3333
Roro	37	147.0333	-8.9833	Binanderean	22	149.3
Roro	37	146.9833	-8.5666	Binanderean	22	147.85
Roro	37	146.7666	-9.0666	Binanderean	22	148.0166
Roro	37	146.5333	-8.8333	Binanderean	22	147.0666
Roro	37	146.9	-8.5666	Binanderean	22	147.9833
Roro	37	146.55	-8.7833	Binanderean	22	148.1166
Roro	37	146.5666	-8.55	Binanderean	22	147.9833
Roro	37	146.9666	-9.0166	Binanderean	22	148.7666
Roro	37	146.55	-8.5666	Binanderean	22	149.05
Roro	37	146.6	-8.7666	Binanderean	22	148.2833
Motu	38	147.9666	-9.75	Southeast Papuan	23	148.3333
Motu	38	148.1166	-10.1333	Southeast Papuan	23	147.6
Motu	38	147.5833	-9.8	Southeast Papuan	23	146.8
Motu	38	148.2	-10.05	Southeast Papuan	23	147.2833
Motu	38	148.2166	-10.1166	Southeast Papuan	23	147.25

Sub group	id	Longitude	Latitude	Sub group	id	Longitude
Motu	38	147.9666	-10.1666	Southeast Papuan	23	147.5
Motu	38	148	-9.9333	Southeast Papuan	23	146.6833
Motu	38	147.9166	-9.9166	Southeast Papuan	23	146.9666
Motu	38	147.7166	-10.1	Southeast Papuan	23	149.4666
Motu	38	147.7833	-9.5833	Southeast Papuan	23	147.1333
Motu	38	148	-9.7	Southeast Papuan	23	148.1666
Motu	38	147.5833	-9.7333	Southeast Papuan	23	147.6666
Motu	38	148.0666	-10.1	Southeast Papuan	23	147.2333
Motu	38	147.9666	-9.8	Southeast Papuan	23	147.05
Motu	38	147.7166	-9.6666	Southeast Papuan	23	147.1666
Motu	38	147.9666	-9.6833	Southeast Papuan	23	149.7166
Motu	38	147.8166	-9.6833	Southeast Papuan	23	147.3833
Motu	38	147.7666	-9.5833	Southeast Papuan	23	147.65
Motu	38	147.6166	-9.7166	Southeast Papuan	23	148.4333
Motu	38	147.7166	-9.8333	Southeast Papuan	23	148.5666
Motu	38	147.8833	-9.8833	Southeast Papuan	23	147.7
Motu	38	148.0333	-9.9333	Southeast Papuan	23	146.8333
Motu	38	147.95	-9.8166	Southeast Papuan	23	148.4166
Motu	38	147.7833	-9.65	Southeast Papuan	23	147.7666
Motu	38	148.1166	-9.7666	Southeast Papuan	23	147.6333
Motu	38	148.0833	-9.7166	Southeast Papuan	23	148.2
Motu	38	147.6666	-9.9666	Southeast Papuan	23	147.2666
Motu	38	147.4666	-9.7	Southeast Papuan	23	148.55
Motu	38	147.6	-9.8	Southeast Papuan	23	147.05
Motu	38	147.5666	-9.8	Southeast Papuan	23	148.7166
Motu	38	148	-9.9666	Southeast Papuan	23	149.2833
Motu	38	148.0833	-10.1	Southeast Papuan	23	146.7833
Motu	38	147.6666	-9.65	Southeast Papuan	23	149.5166
Motu	38	147.5666	-9.6833	Southeast Papuan	23	149.55
Motu	38	147.75	-9.6	Southeast Papuan	23	147.5833
Motu	38	147.9	-9.6666	Southeast Papuan	23	147.15
Motu	38	147.7166	-10.05	Southeast Papuan	23	148.4666
Motu	38	148.1833	-10.05	Southeast Papuan	23	149.5666
Motu	38	147.85	-9.7333	Southeast Papuan	23	146.7166
Motu	38	147.5	-9.8	Southeast Papuan	23	149.6166
Motu	38	147.65	-9.9166	Southeast Papuan	23	147.5666

Sub group	id	Longitude	Latitude	Sub group	id	Longitude
Motu	38	147.95	-9.9	Southeast Papuan	23	147.3333
Motu	38	147.6	-9.7833	Southeast Papuan	23	147.6
Motu	38	147.6666	-9.6333	Southeast Papuan	23	149.2
Motu	38	147.7666	-9.95	Southeast Papuan	23	148.3333
Motu	38	147.9666	-9.7	Southeast Papuan	23	147.6833
Motu	38	147.6	-9.8333	Southeast Papuan	23	148.3666
Motu	38	148.05	-9.7833	Southeast Papuan	23	149.5166
Motu	38	147.8666	-10.0666	Southeast Papuan	23	146.95
Motu	38	147.75	-9.6333	Southeast Papuan	23	147.4166
Papuan Tip Cluster	39	150.4	-10.35	Angan	24	146.45
Papuan Tip Cluster	39	150.2666	-10.4666	Angan	24	146.55
Papuan Tip Cluster	39	150.8833	-9.85	Angan	24	145.8333
Papuan Tip Cluster	39	150.25	-9.45	Angan	24	145.7
Papuan Tip Cluster	39	149.9666	-10.3833	Angan	24	146.0333
Papuan Tip Cluster	39	150.5	-10.3333	Angan	24	146.05
Papuan Tip Cluster	39	149.9833	-10.6	Angan	24	146.25
Papuan Tip Cluster	39	149.8666	-10.1	Angan	24	145.8833
Papuan Tip Cluster	39	150.4333	-10.6833	Angan	24	146.0166
Papuan Tip Cluster	39	150.3	-9.4666	Angan	24	145.95
Papuan Tip Cluster	39	150.4833	-10.5833	Angan	24	146.1166
Papuan Tip Cluster	39	149.9166	-10.5	Angan	24	146.3
Papuan Tip Cluster	39	150.8833	-9.9666	Angan	24	145.4666
Papuan Tip Cluster	39	150.4833	-10.2833	Angan	24	145.9833
Papuan Tip Cluster	39	151.2333	-10.0833	Angan	24	145.7166
Papuan Tip Cluster	39	150.9666	-10.1	Angan	24	145.8
Papuan Tip Cluster	39	149.85	-9.7833	Angan	24	145.8833
Papuan Tip Cluster	39	150.35	-9.4	Angan	24	146.1333
Papuan Tip Cluster	39	150.05	-10.4166	Angan	24	145.65
Papuan Tip Cluster	39	150.6166	-9.5	Angan	24	146.3
Papuan Tip Cluster	39	150.7166	-10.3166	Angan	24	146.5666
Papuan Tip Cluster	39	151	-9.6166	Angan	24	145.7833
Papuan Tip Cluster	39	151	-10	Angan	24	146.4
Papuan Tip Cluster	39	150.2166	-10.4333	Angan	24	146.2166
Papuan Tip Cluster	39	150.4166	-10.3333	Angan	24	146.4
Papuan Tip Cluster	39	150.3833	-10.3333	Angan	24	145.9166
Papuan Tip Cluster	39	150.05	-10.3833	Angan	24	145.6333

Sub group	id	Longitude	Latitude	Sub group	id	Longitude
Papuan Tip Cluster	39	150.8666	-9.8666	Angan	24	146.4333
Papuan Tip Cluster	39	149.9333	-10.5	Angan	24	146.4333
Papuan Tip Cluster	39	149.9	-9.7	Angan	24	146.0666
Papuan Tip Cluster	39	149.8	-9.7833	Angan	24	146.1166
Papuan Tip Cluster	39	150.8	-9.6833	Angan	24	145.9666
Papuan Tip Cluster	39	150.6833	-10.6166	Angan	24	145.45
Papuan Tip Cluster	39	150.1833	-10.4666	Angan	24	146.2333
Papuan Tip Cluster	39	150.5	-9.55	Angan	24	146.45
Papuan Tip Cluster	39	150.1166	-9.35	Angan	24	146.0333
Papuan Tip Cluster	39	150	-10.5	Angan	24	145.95
Papuan Tip Cluster	39	150.1	-10.65	Angan	24	145.95
Papuan Tip Cluster	39	150.4166	-10.3	Angan	24	146.2833
Papuan Tip Cluster	39	149.8	-10.2	Angan	24	145.8666
Papuan Tip Cluster	39	150.95	-10.0333	Angan	24	145.6166
Papuan Tip Cluster	39	150.8333	-9.4833	Angan	24	146.2333
Papuan Tip Cluster	39	150.4166	-10.7	Angan	24	145.9666
Papuan Tip Cluster	39	151.2833	-10.05	Angan	24	145.9333
Papuan Tip Cluster	39	150.8333	-10.6333	Angan	24	146.15
Papuan Tip Cluster	39	150.4333	-10.3	Angan	24	146.1
Papuan Tip Cluster	39	150.4833	-10.3333	Angan	24	146.05
Papuan Tip Cluster	39	150.9166	-9.6833	Angan	24	146.4166
Papuan Tip Cluster	39	150.45	-10.6833	Angan	24	145.9
Papuan Tip Cluster	39	149.8	-10.2333	Angan	24	145.9
Mek	8	139.7	-4.3666	Eleman	25	146
Mek	8	140.6666	-4.9	Eleman	25	146.3666
Mek	8	140.5333	-4.8	Eleman	25	145.8166
Mek	8	140.1	-4.65	Eleman	25	145.5
Mek	8	139.8666	-4.5666	Eleman	25	145.4166
Mek	8	139.8333	-4.45	Eleman	25	145.5333
Mek	8	140.0333	-4.5	Eleman	25	144.7
Mek	8	140.8	-4.6	Eleman	25	146.4
Mek	8	140	-4.75	Eleman	25	145.3166
Mek	8	139.8666	-4.6666	Eleman	25	145.4333
Mek	8	139.65	-4	Eleman	25	145.7833
Mek	8	140.3666	-4.7166	Eleman	25	146.2
Mek	8	139.8	-4.6	Eleman	25	144.6833

Sub group	id	Longitude	Latitude	Sub group	id	Longitude
Mek	8	139.75	-4.4666	Eleman	25	146.0833
Mek	8	140.2	-4.5833	Eleman	25	146.5333
Mek	8	139.7333	-4.8166	Eleman	25	145.55
Mek	8	140.6833	-4.7	Eleman	25	144.7666
Mek	8	140.3333	-4.7333	Eleman	25	145.3166
Mek	8	139.9166	-4.4166	Eleman	25	145.2666
Mek	8	140.05	-4.6333	Eleman	25	146.0666
Mek	8	140.3	-4.8666	Eleman	25	144.8333
Mek	8	139.9333	-4.4166	Eleman	25	144.85
Mek	8	140.0333	-4.75	Eleman	25	144.65
Asmat Kamoro	9	134.6666	-3.95	Eleman	25	145.3333
Asmat Kamoro	9	136	-4.55	Eleman	25	146.3833
Asmat Kamoro	9	136.8	-4.6666	Eleman	25	146.1
Asmat Kamoro	9	133.5333	-3.5833	Eleman	25	144.6
Asmat Kamoro	9	136.7	-4.8333	Eleman	25	145.45
Asmat Kamoro	9	137.1	-4.85	Eleman	25	146.2666
Asmat Kamoro	9	138.7833	-4.8666	Eleman	25	145.4166
Asmat Kamoro	9	134.9666	-3.95	Eleman	25	145.3166
Asmat Kamoro	9	133.2	-3.55	Eleman	25	145.9166
Asmat Kamoro	9	138.5833	-5.6666	Eleman	25	145.7
Asmat Kamoro	9	138.8833	-5.4666	Eleman	25	145.4333
Asmat Kamoro	9	135.8333	-4.4833	Eleman	25	145.6833
Asmat Kamoro	9	137.3333	-5.05	Eleman	25	145.1833
Asmat Kamoro	9	137.1833	-4.9833	Eleman	25	145.65
Asmat Kamoro	9	137	-4.75	Eleman	25	145.7166
Asmat Kamoro	9	136.4166	-4.5	Eleman	25	144.5833
Asmat Kamoro	9	133.4666	-3.4666	Eleman	25	145.2333
Asmat Kamoro	9	139.5333	-5.1833	Eleman	25	145.85
Asmat Kamoro	9	135.7166	-4.4666	Eleman	25	145.6333
Asmat Kamoro	9	135.0833	-4.4	Eleman	25	145.4
Asmat Kamoro	9	133.3833	-3.6	Eleman	25	145.4666
Asmat Kamoro	9	137.3166	-4.9	Eleman	25	145.8166
Asmat Kamoro	9	136.6	-4.6833	Eleman	25	146.3166
Asmat Kamoro	9	135.0166	-4.35	Eleman	25	144.6166
Asmat Kamoro	9	135.9	-4.25	Eleman	25	146.4333
Asmat Kamoro	9	136.6833	-4.4166	Eleman	25	145.35

Sub group	id	Longitude	Latitude	Sub group	id	Longitude
Asmat Kamoro	9	134.9333	-4.3	Eleman	25	145.65
Asmat Kamoro	9	136.8333	-4.7666	Turama Kikorian	26	144.05
Asmat Kamoro	9	135.9833	-4.55	Turama Kikorian	26	143.6
Asmat Kamoro	9	135.2	-4.45	Turama Kikorian	26	143.6
Asmat Kamoro	9	137.2833	-5.0166	Turama Kikorian	26	143.65
Asmat Kamoro	9	138.0666	-5.3833	Turama Kikorian	26	143.6
Asmat Kamoro	9	138.6833	-5.05	Turama Kikorian	26	143.6833
Asmat Kamoro	9	138	-5	Turama Kikorian	26	143.5
Asmat Kamoro	9	137.7	-5.1833	Turama Kikorian	26	144.1666
Asmat Kamoro	9	134.6833	-3.9666	Turama Kikorian	26	143.25
Asmat Kamoro	9	135.7666	-4.5	Turama Kikorian	26	144.0166
Asmat Kamoro	9	136.6833	-4.5333	Turama Kikorian	26	144.2166
Asmat Kamoro	9	136	-4.5333	Turama Kikorian	26	143.7666
Asmat Kamoro	9	138.4	-6.3	Turama Kikorian	26	143.6666
Asmat Kamoro	9	135.9166	-4.2666	Turama Kikorian	26	143.7
Asmat Kamoro	9	135.95	-4.3	Turama Kikorian	26	144.1333
Asmat Kamoro	9	136.7333	-4.5	Turama Kikorian	26	144.2833
Asmat Kamoro	9	139	-7.1833	Turama Kikorian	26	143.6833
Asmat Kamoro	9	136.4333	-4.7166	Turama Kikorian	26	144.2333
Asmat Kamoro	9	139.3	-5.7	Turama Kikorian	26	143.8166
Asmat Kamoro	9	139.5833	-4.9666	Turama Kikorian	26	143.4333
Asmat Kamoro	9	136.65	-4.3166	Turama Kikorian	26	143.5833
Asmat Kamoro	9	139.3666	-4.7333	Turama Kikorian	26	143.5166
Asmat Kamoro	9	136.2	-4.65	Turama Kikorian	26	143.5166
Awyu Domot	10	139.9166	-6.2333	Turama Kikorian	26	144.2333
Awyu Domot	10	139.9666	-6.9333	Turama Kikorian	26	143.9333
Awyu Domot	10	139.4166	-6.9	Turama Kikorian	26	143.95
Awyu Domot	10	139.15	-7.1333	Turama Kikorian	26	144.1333
Awyu Domot	10	140.5166	-6	Turama Kikorian	26	143.75
Awyu Domot	10	139.9333	-7.2333	Turama Kikorian	26	143.8166
Awyu Domot	10	139.9666	-7.2	Turama Kikorian	26	143.8166
Awyu Domot	10	139.4	-6.8	Turama Kikorian	26	143.9333
Awyu Domot	10	140.5	-6.0833	Turama Kikorian	26	143.1666
Awyu Domot	10	140.2833	-6.0833	Turama Kikorian	26	144.2
Awyu Domot	10	140.1833	-6.85	Turama Kikorian	26	143.65
Awyu Domot	10	140.35	-5.9	Turama Kikorian	26	143.15

Sub group	id	Longitude	Latitude	Sub group	id	Longitude
Awyu Domot	10	139.2166	-6.9166	Turama Kikorian	26	144.15
Awyu Domot	10	139.7666	-7.05	Turama Kikorian	26	144.1166
Awyu Domot	10	140.1666	-6.2666	Turama Kikorian	26	144.2
Awyu Domot	10	140.0166	-5.9166	Turama Kikorian	26	144.25
Awyu Domot	10	140.2833	-5.9333	Turama Kikorian	26	143.6666
Awyu Domot	10	139.5833	-7.0833	Turama Kikorian	26	144.1833
Awyu Domot	10	139.8	-5.0833	Turama Kikorian	26	143.7833
Awyu Domot	10	139.4666	-6.8	Turama Kikorian	26	143.0166
Awyu Domot	10	139.75	-7.1666	Turama Kikorian	26	143.7666
Awyu Domot	10	139.6166	-7.1666	Turama Kikorian	26	143.45
Awyu Domot	10	140.6666	-5.45	Turama Kikorian	26	143.8666
Awyu Domot	10	140.1833	-6.2166	Turama Kikorian	26	143.9166
Awyu Domot	10	139.5	-6.6333	Turama Kikorian	26	144.25
Awyu Domot	10	140.1666	-6.1666	Turama Kikorian	26	144.2
Awyu Domot	10	139.85	-6.9166	Turama Kikorian	26	143.9166
Awyu Domot	10	139.4	-6.7333	Kiwai Porome	27	143.7666
Awyu Domot	10	140.0333	-7.15	Kiwai Porome	27	143.8666
Awyu Domot	10	140.1666	-6.9166	Kiwai Porome	27	144.5
Awyu Domot	10	140.7166	-6.0666	Kiwai Porome	27	144.0833
Awyu Domot	10	140.7166	-5.5	Kiwai Porome	27	143.7666
Awyu Domot	10	140.4	-5.8	Kiwai Porome	27	144.4166
Awyu Domot	10	140.6	-5.7166	Kiwai Porome	27	144.1
Awyu Domot	10	140.4833	-6.15	Kiwai Porome	27	143.5833
Awyu Domot	10	140.4	-6.1666	Kiwai Porome	27	143.55
Awyu Domot	10	139.4	-6.7166	Kiwai Porome	27	143.25
Awyu Domot	10	140.5666	-5.9	Kiwai Porome	27	144.4333
Awyu Domot	10	140.2666	-6.85	Kiwai Porome	27	143.3833
Awyu Domot	10	140.3333	-6.6833	Kiwai Porome	27	143.5
Awyu Domot	10	139.8333	-6.2333	Kiwai Porome	27	143.3333
Awyu Domot	10	140.3	-5.1	Kiwai Porome	27	144.2833
Awyu Domot	10	140.05	-5.9166	Kiwai Porome	27	143.1833
Awyu Domot	10	139.8	-6.45	Kiwai Porome	27	143.8833
Awyu Domot	10	139.3833	-6.75	Kiwai Porome	27	144.1333
Awyu Domot	10	140.3166	-6.3166	Kiwai Porome	27	143.5833
Awyu Domot	10	140.5333	-5.8	Kiwai Porome	27	143.3666
Awyu Domot	10	140.6666	-6.1	Kiwai Porome	27	143.3833

Sub group	id	Longitude	Latitude	Sub group	id	Longitude
Awyu Domot	10	139.95	-6.2	Kiwai Porome	27	142.95
Awyu Domot	10	139.2166	-6.9833	Kiwai Porome	27	143.2
Ok	11	141.1166	-6.5166	Kiwai Porome	27	143.2666
Ok	11	141	-6.3333	Kiwai Porome	27	144.2833
Ok	11	141.2333	-5.3833	Kiwai Porome	27	144.1666
Ok	11	141.3333	-5.1666	Kiwai Porome	27	144.1833
Ok	11	141	-5	Kiwai Porome	27	143.6166
Ok	11	141.1333	-4.5833	Kiwai Porome	27	143.5833
Ok	11	141.2666	-5.3333	Kiwai Porome	27	144.2666
Ok	11	141.0333	-4.7666	Kiwai Porome	27	144.4833
Ok	11	141.25	-5.35	Kiwai Porome	27	143.25
Ok	11	141.0833	-5.05	Kiwai Porome	27	143.4666
Ok	11	141.7	-5.35	Kiwai Porome	27	144.25
Ok	11	141.2166	-5.4333	Kiwai Porome	27	144.3833
Ok	11	141.05	-5.0666	Kiwai Porome	27	144.3333
Ok	11	140.9833	-5.15	Kiwai Porome	27	144.1833
Ok	11	140.9	-5.75	Kiwai Porome	27	144.3833
Ok	11	141.2	-5.1166	Kiwai Porome	27	144.4666
Ok	11	141.2833	-4.5	Kiwai Porome	27	143.8333
Ok	11	141.6666	-5.1666	Kiwai Porome	27	144.3666
Ok	11	141.1833	-5.2833	Kiwai Porome	27	142.9666
Ok	11	141.0666	-5.3833	Kiwai Porome	27	143.25
Ok	11	141.0166	-4.7666	Kiwai Porome	27	144.25
Ok	11	141.6666	-5.1833	Kiwai Porome	27	144.5333
Ok	11	141.5	-4.5666	Kiwai Porome	27	144.15
Ok	11	141.0333	-5.6	Kiwai Porome	27	143.2666
Ok	11	141.1333	-5.6166	Kiwai Porome	27	143.1666
Ok	11	141.6	-5.1833	Kiwai Porome	27	144.1333
Ok	11	141.4333	-5.55	Kiwai Porome	27	143.2166
Ok	11	141.35	-5.2333	Gogodala Suki	28	142.2666
Ok	11	141.5	-5.5	Gogodala Suki	28	142.8666
Ok	11	141.0666	-5.4166	Gogodala Suki	28	142.4333
Ok	11	141.15	-4.8666	Gogodala Suki	28	141.2833
Ok	11	141.8	-5.5833	Gogodala Suki	28	142.9
Ok	11	141.1666	-4.8	Gogodala Suki	28	141.85
Ok	11	141.65	-5.0166	Gogodala Suki	28	142.9

Sub group	id	Longitude	Latitude	Sub group	id	Longitude
Ok	11	141.2	-4.8333	Gogodala Suki	28	142.45
Ok	11	141.5	-5.1166	Gogodala Suki	28	141.7
Ok	11	141.2833	-4.8666	Gogodala Suki	28	141.5333
Ok	11	141.05	-4.7333	Gogodala Suki	28	142.4666
Ok	11	140.8666	-5.7166	Gogodala Suki	28	141.8666
Ok	11	141.0833	-4.7333	Gogodala Suki	28	142.7666
Ok	11	141.0666	-4.7333	Gogodala Suki	28	142.95
Ok	11	141.05	-6.4333	Gogodala Suki	28	141.6666
Ok	11	141.1333	-4.8166	Gogodala Suki	28	142.9666
Ok	11	141.2166	-4.8666	Gogodala Suki	28	141.8333
Ok	11	141.05	-5.1	Gogodala Suki	28	141.5666
Ok	11	141.9666	-5.0666	Gogodala Suki	28	142.8333
Ok	11	141.1333	-4.6833	Gogodala Suki	28	141.7333
Ok	11	141.45	-4.3666	Gogodala Suki	28	142.85
Ok	11	141.0166	-4.7	Gogodala Suki	28	143.0166
Ok	11	141.2	-4.9166	Gogodala Suki	28	142.35
Marind	12	141.1166	-6.5166	Gogodala Suki	28	141.9833
Marind	12	141	-7	Gogodala Suki	28	142.5166
Marind	12	139.6	-7.9666	Gogodala Suki	28	141.7666
Marind	12	141.1833	-7.3666	Gogodala Suki	28	141.7166
Marind	12	139.2166	-8.0833	Gogodala Suki	28	142.9
Marind	12	139.6166	-7.3666	Gogodala Suki	28	141.3166
Marind	12	139.5	-7.8666	Gogodala Suki	28	142.7333
Marind	12	141.5	-7	Gogodala Suki	28	141.8666
Marind	12	139.7	-8.0666	Gogodala Suki	28	142.9833
Marind	12	139.8666	-7.5666	Gogodala Suki	28	142.5666
Marind	12	139.7	-7.6666	Gogodala Suki	28	142.4166
Marind	12	139.4666	-7.7666	Gogodala Suki	28	141.6833
Marind	12	139.3166	-6.5333	Gogodala Suki	28	142.9166
Marind	12	139.3333	-8.0333	Gogodala Suki	28	142
Marind	12	140.5	-7.4333	Gogodala Suki	28	141.6833
Marind	12	141.5666	-6.85	Gogodala Suki	28	142.7833
Marind	12	139.4666	-7.9	Gogodala Suki	28	142.6166
Marind	12	141.0666	-6.7833	Gogodala Suki	28	142.0333
Marind	12	141.3166	-7.5833	Gogodala Suki	28	142.95
Marind	12	139.9666	-7.7333	Gogodala Suki	28	141.7666

Sub group	id	Longitude	Latitude	Sub group	id	Longitude
Marind	12	140.1666	-8.0333	Gogodala Suki	28	142.8666
Marind	12	139.65	-7.7666	Gogodala Suki	28	142.9166
Marind	12	139.7166	-7.6666	Gogodala Suki	28	142.8666
Marind	12	140.7666	-8.1833	Gogodala Suki	28	142.9333
Marind	12	141.3333	-6.8	Gogodala Suki	28	142.45
Marind	12	140.55	-7.7166	Gogodala Suki	28	142.7
Marind	12	141.0333	-6.9666	Gogodala Suki	28	141.3333
Marind	12	140.6	-7.8666	Uhunduni	29	137.2833
Marind	12	139.9833	-7.9666	Uhunduni	29	137.4333
Marind	12	139.9333	-8.1166	Uhunduni	29	137.7
Marind	12	140.1666	-7.4666	Uhunduni	29	137.0833
Marind	12	140.15	-7.0166	Uhunduni	29	137
Marind	12	139.9333	-7.3	Uhunduni	29	137
Marind	12	139.65	-7.7166	Uhunduni	29	137.1333
Marind	12	139.8166	-7.2666	West TNG linkage	0	132.4333
Marind	12	140.2	-8.05	West TNG linkage	0	138.45
Marind	12	140.8166	-8.0833	West TNG linkage	0	139.4166
Marind	12	139.7166	-7.7	West TNG linkage	0	137.5
Marind	12	141.1666	-6.9833	West TNG linkage	0	135.8666
Marind	12	141.2666	-6.8	West TNG linkage	0	135.7666
Marind	12	141.3	-7.6	West TNG linkage	0	137.3833
Marind	12	140.2333	-8.35	West TNG linkage	0	135.9333
Marind	12	140.3	-7.2	West TNG linkage	0	138.7166
Marind	12	141.4833	-6.8833	West TNG linkage	0	135.9
Marind	12	139.6666	-7.5	West TNG linkage	0	139
Marind	12	139.65	-7.3	West TNG linkage	0	132.1166
Marind	12	140.45	-8.2	West TNG linkage	0	136.65
Marind	12	140.0166	-7.6666	West TNG linkage	0	136.9333
Marind	12	141.4333	-6.8333	West TNG linkage	0	137.35
Marind	12	141.55	-7.0333	West TNG linkage	0	136.25
Bosavi	15	142.4333	-6.6333	West TNG linkage	0	136.9166
Bosavi	15	142.35	-6.3166	West TNG linkage	0	138.3833
Bosavi	15	142.4166	-6.2833	West TNG linkage	0	135.3666
Bosavi	15	142.4	-6.65	West TNG linkage	0	132.25
Bosavi	15	142.3833	-6.3666	West TNG linkage	0	132.15
Bosavi	15	142.25	-6.45	West TNG linkage	0	136.2166

Sub group	id	Longitude	Latitude	Sub group	id	Longitude
Bosavi	15	142.2666	-6.25	West TNG linkage	0	135.5
Bosavi	15	142.4166	-6.1666	West TNG linkage	0	132.1333
Bosavi	15	143	-6.5833	West TNG linkage	0	131.9666
Bosavi	15	142.5666	-5.6833	West TNG linkage	0	138.75
Bosavi	15	142.4666	-6.3166	West TNG linkage	0	132.0833
Bosavi	15	142.3666	-6.2833	West TNG linkage	0	136.6833
Bosavi	15	142.9833	-6.35	West TNG linkage	0	138
Bosavi	15	142.4333	-6.2333	West TNG linkage	0	138.4666
Bosavi	15	142.35	-6.3666	West TNG linkage	0	136.5833
Bosavi	15	142.4333	-6.3666	West TNG linkage	0	132.1
Bosavi	15	142.3833	-6.65	West TNG linkage	0	136.75
Bosavi	15	142.45	-6.1833	West TNG linkage	0	138.4666
Bosavi	15	142.4166	-6.2333	West TNG linkage	0	132.6
Bosavi	15	142.4833	-6.2333	West TNG linkage	0	138.55
Bosavi	15	142.3333	-6.3166	West TNG linkage	0	136.2
Bosavi	15	143.1166	-6.6833	West TNG linkage	0	138.8166
Bosavi	15	142.7333	-6.4333	West TNG linkage	0	132.3166
Bosavi	15	142.6	-5.8666	West TNG linkage	0	137.9833
Bosavi	15	142.25	-6.3833	West TNG linkage	0	138.5666
Bosavi	15	142.35	-6.2833	West TNG linkage	0	138.8833
Bosavi	15	142.35	-6.25	West TNG linkage	0	138.4166
Bosavi	15	142.3666	-6.2	West TNG linkage	0	138.6666
Bosavi	15	142.6333	-6.4333	West TNG linkage	0	136.8
Bosavi	15	143.0833	-6.8	West TNG linkage	0	138.9833
Bosavi	15	142.4166	-6.3166	West TNG linkage	0	138.0833
Bosavi	15	142.3166	-6.2666	West TNG linkage	0	137.9166
Bosavi	15	142.35	-6.3833	West TNG linkage	0	136.3666
Bosavi	15	142.9666	-6.45	West TNG linkage	0	136.7166

References

1. Peterson AT. Predicting species' geographic distributions based on ecological niche modeling. The Condor. 2001;103(3):599 – 605.
2. Soberón J, Peterson T. Biodiversity informatics: Managing and applying primary biodiversity data. Philosophical Transactions of the Royal Society of London B: Biological Sciences. 2004;359(1444):689 – 698.

3. Banks WE, d'Errico F, Dibble HL, Krishtalka L, West D, Olszewski DI, et al. Eco-Cultural Niche Modeling: New Tools for reconstructing the Geography and Ecology of Past Human Populations. PaleoAnthropology. 2006;4:68 – 83.
4. Banks WE, d'Errico F, Peterson AT, Vanhaeren M, Kageyama M, Sepulchre P, et al. Human Ecological Niches and Ranges during the LGM in Europe derived from an Application of Eco-Cultural Niche modeling. Journal of Archaeological Science. 2008;35(2):481 – 491.
5. Banks WE, d'Errico F, Peterson AT, Kageyama M, Sima A, Sanchez-Goni MF. Neanderthal extinction by competitive exclusion. PLoS One. 2008;12(3):e3972.
6. Banks WE, Aubry T, d'Errico F, Zilhão J, Lira-Noriega A, Peterson AT. Eco-cultural niches of the Badegoulian: Unraveling Links between Cultural Adaptation and Ecology during the Last Glacial Maximum in France. Journal of Anthropological Archaeology. 2011;30(3):359 – 374.
7. Banks WE, d'Errico F, Zilhão J. Human-climate interaction during the Early Upper Paleolithic: Testing the hypothesis of an adaptive shift between the Proto-Aurignacian and the Early Aurignacian. Journal of Human Evolution. 2012;30(1):e17.
8. Banks WE, Antunes N, Rigaud S, d'Errico F. Ecological constraints on the first Prehistoric farmers in Europe. Journal of Archaeological Science. 2013;40(6):2746 – 2753.
9. Gillam JC, Anderson DG, Townsend A, Peterson AT. A Continental-scale Perspective on the Peopling of the Americas: Modeling Geographic Distributions and Ecological Niches of Pleistocene Populations. Current Research in the Pleistocene. 2007;24:86–90.
10. d'Errico F, Banks WE. Identifying Mechanisms behind Middle Paleolithic and Middle Stone Age Cultural Trajectories. Current Anthropology. 2013;0(0):p. S000.
11. d'Errico F, Banks WE, Warren DL, Sgubin G, van Niekerk K, Henshilwood C, et al. Identifying early modern human ecological niche expansions and associated cultural dynamics in the South African Middle Stone Age. Proceedings of the National Academy of Sciences USA. 2017;114(30):7869 – 7876.

12. Kondo Y. An ecological niche modelling of Upper Palaeolithic stone tool groups in the Kanto-Koshinetsu region, Eastern Japan. The Quaternary Research (Daiyonki-Kenkyu). 2015;54(5):207 – 218.

13. Tallavaara M, Luoto M, Korhonen N, Järvinen H, Seppä H. Human population dynamics in Europe over the Last Glacial Maximum. Proceedings of the National Academy of Sciences USA. 2015;112(27):8232 – 8237.

14. Antunes N, Banks WE, d'Errico F. Evaluating Viking eco-cultural niche variability between the Medieval Climate Optimum and the Little Ice Age: a feasibility study. In: Garcia et al. (eds), Debating Spatial Archaeology. Santander, 2012; 2014. p. 113–130. Available from: http://spatialarchaeology.wordpress.com/publications/.

15. Stockwell D. The Garp modeling system: Problems and solutions to automated spatial prediction. International Journal of Geographical Information Science. 1999;13(2):143 – 158.

16. Phillips SJ, Anderson RP, Schapire RE. Maximum entropy modeling of species geographic distributions. Ecological modeling. 2006;190(3):231 – 259.

17. Nix HA. A biogeographic analysis of Australian elapid snakes. In: Atlas of Elapid Snakes of Australia. (Ed.) R. Longmore. Australian Flora and Fauna Series. 1986;7:4–15.

18. Hirzel A, Hausser J, Chessel D, Perrin N. Ecological-niche factor analysis: how to compute habitat-suitability maps without absence data? Ecology. 2002;83(7):2027–2036.

19. Hastie T, Tibshirani R. Generalized additive models. London: Chapman and Hall; 1990.

20. McCullagh P, Nelder JA. Generalized linear models. In Monographs on Statistics and Applied Probability.; 1989.

21. Friedman JH. Multivariate Adaptive Regression Splines. The Annals of Statistics. 1991;19(1):1–67.

22. Burges CJC. A Tutorial on Support Vector Machines for Pattern Recognition. Data Mining and Knowledge Discovery. 1998;2(2):121–167. doi:10.1023/A:1009715923555.

23. Qiao H, Soberón J, Peterson AT. No silver bullets in correlative ecological niche modeling: Insights from testing among many potential algorithms for niche estimation. Methods in Ecology and Evolution. 2015;6(10):1126 – 1136.

24. Hernandez PA, Graham CH, Master LL, Albert DL. The effect of sample size and species characteristics on performance of different species distribution modeling methods. Ecography. 2006;29(5):773 – 785.

25. Stockwell DR, Peterson AT. Effects of sample size on accuracy of species distribution models. Ecological modeling. 2002;148(1):1 – 13.

26. Araùjo MB, New M. Ensemble forecasting of species distributions. Trends in Ecology & Evolution. 2007;22(1):42–47. doi:10.1016/j.tree.2006.09.010.

27. Thuiller W. Biodiversity: Climate change and the ecologist. Nature. 2007;448(7153):550 – 552.

28. Marmion M, Parviainen M, Luoto M, Heikkinen RK, Thuiller W. Evaluation of consensus methods in predictive species distribution modeling. Diversity and distributions. 2009;15(1):59 – 69.

29. Roura-Pascual N, Brotons L, Peterson AT, Thuiller W. Consensual predictions of potential distributional areas for invasive species: A case study of argentine ants in the Iberian peninsula. Biological Invasions. 2009;11(4):1017 – 1031.

30. Thuiller W, Georges D, Engler R. biomod2: Ensemble platform for species distribution modeling. R package version. 2013;2(7):r560.

31. Antunes N. Application d'algorithmes prédictifs à l'identification de niches écoculturelles des populations du passé: approche ethnoarchéologique. Université de Bordeaux. Pessac; 2015.

32. Renfrew C. Archaeology, genetics and linguistic diversity. Man. 1992;27(3):445 – 478.

33. Pawley A, Ross M. Austronesian historical linguistics and culture history. Annual review of Anthropology. 1993;22(1):425 – 459.

34. Bellwood P. An archaeologist's view of Language macrofamily relationships. Oceanic Linguistics. 1994;33(2):391 – 406.

35. Hurles ME, Matisoo-Smith E, Gray RD, Penny D. Untangling Oceanic settlement: The edge of the knowable. Trends in Ecology & Evolution. 2003;18(10):531 – 540.

36. Dunn M, Terrill A, Reesink G, Foley RA, Levinson SC. Structural phylogenetics and the reconstruction of ancient language history. Science. 2005;309(5743):2072 – 2075.

37. Gray RD, Drummond AJ, Greenhill SJ. Language phylogenies reveal expansion pulses and pauses in pacific settlement. Science. 2009;323(5913):479 – 483.

38. Kayser M. The human genetic history of Oceania: Near and remote views of dispersal. Current Biology. 2010;20(4).

39. O'Connell JF, Allen J. The process, biotic impact, and global implications of the human colonization of Sahul about 47,000 years ago. Journal of Archaeological Science. 2015;47:73 – 84.

40. Greenberg JH. The indo-pacific hypothesis. Current Trends in Linguistics. 1971;8:809 – 871.

41. Wurm SA, Laycock DC, Voorhoeve CL, Dutton TE. Papuan linguistic prehistory, and past language migrations in the New Guinea area. New Guinea area languages and language study. 1975;1:935 – 960.

42. Foley WA. The Papuan languages of New Guinea. Cambridge: University Press; 1986.

43. Foley WA. The languages of New Guinea. Annual Review of Anthropology. 2000;languages.

44. Ross M. Pronouns as a preliminary diagnostic for grouping Papuan languages. In: Pawley A, Attenborough RGJHR, editors. Investigations into the cultural, linguistic and biological history of the Papuan speaking peoples. Canberra: Pacific Linguistics; 2005. p. 15 – 66.

45. Greenhill SJ. Transnewguinea.Org: An online database of New Guinea languages. PloS One. 2015;10(10):e0141563.

46. Reesink G, Singer R, Dunn M. Explaining the linguistic diversity of Sahul using population models. PLoS Biology. 2009;7(11):e1000241.

47. Blust R. An Austronesianist looks at Sino-Austronesian. Journal of Chinese Linguistics Monograph Series. 1995; p. 283 – 298.

48. Bellwood P. The Austronesian dispersal. Newsletter of Chinese Ethnology. 1997;35:1 – 26.

49. Kirch PV. The Lapita peoples: Ancestors of the oceanic world (The Peoples of South-East Asia and the Pacific). Oxford: Blackwell: Malden (MA); 1997.

50. Pawley A. The Austronesian dispersal: Languages, technologies and people. In: Bellwood P, Renfrew C, editors. Examining the farming/language dispersal hypothesis. Cambridge: McDonald Institute for Archaeological Research; 2002. p. 251 – 273.

51. Blench R, Sagart L, Sanchez-Mazas A. The peopling of East Asia: Putting together Archaeology, Linguistics and Genetics. New York: Routledge: London; 2005.

52. Gordon RG, Grimes BF. Ethnologue: Languages of the world; 2005. Available from: http://www.ethnologue.com.

53. Hammarström H, Forkel R, Haspelmath M. Glottolog 3.0. Jena: Max Planck Institute for the Science of Human History (available online accessed on 2017-05-15). Science. 2017;.

54. Durie M, Ross M, Eds. The comparative method reviewed: Regularity and irregularity in language change. Oxford: Oxford University Press; 1996.

55. Gibbons JR, Clunie FG. Sea level changes and pacific prehistory: New insight into early human settlement of Oceania. The Journal of Pacific History. 1986;21(2):58 – 82.

56. O'Connell JF, Allen J. Pre-LGM Sahul (Pleistocene Australia-New Guinea) and the Archaeology of Early Modern Humans. In: Mellars P, Boyle K, Bar-Yosef O, Stringer C, editors. Rethinking the human revolution: New behavioural and biological perspectives on the origin and dispersal of modern humans, McDonald Institute for Archaeological Research. Oxford: Oxbow Books; 2007. p. 395 – 410.

57. Pawley A, Green R. Dating the dispersal of the oceanic languages. Oceanic Linguistics. 1973;1/2(12):1 – 67.

58. Bulmer S. Settlement and economy in prehistoric Papua New Guinea: A review of the archeological evidence. Journal de la Société des Océanistes. 1975;31(46):7 – 75.

59. White JP, O'Connell JF, Koettig M. A Prehistory of Australia, New Guinea, and Sahul. Academic Press. 1982;.

60. Swadling P. How long have people been in the Ok Tedi Impact Region? Boroko: PNG National museum; 1983.

61. Spriggs M. The Lapita cultural complex: Origins, distribution, contemporaries and successors. The Journal of Pacific History. 1984;19(4):202 – 223.

62. Groube L, Chappell J, Muke J, Price D. A 40,000 year-old human occupation site at Huon peninsula, Papua New Guinea. Nature. 1986;324(6096):453 – 455.

63. Bellwood P, Koon P. "Lapita colonists leave boats unburned!" The question of Lapita links with island Southeast Asia. Antiquity. 1989;63(240):613 – 622.

64. O'Connell JF, Allen J. Dating the colonization of Sahul (Pleistocene Australia-New Guinea): A review of recent research. Journal of Archaeological Science. 2004;31(6):835 – 853.

65. Leavesley MG, Bird MI, Fifield LK, Hausladen P, Santos G, Tada MD. Buang Merabak: Early evidence for human occupation in the Bismarck archipelago, Papua New Guinea. Australian Archaeology. 2002;54(1):55 – 57.

66. Schiefenhövel W. Human ethological perspectives on prehistoric adaptation and dispersal in the Central Highlands of New Guinea. In: Sanz N, editor. Human evolution, adaptation and social developments. France: UNESCO; 2014. p. 235–254. (World Heritage Programs, 39).

67. Swadling P, Wiessner P, Tumu A. Prehistoric stone artefacts from Enga and the implication of links between the Highlands, Lowlands and Islands for early agriculture in Papua New Guinea. Journal de la Société des Océanistes. 2008;1(126-127):271 – 292.

68. McElhanon KA, Voorhoeve CL. The Trans-New Guinea phylum: Explorations in deep-level genetic relationships. Canberra: The Australian National University; 1970.

69. Golson J. Archaeology and agricultural history in the New Guinea Highlands. In: Problems in Economic and Social Archaeology. London: Duckworth; 1976. p. 201–220.

70. Golson J. [Transitions to Agriculture in the Pacific Region] The New Guinea Highlands on the eve of agriculture. Bulletin of the Indo-Pacific Prehistory Association. 1991;11:82–91.

71. Pawley A. The chequered career of the Trans New Guinea hypothesis: recent research and its implications. Papuan pasts: Cultural, linguistic and biological histories of Papuan-speaking peoples. 2005; p. 67–108.

72. Ross M. Pronouns as a preliminary diagnostic for grouping Papuan languages. In: Pawley A GJHR Attenborough R, editor. Investigations into the cultural, linguistic and biological history of the Papuan speaking peoples. Pacific Linguistics: Canberra; 2005. p. 15–66.

73. Denham T, Haberle SG, Lentfer C, Fullagar R, Field J, Therin M, et al. Origins of agriculture at Kuk swamp in the Highlands of New Guinea. Science. 2003;301(5630):189 – 193.

74. Denham T. The roots of agriculture and arboriculture in New Guinea: Looking beyond Austronesian expansion, Neolithic packages and indigenous origins. World Archaeology. 2004;36(4):610 – 620.

75. Bergström A, Nagle N, Chen Y, McCarthy S, Pollard MO, Ayub Q, et al. Deep Roots for Aboriginal Australian Y Chromosomes. Current Biology. 2016;26(6):809 – 813.

76. Bergström A, Oppenheimer SJ, Mentzer AJ, Auckland K, Robson K, Attenborough R, et al. A Neolithic expansion, but strong genetic structure, in the independent History of New Guinea. Science. 2017;357(6356):1160 – 1163.

77. Malaspinas AS, Westaway MC, Muller C, Sousa VC, Lao O, Alves I, et al. A genomic history of aboriginal australia. Nature. 2016;538(7624):207.

78. Bellwood P. Prehistory of the Indo-Malaysian Archipelago: Revised edition. Sydney: Academic Press; 1985.

79. Bellwood P. The Archaeology of Papuan and Austronesian Prehistory in the Northern Moluccas, Eastern Indonesia. In: Blench R, Spriggs M, editors. Archaeology and language II. Archaeological Data and Linguistic Hypotheses. London: Routledge; 1998. p. 128 – 140.

80. Bellwood P. From bird's head to bird's eye view: Long term structures and trends in Indo-Pacific Prehistory. In: Miedema J, Odé C, Dam

RAC, editors. Perspectives on the Bird's Head of Irian Jaya, Indonesia, Proceedings of the Conference Leiden, 13-17 October 1997. Amsterdam, Atlanta: Editions Rodopi; 1998. p. 951 – 975.

81. Ross M. Sequencing and dating linguistic events in Oceania: The linguistics/archaeology interface. Blench R., Spriggs M. (Eds.). Archaeology and language II. Archaeological Data and Linguistic Hypotheses. London: Routledge; 1998.

82. Spriggs M. The Island Melanesians. Oxford: Blackwell; 1997.

83. Spriggs M, Blench R. From Taiwan to the Tuamotus: absolute dating of Austronesian language spread and major subgroups. Archaeology and language II: archaeological data and linguistic hypotheses. 1998; p. 115–127.

84. Solheim WG. The Nusantao hypothesis: The origin and spread of Austronesian speakers. Asian perspectives. 1984;26(1):77 – 88.

85. Kayser M, Brauer S, Weiss G, Underhill P, Roewer L, Schiefenhövel W, et al. Melanesian origin of Polynesian y-chromosomes. Current Biology. 2000;10(20):1237 – 1246.

86. Allen J. From Beach to Beach: The Development of Maritime Economies. In: O'Connor S, Veth P, editors. Prehistoric Melanesia East of Wallace's Line: Studies of past and present maritime cultures of the Indo-Pacific region, Modern Quaternary Research in Southeast Asia. Rotterdam: Barkema; 2000. p. 139 – 176.

87. Hurles ME, Nicholson J, Bosch E, Renfrew C, Sykes BC, Jobling MA. Y-chromosomal evidence for the origins of oceanic-speaking peoples. Genetics. 2002;160(1):289 – 303.

88. Green R. The Lapita horizon and traditions-signature for one set of oceanic migrations. In: Sand C, editor. Pacific Archaeology: Assessments and Prospects. Noumea: Service des Musées et du Patrimoine de Nouvelle-Calédonie; 2003. p. 95 – 120.

89. Blust R. Eastern Malayo-Polynesian: A subgrouping argument. In: Wurm SA, Carrington L, editors. Second international conference on Austronesian linguistics: Proceedings, volume 1, Department of Linguistics. Canberra: Pacific Linguistics; 1978. p. 181 – 234.

90. Blust R. More on the position of the languages of Eastern Indonesia. Oceanic Linguistics. 1983;22(1/2):1 – 28.

91. Friedlaender JS, Friedlaender FR, Reed FA, Kidd KK, Kidd JR, Chambers GK, et al. The genetic structure of pacific islanders. PLoS Genetics. 2008;4(1):e19.

92. de Souza Muñoz ME, De Giovanni R, de Siqueira MF, Sutton T, Brewer P, Pereira RS, et al. OpenModeller: a generic approach to species' potential distribution modelling. GeoInformatica. 2011;15(1):111–135.

93. Allouche O, Tsoar A, Kadmon R. Assessing the accuracy of species distribution models: prevalence, kappa and the true skill statistic (TSS). Journal of Applied Ecology. 2006;43(6):1223–1232. doi:10.1111/j.1365-2664.2006.01214.x.

94. DeLong ER, DeLong DM, Clarke-Pearson DL. Comparing the areas under two or more correlated receiver operating characteristic curves: a nonparametric approach. Biometrics. 1988; p. 837–845.

95. Fielding AH, Bell JF. A review of methods for the assessment of prediction errors in conservation presence/absence models. Environmental Conservation. 1997; p. 38–49.

96. Dodd LE, Pepe MS. Partial AUC estimation and regression. Biometrics. 2003;59(3):614–623.

97. Peterson AT, Papeş M, Soberón J. Rethinking receiver operating characteristic analysis applications in ecological niche modeling. Ecological Modelling. 2008;213(1):63–72. doi:10.1016/j.ecolmodel.2007.11.008.

98. Muturzikin. Muturzikin Linguistic Maps, http://www.muturzikin.com/. Québec,Canada: Rouyn-Noranda; 2005. Available from: `http://www.muturzikin.com/`.
