# Supplementary material for: Quantitative methods demonstrate that environment alone is an insufficient predictor of present-day language distributions in New Guinea
Source: PLoS One. 2020 Oct 7;15(10):e0239359. doi: 10.1371/journal.pone.0239359 (PMC7540881; doi:10.1371/journal.pone.0239359)
Supplement: S2 File — (PDF) [file pone.0239359.s003.pdf]

S3 File. Supporting Information for:

Quantitative methods demonstrate that environment alone is an insufficient predictor of present-day language distributions in New Guinea

Nicolas Antunes, Wulf Schiefenhövel, Francesco d'Errico, William E. Banks, Marian Vanhaeren

Correspondence to: antunes@rgzm.de

Environmental characteristics

At a broad scale and relative to the whole of Australasia, NG ecosystems might appear homogeneous (S3 Fig. 1), but at finer scales it is possible to distinguish significant differences (Fig. 8). As recommended by Segurado *et al.* (2006) [1], selecting and using non-correlated environmental variables should improve niche estimations (reduce overfitting) and facilitate interpretations. However, problems associated with employing correlated variables are minimized with an 'ensemble' or consensus modeling approach. Firstly, the multiple employed algorithms do not treat all variables equally and many, such as GARP (genetic rules), exclude redundant information. In addition, Feng et al. [2] selecting and using non-correlated environmental variables should improve niche estimations (reduce overfitting) and facilitate interpretations. However, problems associated with employing correlated variables are minimized with an 'ensemble' or consensus modeling approach. Firstly, the multiple employed algorithms do not treat all variables equally and many, such as GARP (genetic rules), exclude redundant information. In addition, Feng et al.2) demonstrate that excluding highly correlated predictor variables from modelling performed with Maxent does not significantly influence model performance. Secondly, variability between two variables may be distinguishable locally (at the ecosystem scale), whereas the same variables appear to be correlated at a coarser, macro-regional scale (S3 Fig. 2).

Correlation between pairs of variables

Differences in correlations between environmental variables have been recognized for the four principal ecosystems: Vogelkop montane rain forests, Southeastern Papuan rain forests, Northern New Guinea lowland rainforests and freshwater swamp forests, and the Trans Fly savanna and grasslands (S3 Fig. 2 C). These four ecosystems show different correlations between the available environmental variables. This correlative variability between the different ecosystems warrants that all 26 variables be retained for estimating ELNs.

Local variations of the correlation rates among the most auto-correlated variables are depicted in S3 Fig. 2 D. These maps show that correlations are not homogeneous across ecosystems. Although in some cases the same correlations are shared amongst the ecosystems, one notes that they do not always concern the same geographic regions. The southern region characterized by

S3 Fig. 1: **Biomes of Australasia according to the World Wide Fund For Nature (WWF) ecosystem database [3].** Map generated with R::rgdal package - CRAN repository.

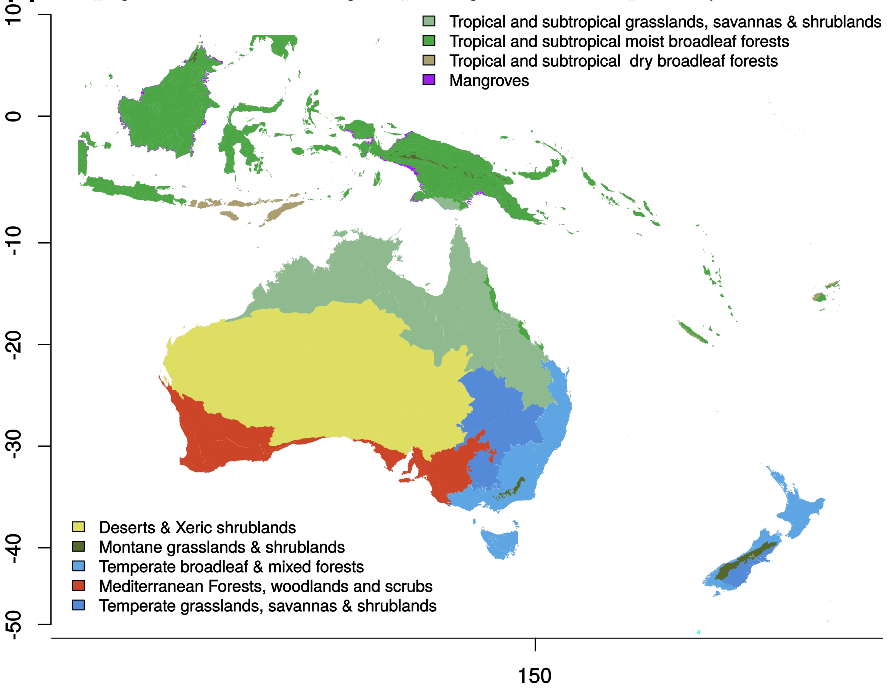

the Trans Fly savanna and grasslands ecosystem does not follow the same pattern as the rest of the archipelago.

Estimation of overfitting for an ecosystem

In order to estimate potential overfitting with respect to a given ecosystem (the Southeastern Papuan rain forests ecosystem), we performed a model with all 26 environmental variables and another with non-auto-correlated variables, each using the Roro occurrence data set and the Maxent.

The ENMTools MDS dimensional analysis [4] obtained for this ecosystem (S3 Fig. 3 A) differs from the MDS obtained for all NG (S3 Fig. 2 A).Variations in the correlations can also be seen in the heatmap comparisons (S3 Fig. 2 B and C).

Based on the MDS for this ecosystem, 15 variables were retained to perform the non-auto-correlated modelling (S3 Fig. 3 C-A). Results of both modelling are depicted in S3 Fig. 3 C-D. Niche modeling done with the 26 environmental variables shows a tendency towards more restricted prediction around occurrences, and this is likely due to overfitting (S3 Fig. 3 B-D).

The ENMevaluate function of the R::ENMeval package [5] allows one to obtain differences in the AUC (area under the receiver operating characteristic curve) by executing Maxent across a range of settings for each of the two mod-

S3 Fig. 2: **Variable correlations.** ENMTools MDS plot [4] for all NG (A); heatmap of variable correlation values for all NG (B); heatmaps of variable correlations for 4 distinct ecosystems (the WWF ecosystem database [3]) (C); geographic variation of correlation values for 4 pairs of auto-correlated variables (D).

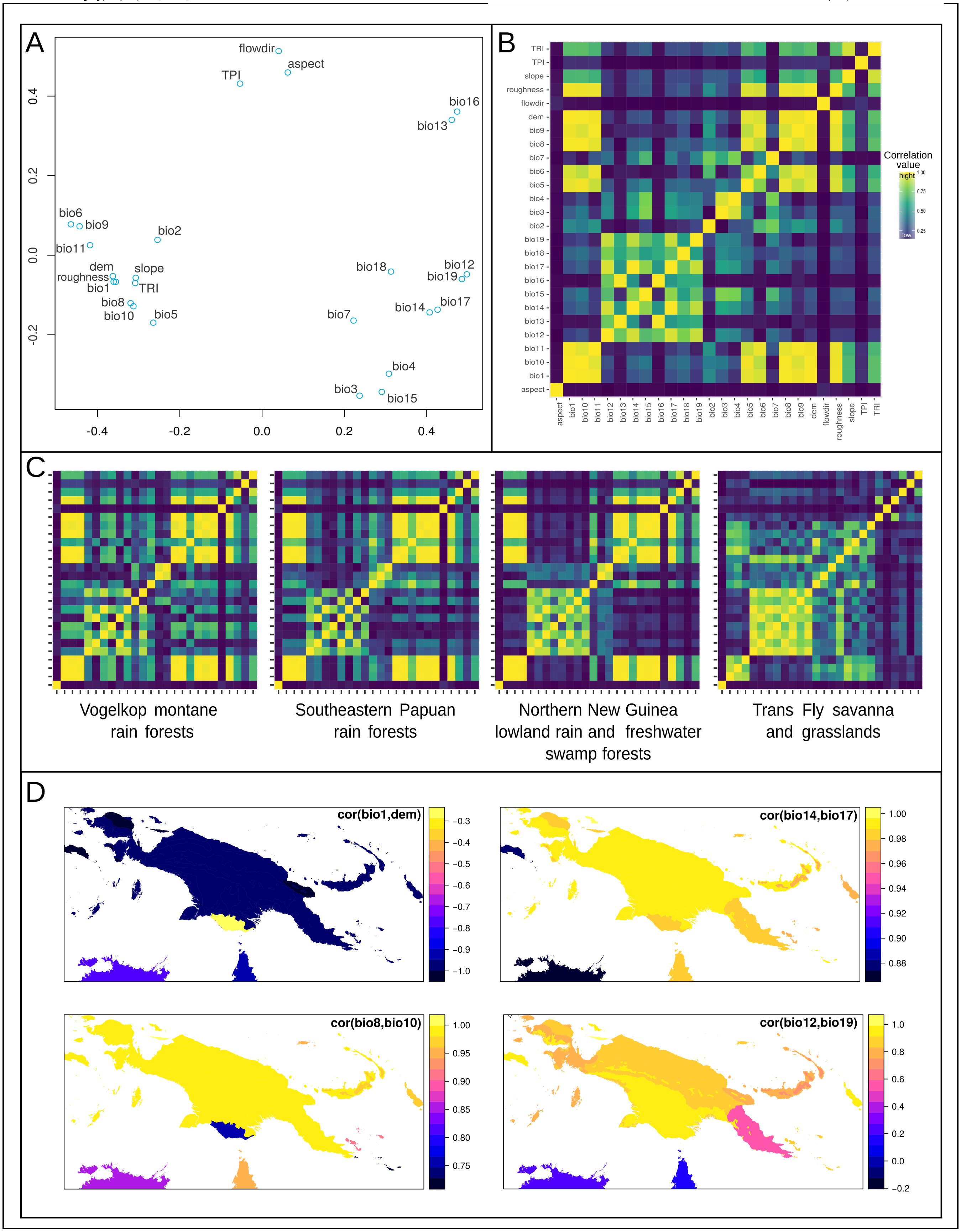

S3 Fig. 3: **Overfitting estimation.** ENMTools MDS plot for the Southeastern Papuan rain forests ecosystem (A); variables colored in black are those retained for the non-auto-correlated modeling; Roro language occurrences (B); modelling with 26 variables (C); non-auto-correlated modeling 15 variables (D)

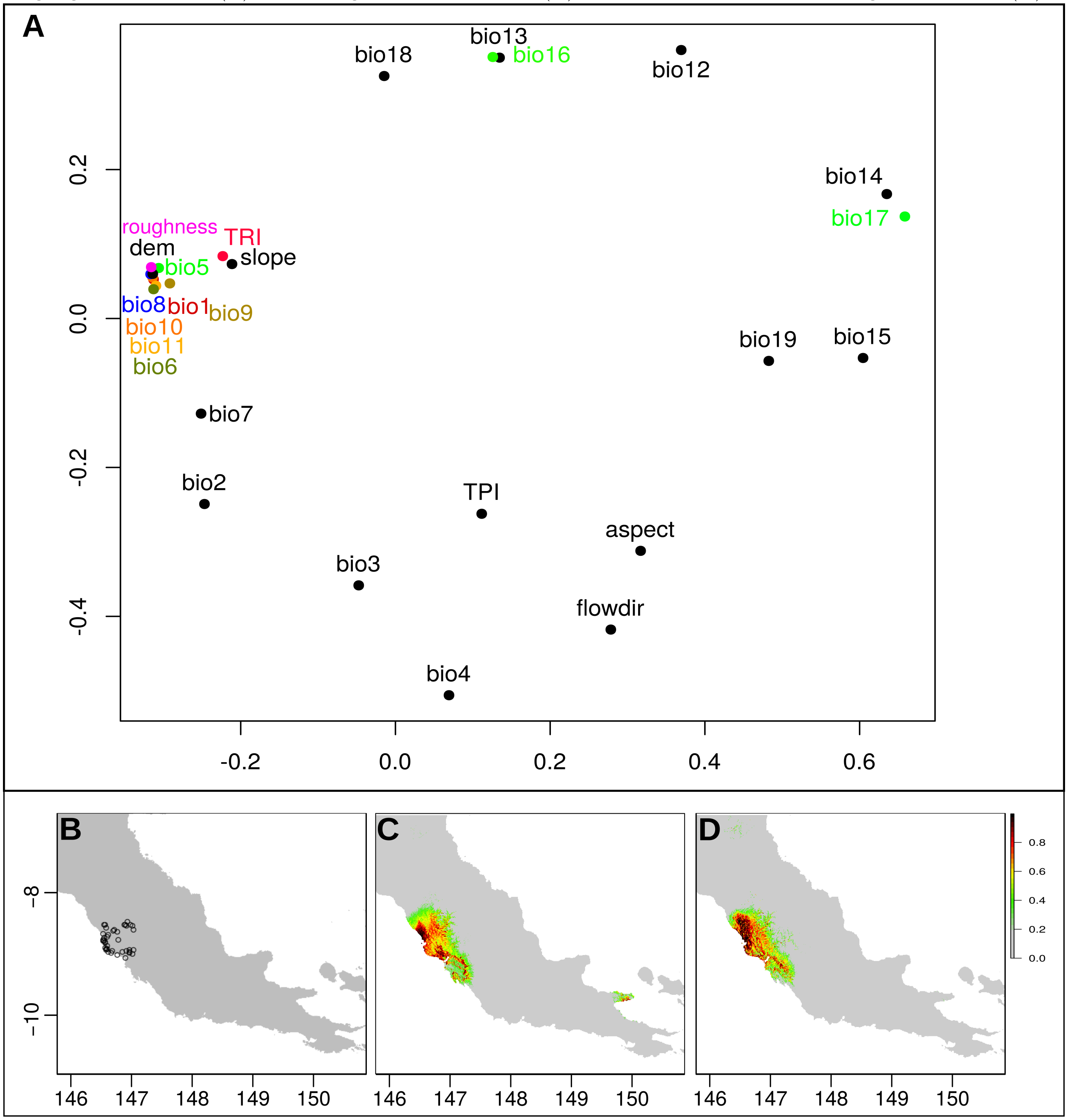

S3 Table 1: **Difference in AUC (area under the receiver operating characteristic curve) between modeling runs that used auto-correlated variables and those that did not.** Maxent was run 12 times for the non-auto-correlated modelling (15 variables) and the modelling with all predictor variables; run settings differ according to linear feature (L), quadratic feature (Q), and values of the regularization multiplier (rm). Results are extracted from the output of the ENMevaluate function in the R::ENMeval package [5].The random K-fold method was used for data partitioning and employed 5 bins.

Features	rm	avg.diff.AUC 15 variables	avg.diff.AUC 26 variables
L	0.5	0.0132	0.0161
Q	0.5	0.0136	0.0129
LQ	0.5	0.0153	0.0178
L	1	0.0126	0.0143
Q	1	0.0133	0.0119
LQ	1	0.0140	0.0156
L	1.5	0.0128	0.0116
Q	1.5	0.0134	0.0097
LQ	1.5	0.0140	0.0136
L	2	0.0133	0.0095
Q	2	0.0138	0.0084
LQ	2	0.0139	0.0122

eling scenarios. The greater the difference, the greater the overfitting [6].The average of these differences is 0.0128 and 0.0135 for the two models, respectively (S3 Table 1).

Retention of all the 26 variables

In the light of the geographic differences between results obtained with auto-correlated variables and those obtained using only non-auto-correlated variables, and considering the fact that overfitting is minimized by the consensus approach, we decided to retain all the available bioclimatic variables, even if some were, on average, highly correlated. Moreover, due to the use of multiple predictive architectures within a consensus approach, we were able to take into account local variability when it is relevant to eco-linguistic niches (i.e., based on their AUC and accuracy values, as well as their partial-ROC ratio). Retaining all available environmental variables allows one to compare the niches with each other (for example to represent them in the same PCA in order to evaluate their relative positions in the environmental space).

References

1. Segurado P, Araújo MB, Kunin W. Consequences of spatial autocorrelation for niche-based models. Journal of Applied Ecology. 2006;43(3):433–

444.

2. Feng X, Park DS, Liang Y, Pandey R, Papeş M. Collinearity in ecological niche modeling: Confusions and challenges. Ecology and evolution. 2019;9(18):10365–10376.
3. Oviedo G, Larsen PB, Maffi L. Indigenous and traditional peoples of the world and ecoregion conservation: An integrated approach to conserving the world's biological and cultural diversity. WWF (World Wide Fund For Nature) International; 2000.
4. Warren DL, Glor RE, Turelli M. ENMTools: a toolbox for comparative studies of environmental niche models. Ecography. 2010;33(3):607–611.
5. Muscarella R, Galante PJ, Soley-Guardia M, Boria RA, Kass J, Uriarte M, et al. ENMeval: An R package for conducting spatially independent evaluations and estimating optimal model complexity for ecological niche models. Methods in Ecology and Evolution. 2014;5(11):1198–1205.
6. Warren DL, Seifert SN. Ecological niche modeling in Maxent: the importance of model complexity and the performance of model selection criteria. Ecological applications. 2011;21(2):335–342.
